# Trinucleotide substrates under pH–freeze–thaw cycles enable open-ended exponential RNA replication by a polymerase ribozyme

**DOI:** 10.1038/s41557-025-01830-y

**Published:** 2025-05-28

**Authors:** James Attwater, Teresa L. Augustin, Joseph F. Curran, Samantha L. Y. Kwok, Luis Ohlendorf, Edoardo Gianni, Philipp Holliger

**Affiliations:** 1 MRC Laboratory of Molecular Biology, Cambridge Biomedical Campus, Cambridge, UK; 2 UCL Department of Chemistry, London, UK; 3 Present Address: Merkin Institute of Transformative Technologies in Healthcare, Broad Institute of MIT and Harvard, Cambridge, MA USA; 4 Present Address: Department of Chemistry and Chemical Biology, Harvard University, Cambridge, MA USA; 5 Present Address: Howard Hughes Medical Institute, Harvard University, Cambridge, MA USA; 6 Present Address: The Francis Crick Institute, London, UK

**Keywords:** RNA, Origin of life

## Abstract

RNA replication is considered a key process in the origins of life. However, both enzymatic and non-enzymatic RNA replication cycles are impeded by the ‘strand separation problem’, a form of product inhibition arising from the extraordinary stability of RNA duplexes and their rapid reannealing kinetics. Here we show that RNA trinucleotide triphosphates can overcome this problem by binding to and kinetically trapping dissociated RNA strands in a single-stranded form, while simultaneously serving as substrates for replication by an RNA polymerase ribozyme. When combined with coupled pH and freeze–thaw cycles, this enabled exponential replication of both (+) and (−) strands of double-stranded RNAs, including a fragment of the ribozyme itself. Subjecting random RNA sequence pools to open-ended replication yielded either defined replicating RNA sequences or the gradual emergence of diverse sequence pools. The latter derived from partial ribozyme self-replication alongside generation of new RNA sequences, and their composition drifted towards hypothesized primordial codons. These results unlock broader opportunities to model primordial RNA replication.

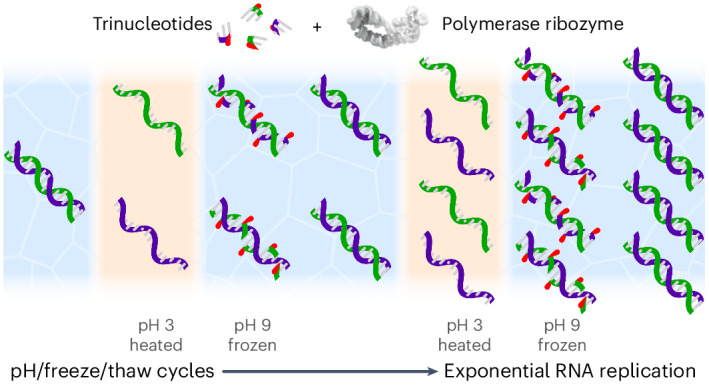

## Abstract (web summary)

Models of abiotic RNA replication suffer from inherent product inhibition arising from the high stability of RNA duplexes. Now, it has been shown that RNA trinucleotide substrates undergoing simple physicochemical cycles can overcome this problem, enabling open-ended replication of defined and random RNA sequences by a polymerase ribozyme.

## Main

**Fig. 1:**
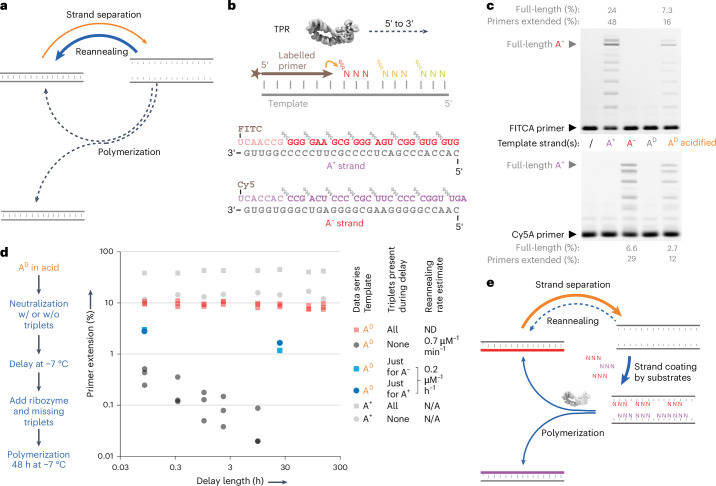
Triplet substrates alleviate strand reannealing during RNA replication. **a**, The strand separation problem: the high energetic barrier of strand separation and speed of strand reannealing jointly inhibit RNA replication cycles. **b**, RNA strand copying by polymerization of trinucleotide triphosphates (triplets) upon a RNA template, catalysed by a TPR (a polymerase ribozyme using trinucleotide triphosphates as substrates, structure from ref. ^21^). Below, substrates for synthesis of the A^D^ RNA duplex. Individual strands (A^+^ and A^−^) are shown hybridized to their complementary primers and triplets. **c**, TPR-catalysed RNA polymerization using 0.1 µM A^D^ duplex or individual strands (A^+^, A^−^) as templates, showing product A^−^ (top, fluorescein channel) and A^+^ (bottom, Cy5 channel). ‘A^D^ acidified’ was pre-incubated in 2.5 mM HCl, and neutralized before reactions were frozen to initiate RNA polymerization (−7 °C for 48 h). Observed percentages of primer extended by >1 triplet, or reaching full length, are given after subtraction of levels in no-template controls (/). **d**, Effect of delaying ribozyme and triplet/primer addition after neutralization of the acidified A^D^ template upon the percent of primers extended. Curve fitting indicates that A^D^ reanneals with a *t*_1/2_ of 0.7 µM^−1^ min^−1^ (black circles, *n* = 3). Addition of triplets immediately upon A^D^ neutralization (red squares, *n* = 3) essentially abolishes strand reannealing. ND, not detected. **e**, Revised scheme of an RNA replication cycle driven by triplet substrate inhibition of strand reannealing. Source data

Life on Earth relies on the faithful copying of its genetic material—replication—to enable heredity and evolution. This process is thought to have begun with the templated polymerization of activated mono- or oligonucleotide building blocks by chemical replication processes^1–3^ and later by RNA-catalysed RNA replication^4–7^. In its simplest form, RNA replication comprises the copying of (+) and (−) strands into complementary (−) and (+) daughter strands. For replication to proceed further, the double-stranded RNA replication products (duplexes) must again be dissociated into single-stranded RNAs, and these must be copied before they reanneal (Fig. 1a).

However, RNA duplexes of functional lengths and concentrations (for example >25 nucleotides (nt), 100 nM) behave as essentially inert, ‘dead-end’ products due to their remarkable stability (with melting temperatures approaching the boiling point of water)^8^. Furthermore, even when dissociated into individual, single-stranded RNAs, such strands reanneal on timescales (seconds to minutes) that are shorter than the typical time needed for copying reactions (hours to days) either by non-enzymatic processes or by polymerase ribozymes^1^. Thus, RNA replication cycles under standard conditions are both kinetically and thermodynamically disfavoured (Fig. 1a).

This so-called ‘strand separation problem’^1^ is aggravated by the comparative chemical instability of RNA. This precludes duplex dissociation under harsh conditions. High temperatures degrade RNA templates and ribozyme catalysts, particularly in the presence of divalent cations such as Mg^2+^ (which boost ribozyme activity but accelerate RNA fragmentation by transesterification)^9^. Furthermore, the strand separation problem worsens with increasing lengths of RNA duplexes, which become progressively harder to dissociate, more vulnerable to degradation and more prone to reannealing.

A range of different approaches have been tried to overcome this fundamental barrier to open-ended RNA replication. Acidic pH can protonate the N1 of adenine and N3 of cytosine, disrupting base-pairing and destabilizing RNA duplexes^10^. Coupled with wet/dry cycles or ionic gradients in a thermophoretic setting, this has been shown to promote duplex melting and RNA assembly, and enable nucleic acid amplification by proteinaceous enzymes^11–14^. Furthermore, highly viscous solvents can slow RNA reannealing sufficiently for long (32 nt) substrates to be ligated^15,16^. Alternatively, strand-displacement syntheses can circumvent full duplex dissociation by the progressive addition of ‘invader’ oligonucleotides complementary to the non-templating strand^17^, or by the buildup of conformational strain on circular RNA templates^18^. Nevertheless, the scope of RNA-catalysed RNA replication cycles has been limited to polymerization of mononucleotides on primers flanking a 4-nt region assisted by denaturants^19^, or the templated ligation of up to three polynucleotide substrate segments^14,20^. However, general RNA replication and open-ended evolution requires the replication of longer sequences (able to encode a phenotype) via the polymerization of building blocks short enough to allow free sequence variation.

In this Article we describe an approach that unlocks both the replication of longer RNA sequences and enables free sequence variation in replicating RNA pools. Our approach leverages an unexpected capacity of trinucleotide triphosphate (triplet) substrates to stabilize dissociated RNA strands. This can be coupled to cycles of pH, temperature and concentration to drive open-ended RNA replication by a polymerase ribozyme that utilises triplet substrates.

## Results and discussion

### Inhibition of strand reannealing by triplet substrates

**Extended Data Fig. 1:**
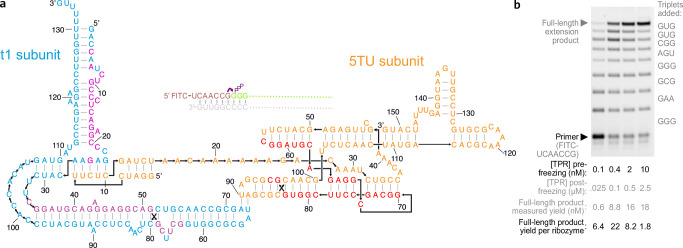
Features and activity of the triplet polymerase ribozyme 5TU^+1^. (**a**) A secondary-structure-level representation of the 5TU^+1^ (5TU/t1) triplet polymerase ribozyme (TPR) heterodimer based upon its cryo-EM structural model^21^. The TPR comprises a catalytic 5TU subunit (orange) and an inactive type 1 (t1) subunit (blue), depicted next to a template-substrate complex. Triplet substrate (green) binding to template (light grey) juxtaposes the 3’ hydroxyl of the upstream RNA primer (brown) and the 5’ triphosphate of the triplet (purple). The 5TU subunit catalyses nucleophilic attack of the 3’ hydroxyl on the triphosphate α-phosphate of a correctly-paired triplet to form a new phosphodiester linkage, repeating this process iteratively. Type 1 forms a heterodimer with 5TU, improving 5TU’s interaction with ligation junctions. The γ fragment of 5TU is highlighted in red, and type 1 regions complementary to products in Extended Data Fig. 8 are highlighted in lilac. (**b**) Primer extension by the TPR. Full-length product is generated when all junctions are ligated, and incomplete ligation yields a ladder of intermediate extension products. Here, multiple-turnover catalysis allows the TPR to synthesise complementary strand RNA upon excess primer/template molecules. 40 nM of single-stranded template A^+^ and primer FITCA were mixed with the indicated concentrations of TPR in 1 mM MgCl_2_, 1 mM CHES·KOH pH 9.0, 4 mM KCl, 0.001% Tween-20, and 0.1 µM of each triplet substrate. To initiate extension, the mixture was frozen in dry ice then incubated at −7 °C for 24 h. Formation of a supercooled liquid brine eutectic phase concentrated all solutes by an estimated 250-fold. Primer extension products were resolved by denaturing PAGE, and gel densitometry allowed quantification of extension and calculation of the number of complementary strands synthesised per ribozyme molecule. Considering that each strand is itself the product of eight iterative ligation reactions, at 0.4 nM TPR each TPR molecule must carry out a ligation every few minutes in ice. Source data

We explored RNA replication catalysed by the 5TU/t1 polymerase ribozyme (henceforth triplet polymerase ribozyme / TPR). This is an artificial heterodimeric ribozyme^21^ that has been evolved in vitro to copy RNA template sequences using trinucleotide triphosphates (triplets) as substrates^4^ (Fig. 1b and Extended Data Fig. 1). As shown previously, RNA-templated RNA synthesis by the TPR is preferentially carried out within the eutectic phase of water–ice at −7 °C, helped by the high ionic and RNA substrate concentrations and reduced water activity therein^22^. Under these conditions, triplet substrates display remarkable properties such as cooperative invasion of template RNA secondary structures, enabling copying of even highly structured RNA templates by the TPR^4^.

We initially hypothesized that this ability of triplets to invade and unravel template secondary structures might be leveraged to invade and replicate otherwise inert RNA duplexes. To test this we assembled a model 30-nt GC-rich RNA duplex A^D^ (Fig. 1b; predicted *T*_m_ = 99 °C)^8^ and incubated it together with its constitutive triplet substrates and TPR. However, although the TPR readily synthesized full-length products on the duplex’s individual RNA (+) and (−) strands (A^+^ and A^−^), the A^D^ RNA duplex itself remained inert (Fig. 1c), indicating that RNA duplex dissociation might be required.

**Extended Data Fig. 2:**
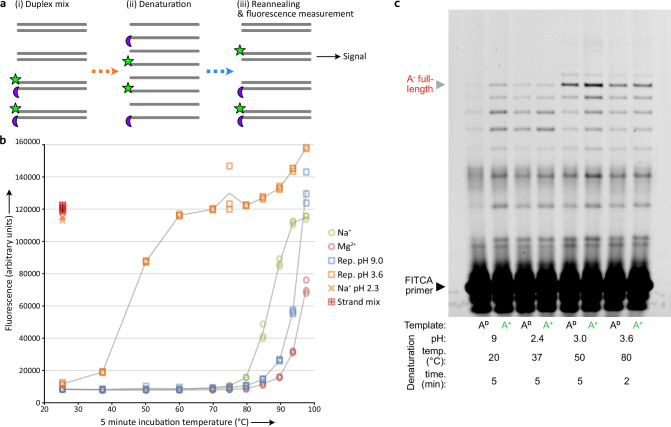
Assaying strand separation under different pH and temperature regimes via fluorescence/quench assay and ribozyme-catalysed duplex replication. (**a**) Fluorescence/quench assay design. (i) The 27-nt RNA duplex Rγ1^D^ (70% GC composition) is mixed with an equal amount of duplex FQγ1^D^, whose component strands are identical but for a 5’ fluorescein group (green) on one and a 3’ Black Hole Quencher group (purple) on the other strand. (ii) Upon subjecting the duplex mix to denaturing conditions, the strands separate from their starting partners. (iii) Returning to native conditions (pH 8, room temperature) leads to reannealing and reassortment of strands. Some fluorophore-labelled strands are now hybridised to unlabelled complements, yielding a fluorescent signal in proportion to the extent of duplex dissociation. This assay decouples the conditions required for measurement from those needed for strand separation, and thus measures the history of denaturation in a sample, not its current state. (**b**) Strand separation of GC-rich RNA duplexes by heating. 1 pmol each of Rγ1^D^ and FQγ1^D^ were mixed in 50 µl of 0.02% Tween-20 and the following buffer compositions: 0.1 M NaCl, 1 mM Tris·HCl pH 7.4 (Na^+^); 0.1 M NaCl, 20 mM MgCl_2_, 1 mM tris·HCl pH 7.4 (Mg^2+^); 2.4 mM KCl, 0.4 mM MgCl_2_, 1 mM CHES·KOH pH 9.0 (Rep. pH 9.0); 2.4 mM KCl, 0.4 mM MgCl_2_, 0.25 mM HCl (Rep. pH 3.6); 0.1 M NaCl, 5 mM HCl (Na^+^ pH 2.3). The samples were incubated for five minutes at the indicated temperatures, then cooled on the bench and neutralized with 50 µl 1 M Tris·HCl pH 8, 25 mM EDTA. After sitting overnight at room temperature, sample fluorescence was measured and plotted against incubation temperature. Reactions were set up in triplicate. A positive control (Strand mix) comprising a mix of 1 pmol of both (+) with 1 pmol of both (-) strands of the duplexes in ‘Na+’ buffer conditions was also prepared. Very high temperatures are needed to dissociate the duplex under neutral or slightly basic conditions, but at an acidic pH of 3.6 moderate heating is sufficient to denature the RNA duplex. (**c**) Combinations of temperature and pH capable of denaturing the RNA duplex A^D^ prior to TPR-catalysed primer extension. Extensions were carried out as in Fig. 2c with different denaturing conditions followed by a single cycle of extension on single-stranded A^+^ or duplex A^D^; matched full-length efficiencies indicate strand separation during denaturation, and maintenance of this state into polymerisation. Source data

Using a fluorescence-quench assay, we found that temperatures over 90 °C were required to dissociate a mixed-sequence 30-nt RNA duplex (Rγ1^D^) into constituent strands (Extended Data Fig. 2). In the presence of millimolar concentrations of Mg^2+^ ions (needed for ribozyme catalysis), even higher temperatures approaching the boiling point of water were required. Exposure to such temperatures in the presence of Mg^2+^ would cause rapid fragmentation of longer RNA strands, including the TPR catalyst. We thus explored alternative approaches to destabilize the RNA duplexes.

Mildly acidic pH had previously been shown to destabilize short RNA duplexes and is not destructive to RNA^10^, which—unlike DNA—does not suffer depurination at low pH. However, we found that (at ambient temperatures) A^D^ duplex dissociation of >50% still required low pH (pH ≤ 3, or pH ≤ 2.5 with added 20 mM MgCl_2_; Supplementary Fig. 1). Although incompatible with polymerase ribozyme activity (pH_opt_ ≈ 8.8), we tested if acid-induced RNA duplex dissociation could be leveraged for copying of the A^D^ duplex after neutralization. We therefore performed (1) acid denaturation of the A^D^ duplex, followed by (2) neutralization and concurrent addition of TPR, primer and triplet substrates, and (3) freezing and TPR-catalysed polymerization at −7 °C. This yielded full-length synthesis of both A^+^ and A^−^ constituent strands starting from A^D^ duplex template (at 30–40% of the yields compared to individual single-stranded A^+^ or A^−^ strands as templates; Fig. 1c).

We next investigated the kinetics of RNA duplex reannealing and replication in such reactions, by progressively delaying ribozyme addition after the neutralization step. To our surprise, extension on a pre-denatured duplex was maintained even when ribozyme was added up to six days post-neutralization (Fig. 1d). However, if triplet addition was also delayed, subsequent extension was rapidly reduced (*k*_obs_ ~ 0.7 µM^−1^ min^−1^; Fig. 1d), presumably due to rapid reannealing of the two dissociated A^+^ and A^−^ RNA strands.

**Extended Data Fig. 3:**
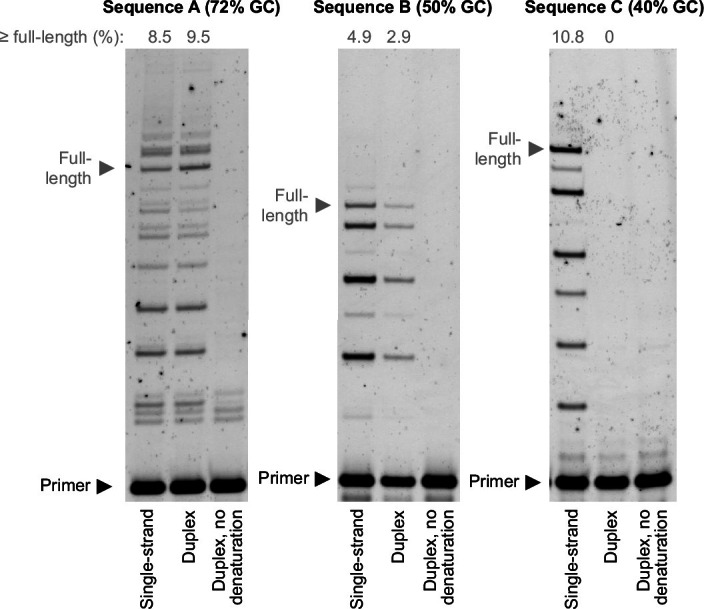
Influence of substrate GC-content upon inhibition of reannealing. One cycle of replication in replication buffer, carried out as in Fig. 2c, but on RNA single strands or duplexes of different sequence compositions, supplying their constitutive triplet substrates and primers from the start (Sequence A = A^+^/A^D^ template (>50% GC), FITCA & Cy5A primers, constitutive triplets as in Fig. 1b; Sequence B = B^+^/B^D^ template (50% GC), FITCB & Cy5B primers, constitutive triplets; Sequence C = C^+^/C^D^ template (40% GC), FITCC & Cy5C primers, constitutive triplets). Although the TPR can synthesise full-length extension products of FITC-labelled primers on all three single-stranded templates, it could only do so on double-stranded duplexes of 50% GC composition or higher. The capacity of low concentrations of GC-rich triplets – but not high concentrations of AU-rich triplets – to support duplex copying likely reflects the extreme cooperativity of strand coating (via Watson-Crick pairing and base stacking) needed to maintain a kinetic barrier to reannealing. Source data

We hypothesized that dissociated RNA strands become kinetically trapped in a single-stranded state when partially (or fully) hybridized to complementary triplets, which robustly attenuate strand reannealing, providing a time window for RNA polymerization (Fig. 1e). Consistent with this hypothesis, even partial triplet occupancy was sufficient to attenuate reannealing: the addition of triplets complementary to just one of the strands (A^+^ or A^−^) slowed reannealing by ~200-fold (Fig. 1d), and addition of complementary triplets to both strands effectively stopped the second-order reannealing process and maintained strands in a dissociated state for more than 300 h. A further prediction of this scenario is that such ‘substrate-assisted replication’ would be contingent on the nature of the triplet substrates. Indeed, we found that AU-rich triplets on 40% GC templates are unable to compete effectively with strand reannealing, and templates of balanced (50% GC) composition lead to a mix of reannealing and extension (Extended Data Fig. 3). In contrast, on GC-rich templates (such as A^+^, A^−^), inhibition of intermolecular annealing is effective at low (almost stoichiometric) triplet concentrations (~2.5 µM per triplet; Supplementary Fig. 2)—even below the triplet concentrations needed for cooperative invasion of intramolecular template RNA secondary structures such as hairpins (>12 µM per triplet)^4^. This effect is specific to the triplet substrates; an RNA polymerase ribozyme even with high concentrations of mononucleoside triphosphate (NTP) substrates^23^ (5 mM of each NTP in the eutectic phase) exhibited only negligible extension on a pre-denatured duplex (Supplementary Fig. 3).

**Extended Data Fig. 4:**
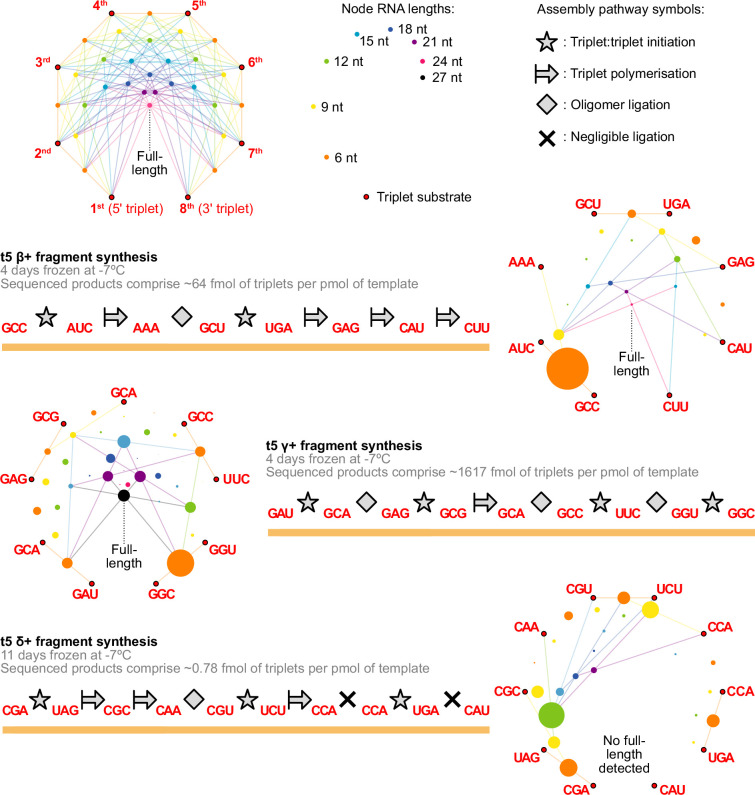
Primer-free synthesis pathways of triplet polymerase ribozyme fragments. Above, Potential synthesis pathways to a 24 nt RNA from triplet substrates. Each node represents a synthesis intermediate, of increasing length towards the centre of the map (full length product). The intermediate at each node can be synthesized from the intermediates or triplets at each end of a line passing through the node. Larger intermediates can potentially be generated via multiple routes. Below, Maps of products detected during primer-less synthesis of three fragments (β+ (top), γ+ (middle) and δ+ (bottom)) of the t5 ribozyme^4^, and their inferred synthesis pathways. The maps show the distribution of polymerised triplets: the area of each node is proportional to the abundance of that intermediate (from sequencing scheme in Supplementary Fig. 4) multiplied by its length. Node sizes are uniformly scaled in each map such that the sum of intermediate node areas is constant; the triplet substrate nodes (not sequenced) are sized arbitrarily. Putative synthesis pathways are marked on each map and displayed alongside in a 5’ to 3’ direction with intervening symbols indicating how triplets are joined together into RNA products – indicating sites of initiation (triplet:triplet ligation), polymerisation (triplet addition to the 3’ of an oligonucleotide) or instances of ligation (of oligomer substrates larger than triplets). The β fragment exhibits two sites of initiation, followed by polymerisation from each with their concomitant ligation on the path to the full-length fragment. The γ fragment synthesis is characterized by multiple sites of initiation and independent ligation of the resulting oligomers, and proceeded effectively, perhaps reflecting the availability of multiple initiation sites. The δ primerless synthesis was inefficient, with negligible ligation at two junctions, and as a result no full-length product was detected. The observed products suggested a pattern of two initiation sites, followed by polymerisation and ligation. The triplet:triplet ligation observed across the three templates is evidence of widespread triplet substrate coating of single-stranded templates. Source data

Our data are consistent with a dual role of triplets, both as RNA chaperones keeping complementary strands from reannealing—progressively ‘coating’ RNA strands via specific hybridization and cooperative triplet–triplet stacking interactions—and as substrates for RNA replication. These ratchet-like processes progressively stabilize the template dissociated state until a full complementary strand is synthesized. This model is supported (and potentially enhanced) by the capacity of the TPR to initiate templated ‘primer-free’ RNA synthesis internally from adjacent triplets at multiple sites along the template (Extended Data Fig. 4 and Supplementary Fig. 4). By blocking strand reannealing and creating a long-lived substrate:template complex, triplets decouple RNA polymerization from both the kinetics and thermodynamics of RNA duplex dissociation and reannealing (Fig. 1e).

### Iterative RNA replication cycles

**Fig. 2:**
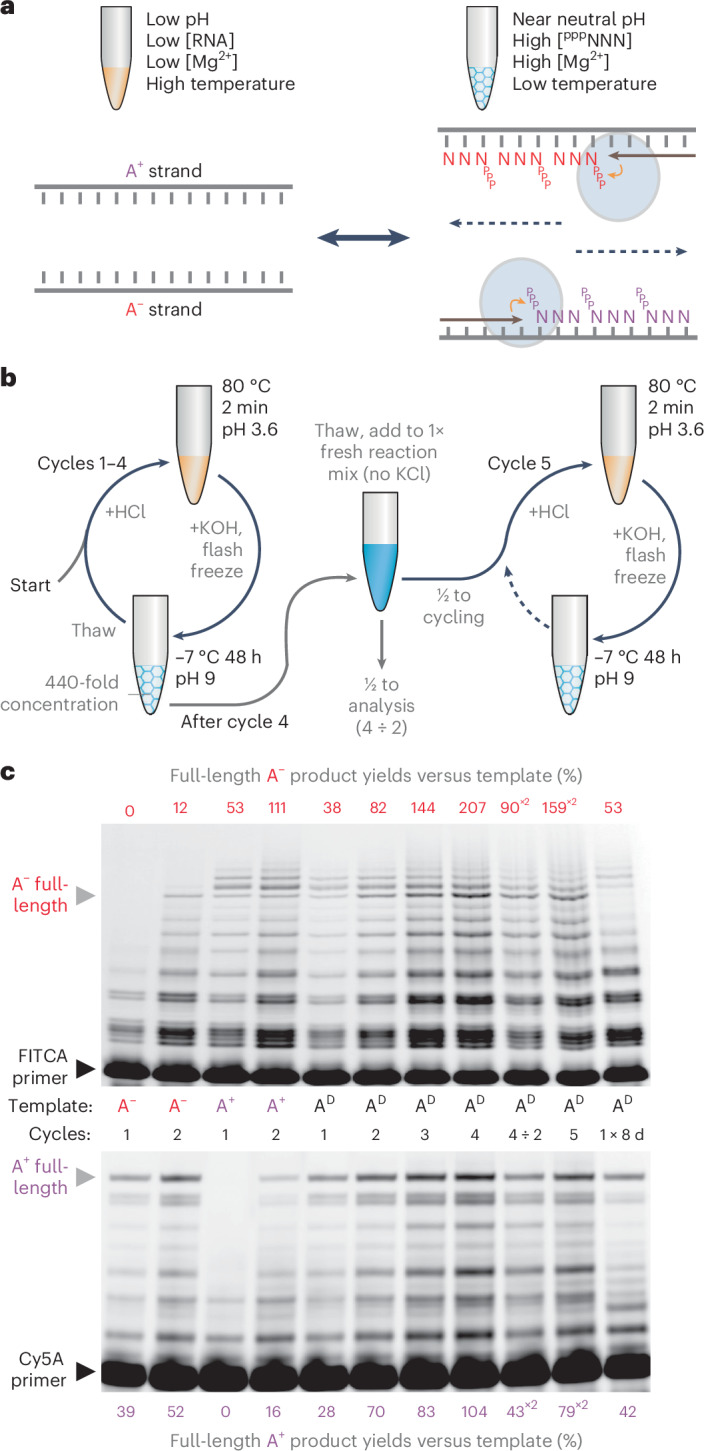
Freeze–thaw/pH cycling allows iterative cycles of RNA replication. **a**, Schematic of conflicting conditions required for RNA strand separation (left) and triplet polymerization (right). **b**, Physicochemical cycling workflow that integrates strand separation and polymerization conditions. pH switching results in a build-up of KCl, and serial dilution allows continued cycling by resetting KCl concentrations and restoring ribozyme and triplet substrate levels. **c**, Iterative replication of the model RNA duplex A^D^ and its constituent strands in replication buffer (4 nM template, substrates and primers from Fig. 1b). Also shown (for comparison) are a single-cycle eight-day polymerization reaction (1 × 8 days), and four-cycle reactions undergoing twofold dilution (4 ÷ 2) followed by an extra cycle (5). Full-length primer extension yields are expressed as percentages relative to the starting template. To compare efficiencies in the diluted 4 ÷ 2 and five cycle reactions, their yields should be doubled (×2). Source data

Having discovered an effective strategy to overcome the strand separation problem, we sought to integrate triplet-assisted strand separation and triplet-based RNA synthesis into a full RNA replication cycle. However, the preferred conditions required for strand separation and RNA synthesis are diametrically opposed. Effective RNA duplex denaturation requires low pH and elevated temperatures to weaken base-pairing interactions, together with low Mg^2+^ concentrations ([Mg^2+^]) to reduce duplex stability and minimize RNA hydrolysis, and low RNA strand concentrations to slow down reannealing. In contrast, optimal RNA synthesis requires a mildly basic pH, high [Mg^2+^], high ribozyme and triplet substrate concentrations ([RNA]) and ambient to low temperatures. Furthermore, incubation of RNA at acidic pH in the presence of the high [Mg^2+^] needed for optimal ribozyme activity (>60 mM) leads to precipitation and inactivation of long RNAs—an effect exploited in the trichloroacetic acid precipitation of nucleic acids. A replication cycle would therefore require opposing shifts in pH, temperature and solute concentrations ([Mg^2+^], [RNA]) (Fig. 2a).

To reconcile these conflicting requirements, we first defined milder, more dilute denaturing conditions (pH 3.6, 80 °C, low [Mg^2+^] and [RNA]) that efficiently separate even GC-rich RNA duplexes (Extended Data Fig. 2) while avoiding RNA degradation. To access synthesis conditions after neutralization, we noted that freezing can drive a more than 200-fold concentration of solutes and reduction of water activity within the eutectic brine phase while supporting polymerase ribozyme activity^22^. We thereby established a coupled pH/freeze–thaw replication cycling regime (Fig. 2b,c), which shifts between denaturing conditions that efficiently dissociate RNA duplexes and extension conditions that yield near-full TPR activity (Supplementary Fig. 5).

When applied to individual A^−^ or A^+^ template strands, a single cycle achieved per-strand yields of 39% or 53%, and a second cycle nearly doubled these yields (52% or 111%; Fig. 2c). This second cycle was also accompanied by full-length extension of primers associated with the starting strands (yields of 12% (A^−^) and 16% (A^+^)). This indicates that in cycle 2, the product strands of cycle 1 are used as templates, providing a foundation for exponential RNA amplification.

**Extended Data Fig. 5:**
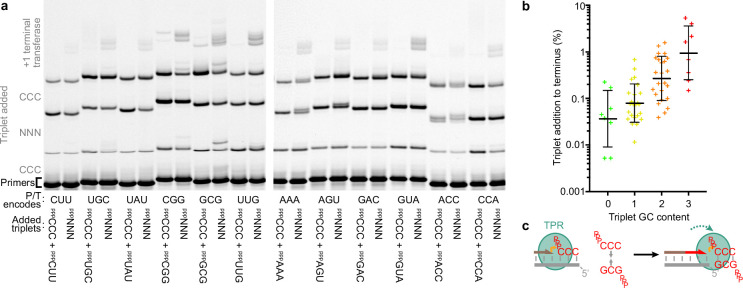
Terminal transferase activity by a triplet polymerase ribozyme. (**a**) Terminal transferase activity by the t5^+1^ TPR^4^ (the precursor of the 5TU^+1^ TPR^21^) when extending 12 different primer/template (P/T) pairs. These template incorporation of ^ppp^CCC preceding and following one of 12 different triplets (indicated). As substrates, either all 64 triplets (^ppp^NNN) or only the specific templated triplet and ^ppp^CCC were provided. With ^ppp^NNN, on average 29% of the full-length products were extended by a fourth triplet in a non-templated manner. Extensions were carried out as previously described (ref. ^4^, ‘Fidelity assay’ section of Methods). (**b**) RNA products in a mixture of the reactions that used ^ppp^NNN in (**a**) were sequenced (see ref. ^4^). The probabilities of each of the 64 triplets being added (non-templated) to a full-length product were plotted by triplet GC content. GC-rich triplets were better substrates for terminal transfer (means ± s.d. after logarithmic conversion, n = 8 (0 & 3 GCs) or 24 (1 & 2 GCs)); from ^ppp^NNN, 19% of all non-templated triplets added were ^ppp^CCC. (**c**) Despite the uniform presence of the best terminal transferase substrate ^ppp^CCC in (**a**), terminal transferase activity was only notable when an additional G-rich triplet was present. We propose that duplex growth proceeds through blunt-ended addition of short RNA duplexes formed by pre-associated GC-rich Watson:Crick-paired triplet dimers, whose independent existence in solution was previously inferred by the influence of complementary triplets upon TPR misincorporation frequencies^4^. Source data

To test this, we initiated repeated cycles of pH/freeze–thaw replication starting from the duplex RNA A^D^ and observed simultaneous production of both full-length A^−^ and A^+^ synthesis products. After four cycles, the product yields reached approximately two A^−^ strands (207%) and one A^+^ strand (104%) per starting A^D^ duplex (Fig. 2c), while only ~0.5 strands of each was produced per duplex in a single long cycle with equivalent total reaction time. During this long incubation, the products were also elongated beyond full length, indicative of a TPR-catalysed terminal transferase activity, probably through blunt-ended ligation of the GC-rich triplet:triplet dimers (Extended Data Fig. 5) that were previously inferred to form in ice^4^.

**Extended Data Fig. 6:**
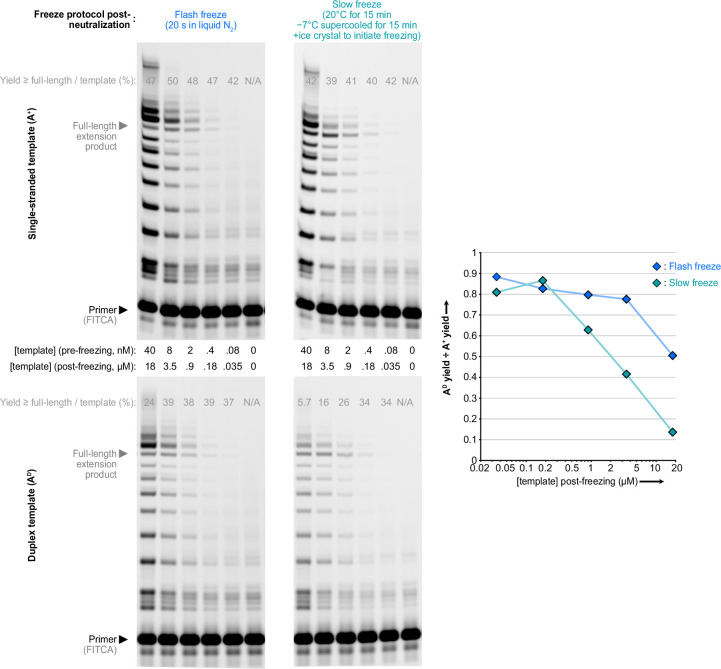
Influence of RNA duplex concentrations and the freeze/thaw/pH cycling protocol upon replication efficiency. Flash-freezing maximises replication efficiency at high RNA duplex concentrations. A single cycle of TPR-catalysed replication (as in Fig. 2c, with 2.4 mM KCl, −7 °C for 40 h) was applied to different concentrations of single-stranded (ss) RNA (top) or double-stranded (ds) duplex RNA (bottom) templates, and full-length yields per template were calculated by gel densitometry. When using ss templates, the yield was broadly constant. However, when using high concentrations of ds templates the yield dropped in a manner dependent upon the protocol used to freeze the reaction after acidification, heating and neutralisation (yield ratios plotted on the right). A flash-freeze in liquid N_2_ over 20 s (blue) maintained yield at a higher concentration of duplex than a slow freeze over half an hour (teal). Reannealing before freezing is more prevalent at higher duplex concentrations and longer delays between neutralisation and eutectic phase formation. In effect, these parameters govern the relative weights of the ‘reannealing’ and ‘strand coating’ arrows in Fig. 1e and thus the productivity of the replication cycling. Efficient duplex use (~80% vs. ssRNA) was nonetheless observed at eutectic phase concentrations of 3.5 µM (after flash-freeze) or 0.8 µM (after slow freeze). This concentration may be considered a critical parameter of any cycling protocol, as it governs the maximum concentration of each RNA species that can be generated via exponential replication. Reassuringly, ribozymes are operational at these concentrations, including the TPR itself (Extended Data Fig. 1), suggesting that sufficient catalysts could accumulate via this cycling protocol to drive RNA catalytic processes including self-replication. Source data

The timescale and steepness of the concentration and temperature and pH gradients during these shifts impact the synthetic yields of RNA replication. For example, flash-freezing gives the highest RNA yields as it minimizes the amount of strand reannealing between the neutralization step and freezing (when triplets become sufficiently concentrated to attenuate reannealing). Nevertheless, even slow cooling supports efficient RNA replication, but reannealing begins at lower RNA duplex concentrations in the eutectic phase (4 µM and 1 µM, respectively; Extended Data Fig. 6). Effective replication cycles can also proceed across a range of temperature and pH conditions, using smaller temperature shifts (to 50 °C or even 37 °C), but require a lower pH (Extended Data Fig. 2).

### Open-ended RNA amplification

**Extended Data Fig. 7:**
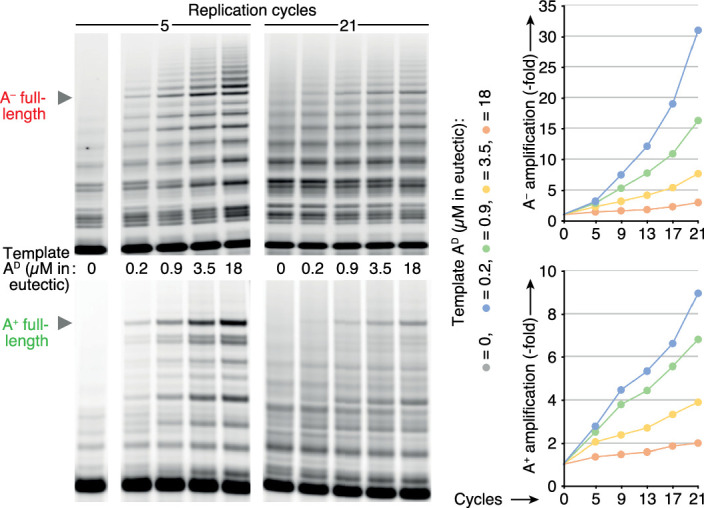
Replication efficiency of template A upon iterative replication cycling. Left panels, Replication reactions were set up as in Fig. 2c with varying starting A^D^ duplex concentrations and continued over 21 cycles (including 5 serial dilutions as shown in Fig. 2b). Right panels, Calculated strand amplification in these reactions. After x cycles, the concentration of primer reaching full-length (above that in the no template control) (= [≥FL(x)]) was measured by densitometry and normalized to the expected concentration of starting A^D^ remaining after serial dilution: Amplification after x cycles = 1 + 2^((x-1)/4)*([ ≥ FL(x)] ÷ starting [A^D^]). Source data

In our pH/freeze–thaw cycling scheme, HCl and KOH addition drives the pH changes. This results in a buildup of K^+^ (and Cl^−^) ions, eventually inhibiting polymerization (Supplementary Fig. 6). We therefore imposed a serial, twofold dilution regime (into fresh reaction mix containing ribozyme and substrates, but lacking template and KCl) every fourth cycle to reset the ionic strength (Fig. 2b). Continuing A^D^ replication under this regime for 21 cycles yielded diminishing returns in A^+^ and A^−^ yields, probably due to TPR/product degradation and the low yield of A^+^ strands (Extended Data Fig. 7). Despite this, we observed overall strand amplifications of ninefold (A^+^) and 31-fold (A^−^) after accounting for the iterative reaction dilution.

**Fig. 3:**
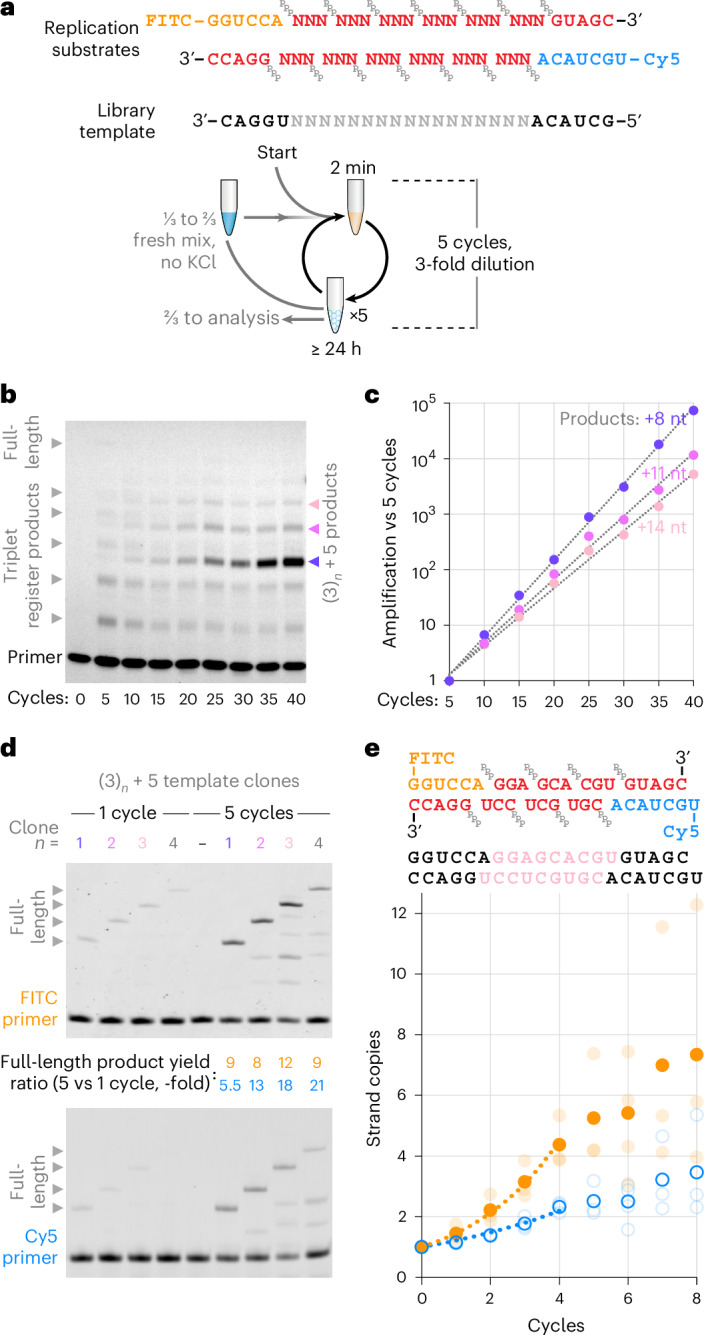
Open-ended exponential amplification of RNA. **a**, Design of replication substrates and scheme for iterative replication of an N_17_ RNA random-sequence library. To start, library template LibN_17_ was mixed at 8 nM in replication buffer (including 0.9 mM KCl and 20 nM TPR) together with 20 nM each of the indicated primers (FITCrep, Cy5rep, ^ppp^GUAGC, ^ppp^GGACC) and 12 nM each of all 64 triplets (^ppp^NNN). **b**, FITCrep extension products from this reaction analysed at each five-cycle interval before threefold serial dilution. **c**, Quantification of overall amplification of ‘(3)_*n*_ + 5’-register products in **b**, calculated as the fold increase in band intensity versus five cycles, multiplied by reaction dilution versus five cycles. Exponential fits yielded the per-cycle amplification efficiencies described in the text. **d**, Reactions set-up as in **a**, but seeded with 0.8 nM of both strands of one emergent RNA duplex sequence from **a**–**c** (Rep(1–4)^+^ with Rep(1–4)^−^, detected in the sequencing of each product population of the final reactions in **b**) as a template, using their constituent triplets as substrates (middle lane: triplets from all four sequences but no template). **e**, Cycling as in **a** of 0.8 nM of both strands of one clone (shown here beneath its substrates) without dilution. Average strand copy numbers (filled orange circles, FITC strand; open blue circles, Cy5 strand; comprising full-length and starting template) are shown for three independent reactions per cycle (transparent circles). Dotted lines are exponential curves fitted up to four cycles (*x*): FITC strand = e^0.38*x*^, *R*^2^ = 0.997; Cy5 strand = e^0.20*x*^, *R*^2^ = 0.984. Source data

To better assess the potential of this cycling protocol in an open-ended context, we initiated RNA replication from a random-sequence template (N_17_) flanked by defined primer binding sites (Fig. 3a) and provided all 64 triplet substrates (^ppp^NNN). We hypothesized that if RNA replication were sustained, we would initially observe drift, then persistence and amplification of those RNA sequences that can both be synthesized and copied efficiently by the TPR. In early cycles, we observed a ladder of triplet-register extension products corresponding to the expected library size (6 × ^ppp^NNN triplet incorporations + a terminal pentamer (^ppp^GUAGC) adding a primer binding site; Fig. 3b). As cycling progressed (up to 40 cycles), the full-length product progressively faded, and a new, second series of shorter products emerged, identifiable by their altered register of migration. These persisted and increased up to 30-fold in abundance. Accounting for the >2,000-fold effective dilution over the course of the experiment, these emergent +8-, +11- and +14-nt product classes have undergone an up to 60,000-fold amplification, with apparent exponents of 1.37-, 1.3- and 1.27-fold per cycle, respectively (Fig. 3c). They probably arose from early incorporation of the terminal pentanucleotide substrate on some library templates, providing a reciprocal primer binding site, and supporting higher yielding synthesis of the shorter amplicons—a well-known phenomenon in polymerase chain reaction (PCR)-style primed amplifications^24^.

To better understand the sequences that are synthesized and copied efficiently in our replication reactions, we sequenced +8-, +11- and +14-nt product pools (Supplementary Table 1). Although no clear sequence consensus was found to dominate the pools, many sequences comprise triplets exhibiting a (G/C, G/C, N) compositional pattern (compatible with reciprocal replication due to the register shift; Fig. 3a). We tested replication of a common emergent sequence of each length. These showed escalating synthesis of both product strands during replication cycling (Fig. 3d). The (+) and (−) strands of the +14-nt sequence exhibited a four-cycle exponential growth phase (1.2- and 1.38-fold per cycle) before plateauing at 3.5- and 7-fold amplification from the starting duplex (Fig. 3e). This confirms that pH/freeze–thaw cycling lets the TPR exponentially replicate RNA sequences, with open-ended cycling driving the evolution of replicable RNAs from libraries.

### Fragmentary self-replication

**Fig. 4:**
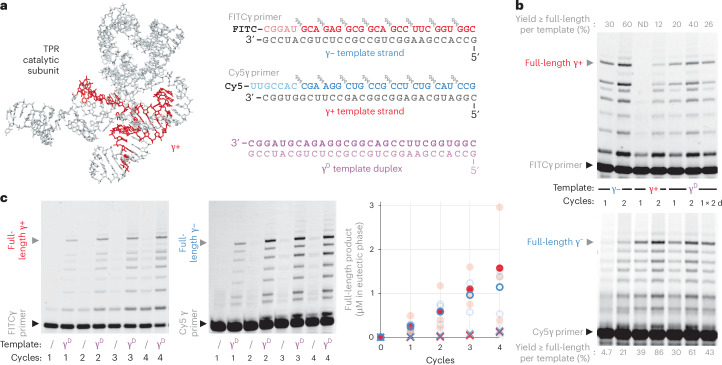
Ribozyme-catalysed replication of a fragment of itself. **a**, 3D structural model^21^ (left) of the TPR catalytic subunit with the γ^+^ segment highlighted in red. Right, sequences of the γ^D^ duplex and its constituent strands γ^+^ and γ^−^, shown with primer and triplet substrates. **b**, γ^+^ and γ^−^ synthesis during replication of single strands and duplex. The triplets (0.2 µM each) and primers (0.1 µM each) shown in **a** were polymerized by the TPR (20 nM) on the indicated templates (2 nM each) in replication buffer across one or two denaturing acid cycles (polymerization: 24 h at −7 °C). Per-template yields of primer reaching full length are shown; two cycles yielded more product than a single cycle with equivalent total incubation time in ice (1 × 2 days). **c**, Iterative replication cycling of reactions (set-up as in **b**, with 0.6 mM starting KCl) with no template or 4 nM γ^D^ duplex (concentrated to ~1.8 µM in the eutectic phase). The eutectic phase concentrations of products reaching full-length were inferred from gel densitometry. Averages are shown of three independent reactions (transparent data points) set up for each cycle number. γ^+^ from γ^D^ template, filled red circles; γ^−^ from γ^D^ template, open blue circles; γ^+^ or γ^−^ products from no γ^D^ template, red/blue dashed crosses. Source data

**Extended Data Fig. 8:**
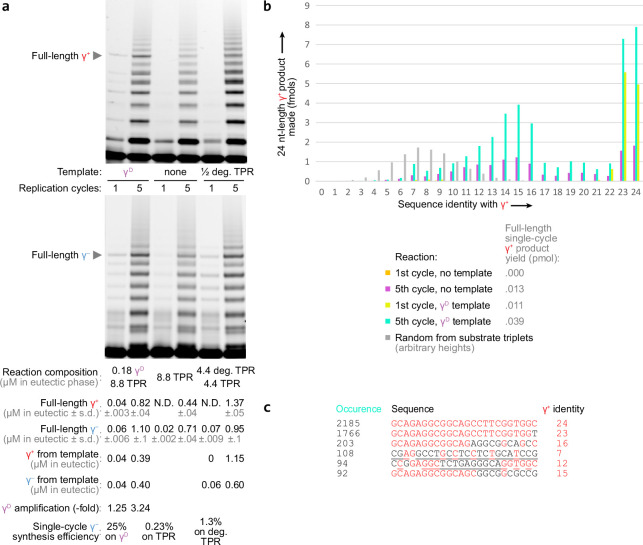
γ-fragment replication reactions, sequencing and analysis. (**a**) Iterative replication of exogenous or endogenous γ fragment by the TPR. Replication reactions were set up as in Fig. 4b (with 0.6 mM KCl), and either seeded with γ^D^, no exogeneous template (TPR) or partly degraded TPR (by prior heating with Mg^2+^ = ‘deg. TPR’). Post-freezing (eutectic phase) concentrations of template, TPR and products are shown. Concentrations of γ^+^ (red) and γ^–^ (blue) products reaching full-length were inferred from gel densitometry of triplicate reactions. Concentrations of product derived from replication of the template seed were calculated by proportional subtraction of products derived from intact ribozyme in unseeded reactions. This showed low single-cycle γ^–^ synthesis efficiency using TPR as a template, but higher efficiency upon partial TPR degradation. Replication efficiency from γ^D^ is higher than in Fig. 4c, likely due to the 10-fold lower starting concentration of γ^D^ duplex used here. (**b**) Yields of full-length fragment replication products classed by γ-fragment homology. The sequence product distribution in Supplementary Fig. 7c for each sample was scaled by the modelled yield of all γ^+^ products in the corresponding cycle (Supplementary Fig. 7d). (**c**) The six most common individual sequences in the 5-cycle γ^D^ replication sample (occurrence shown out of 10,880 total sequences). Interestingly, the two of these sequences with very low γ^+^ identity show complementarity (underlined) to parts of the type 1 ribozyme (see Extended Data Fig. 1), a further instance of TPR acting as a template. Source data

Having previously shown that the TPR can synthesize segments of both its (+) and (−) strands^4^, we asked if replication could be extended to parts of the TPR’s own sequence. A potential pitfall in this context is the potential for invasion and self-inhibition of the (+)-strand TPR by complementary (−)-strand TPR sequence segments following denaturation during cycling. We tested this using a 29-nt fragment of the ribozyme catalytic core previously designated the γ fragment^4^ (Fig. 4a). After one replication cycle on the γ^D^ duplex, we obtained both full-length γ^+^ and γ^−^ product strands, and after two cycles we saw evidence of replication of both individual γ^+^/γ^−^ and duplex γ^D^ templates (Fig. 4b), with the products being used again as templates in a second cycle, as observed for A^D^ (Fig. 2c). We observed no self-inhibition, presumably because the TPR tertiary structure either does not unfold under our denaturation conditions, or refolding is faster than complementary strand invasion. During iterative cycles of γ^D^ partial self-replication, full-length product levels increased steeply in early cycles, reaching micromolar concentrations in the eutectic phase (Fig. 4c) and exhibiting up to 1.26-fold amplification per cycle (Extended Data Fig. 8). The TPR itself is active at micromolar concentrations (Extended Data Fig. 1), indicating that our cycling protocol could support production of a ribozyme at the concentrations needed for activity.

Unexpectedly, we also observed the synthesis of γ^+^ and γ^−^ product strands in an unseeded (no template) negative control reaction (Fig. 4c). Even after a single cycle, both unseeded or exclusively γ^−^-seeded reactions yielded γ^−^ products (whereas γ^+^-seeded reactions showed no γ^+^ synthesis; Fig. 4b). We hypothesized that the γ^+^ segment within the TPR itself might be acting as a template, but at low efficiency compared to exogenously added γ^+^ template. Indeed, after 50% degradation of the TPR (by heating at pH 9.0 with Mg^2+^ before replication cycling), we observed a greater buildup of γ^+^ and γ^−^ synthesis products after five cycles, and more γ^−^ extension products after a single cycle (Extended Data Fig. 8). This suggests that degraded TPR fragments can act as replication templates, boosting product yields despite the lower amount of active TPR catalyst available for RNA replication.

Sequencing the products of γ^D^-seeded reactions confirmed the replication of accurate γ^+^ sequences alongside a number of partially homologous sequences probably derived from incomplete extension products undergoing recombination, and even some unanticipated products complementary to the TPR t1 subunit (Extended Data Fig. 8). Weighting the sequence data by reaction yield provided estimates of the production rates of different sequence classes, establishing that the production rate of accurate γ^+^ products increased almost 50% from cycles 1–5 of γ^D^-seeded replication, with a replication yield of 130% accurate γ^+^ strands after five cycles (Supplementary Fig. 7). Lower levels of accurate γ^+^ sequences also emerged in the unseeded reaction. These results show that (1) a ribozyme can exponentially replicate part of itself from short building blocks, (2) this ‘fragmentary’ self-replication can occur in a ‘one-pot’ cycled reaction and (3) ribozymes can initiate replication on themselves, even in the absence of a seed template.

### Emergence and replication of RNA sequence pools

In all the RNA replication reactions described so far (Figs. 2–4), we provided sequence-specific RNA primers for the replication of either defined or random-sequence templates. However, the availability of specific RNA primers is unlikely in a prebiotic context^1^. We wondered if the capacity of the TPR to initiate replication from template-bound RNA triplets (Extended Data Fig. 4) could support a more prebiotically plausible primer-independent model of RNA-catalysed RNA replication.

**Fig. 5:**
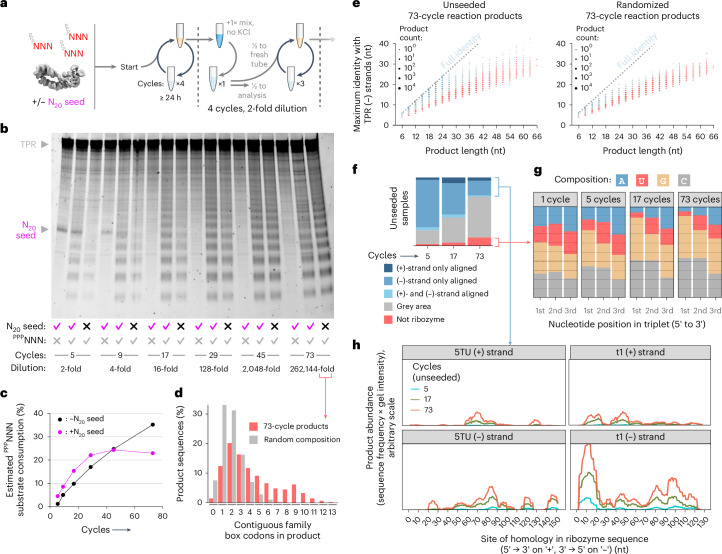
Emergence and amplification of RNA pools during primer-free triplet-based replication cycling. **a**, All 64 RNA triplets (^ppp^NNN, 0.1 µM each) ± 20 nM N_20_ random-sequence RNA template seed were subjected to iterative cycles of replication and dilution in replication buffer (with 20 nM TPR and 1.8 mM KCl, but no primers). **b**, RNAs present at different replication cycles (up to 73) were visualized with an intercalating dye. Both the intensity and length of the RNA products increased during cycling, despite serial dilution. **c**, Estimated conversion of the total triplet substrate pool into the RNA products. The amount of triplets needed to constitute calculated RNA product yields was expressed as a percentage of the replenished triplet substrates (Extended Data Fig. 10). **d**, In silico ‘translation’ using a reduced codon set applied to the sequenced, unseeded 73-cycle synthesis products (red), compared to a simulated pool of random sequences with matching lengths but unbiased codon composition (grey). For each sequence, the longest stretch of family box codons is counted to show the maximum potential length of any encoded peptide using only a primordial genetic code. **e**, A population of sequenced products from the unseeded 73-cycle reaction (left) shows high ribozyme sequence complementarity, absent in a simulated pool of randomized RNAs of identical composition (right). Data are coloured by the classification in **f** (Extended Data Fig. 9 shows the criteria used); sequences with homology to the ribozyme (+) strand are plotted separately (Supplementary Fig. 8). **f**, Changes in proportions of sequence classes in 9–27-nt products from unseeded reactions during amplification. **g**, As cycling progresses, the G–C base composition of sequences classed as having no ribozyme homology increases (data from N_20_-seeded reactions shown). **h**, Mapping of ribozyme-homologous parts of 9–27-nt products from unseeded amplification reactions to the (+) and (−) strands of the TPR subunits 5TU and t1. Peak heights reflect the fraction of products homologous to that site, scaled by the product intensity in the corresponding gel lane in **b**. Products with homology to multiple locations on one or both strands were randomly assigned to one. Note the prior emergence and buildup of (−)-strand TPR homology products, followed by (+)-strand products (templated from (−)-strand products). Source data

To investigate such a replication scenario, where the TPR is free to explore RNA sequence space in an unguided manner, we initiated open-ended RNA replication cycles, without primers, but providing all 64 triplet substrates (^ppp^NNN) and an N_20_ random-sequence RNA seed pool (Fig. 5a). After five cycles, a ladder of triplet-register RNA products was detected (Fig. 5b), indicating primer-free RNA synthesis from the N_20_ template seed. To our surprise, a similar (if fainter) ladder was also seen in the unseeded (no template) control, implying emergence of products even in the absence of a seed template.

Following continued cycling (with serial dilution), the levels of synthesized RNA in both seeded and unseeded reaction pools grew, and product lengths increased. After 73 cycles (and over 2.5 × 10^5^-fold dilution of the initial reaction), a robust ladder of products was present in both seeded and unseeded reactions (Fig. 5b), consuming a substantial fraction of the ^ppp^NNN triplet substrates supplied upon each dilution step (Fig. 5c). Although the seeded reaction initially maintained a higher level of RNA products (over a 128-fold dilution), RNA product levels in the unseeded reaction eventually matched the seeded reaction.

**Extended Data Fig. 9:**
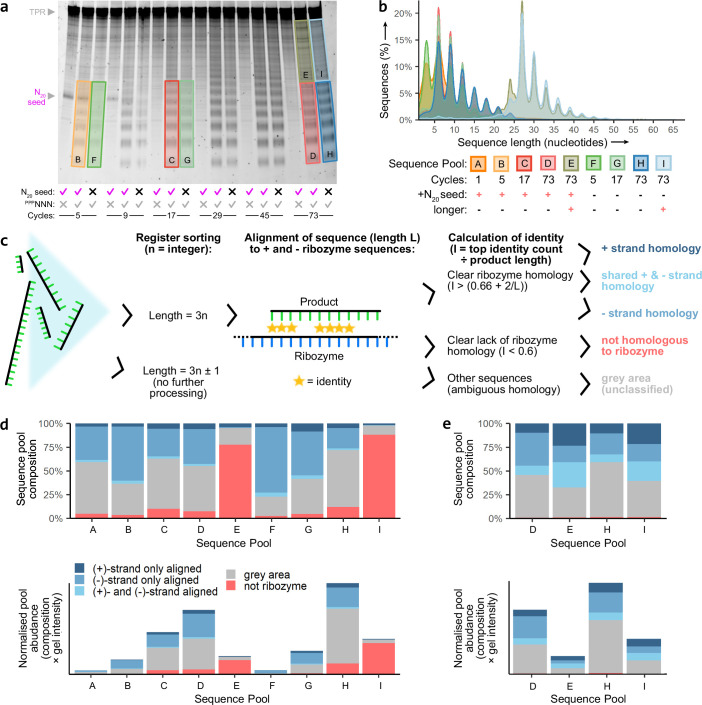
Sequencing and classification of amplification products made from ^ppp^NNN substrates. (**a**) Amplification products derived from indicated regions of the gel in Fig. 5b were sequenced and analysed as product pools B-I, alongside products from the corresponding 9–27 nt region of a separate equivalent 1-cycle seeded amplification (pool A). (**b**) Length distributions observed within the pools of sequenced RNAs. The strong triplet register bias confirms sequencing of ribozyme-synthesised RNAs. (**c**) Workflow for classification of the sequenced products. Only triplet-register products were analysed. Amplification products that exceeded stringent length-dependent sequence identity thresholds at any point when aligned along the (+) strand sequences of either 5TU or t1 TPR subunits (or their (-) strand complements) were classed as possessing ribozyme homology. These were further subdivided by the (+) or (-) strand they were matched to; some showed homology to both (unsurprising in a hairpin-rich RNA with internal complementarity) and were classed separately. Sequences that clearly did not align to the ribozymes or their complements at any point were also classed separately. Sequences between the indicated identity thresholds could not be easily categorised and were excluded from further analysis. (**d**) Levels of ribozyme homology within RNA product pools fractions (top), and corrected for corresponding RNA yields (bottom) based upon intercalator fluorescence in the excised region in part (**a**). A substantial fraction exhibits complementarity to ribozyme. All categories of product increase in absolute abundance over the course of cycling. (**e**) Reclassification of longer amplification products in pools E and I via only the 4^th^–12^th^ nt of each sequence. This showed that the analysis in (**d**) suggesting that most of the > 27 nt sequences exhibited no overall ribozyme homology was partly an artefact of aligning all along these longer sequences; instead, there is a similar level of local ribozyme homology in longer (pools E & I) and shorter (pools D & H) sequences. The lower global ribozyme homology implicates recombination in this discrepancy. Note that the 9 nt length window used here is too short to definitively class sequences as ‘not derived from ribozyme’ using our identity thresholds. Source data

To understand the nature of the emergent RNA sequences, we performed next-generation sequencing of all 5′-triphosphorylated RNAs from both seeded and unseeded reactions (Extended Data Fig. 9). We observed products ranging from 6 to 60 nt in length, with many (25–75%) in both seeded and unseeded reactions showing some complementarity to the TPR ribozyme sequence (Fig. 5e,f and Supplementary Fig. 8). This suggests that, as observed above in the primer-dependent γ^D^ replication (Fig. 4b), the TPR acted both as a polymerase and as a template. The absolute amount of ribozyme-complementary products increased progressively in later cycles (Extended Data Fig. 9), with homologous regions mapping to multiple initiation points along the TPR sequence (Fig. 5h). Although some TPR sequence segments appear to be absent (for example 5TU(−) 30–45, 125–140), products homologous to TPR subunit 5TU or t1 (−) strands build up in the cycling reactions, followed in later cycles by products with homology to the 5TU/t1 (+) strands, suggesting that the emergent (−)-strand segments begin to act as templates, themselves instructing (+)-strand synthesis (Fig. 5h and Supplementary Fig. 8).

A fraction (5–20%) of RNA sequences generated in both seeded and unseeded reactions showed no homology to either TPR (+) or (−) strands. These sequences were extremely diverse (~95% unique) and showed no evidence of convergence or complementarity among themselves (Supplementary Fig. 9). Across iterative cycles, their abundance increased and their composition shifted towards a more GC-rich pattern (Fig. 5f,g) reminiscent of the TPR’s substrate preferences^4^ and the influence of triplet composition upon strand reannealing (Extended Data Fig. 3). However, unlike a previous template selection experiment (using a mononucleotide RNA polymerase ribozyme), where products became biased towards increasingly G-rich sequences^23^, here the relative proportions of G to C as well as of A to U remained both stable and closely matched, following Chargaff’s rule^25^. This strongly suggests that these emerging RNA sequences were propagated by synthesis in a templated process^26^. We hypothesize that the gradual emergence of these diverse RNA sequence pools indicates a de novo sequence generation by the TPR followed by mutual templating and priming, as well as a mix of partial replication and recombination (as seen in Extended Data Fig. 8). In contrast to the products observed from primer-dependent random-sequence replication (Fig. 3b), RNAs in this pool increased in size and complexity upon open-ended cycling. In summary, our data show how randomly triplet-primed RNA replication driven by pH/freeze–thaw cycles can support fragmentary and distributive TPR self-replication as well as the accompanying de novo generation of diverse RNA sequence pools.

## Conclusions

We have shown that RNA trinucleotide triphosphates (triplets) provide a plausible solution to the strand separation problem by acting simultaneously as RNA chaperones (by stabilizing RNA oligomers in single-stranded form and attenuating strand reannealing) and as substrates and initiators (primers) of RNA replication. Combined with coupled pH, temperature and concentration gradients, here this enables exponential replication of defined- and mixed-sequence double-stranded RNA sequences. When extended to primer-free amplification of random RNA sequence pools, this led to emergent de novo sequence generation and partial self-replication, with the TPR spontaneously copying stretches of its own sequence.

All of these outcomes are consequences of the physicochemical properties of the triplet substrates, which form a web of interactions both with the template strands and themselves. Triplet:triplet base-pairing interactions^4^ probably underpin both terminal transferase activity (Extended Data Fig. 5) and the ‘creative mode’ of de novo sequence generation (Fig. 5g and Supplementary Fig. 9). Triplet–triplet stacking interactions drive cooperative triplet binding, enabling primer-free synthesis (Extended Data Fig. 4) and blocking reannealing on templates with ≥50% GC (Fig. 1d). The principles for overcoming the strand separation problem described herein are not dependent on the nature of either the catalyst or a specific geochemical environment (for example, bedrock chemistry) and therefore could probably apply to non-enzymatic RNA replication^1^, where di- and trinucleotides have been shown to act as functional substrates^27^.

Despite the capacity of RNA triplets to hybridize to RNA strands and prime RNA synthesis as well as invade RNA secondary structure^4^, we find that the TPR appears surprisingly resistant to inhibition by them, even in the presence of random triplet (^ppp^NNN) pools and multiple cycles of denaturation (Figs. 3b and 5b), presumably due to either high stability or rapid refolding of the TPR structure. Indeed, the TPR appears largely resistant to invasion, even by a 29-nt complementary internal segment (γ^−^) (Fig. 4). However, the γ segment is part of the stable and highly ordered core of the TPR as judged by cryo-electron microscopy^21^, and it is currently unclear whether more flexible segments of the TPR (such as, for example, the 5TU P10 domain) would be more vulnerable to sequestration. A future RNA replicase ribozyme will probably have to manage the tradeoffs of embodying both template and catalyst in one RNA molecule. Evidence of such tradeoffs is apparent in our replication experiments, where the intact TPR serves as only an inefficient template, but becomes a more efficient template as it begins to unfold and/or degrade upon prolonged cycling (Extended Data Fig. 8). As replication cycles proceed, triplet priming on such fragments creates a growing pool of first TPR (−) and then (+) strand-homologous products, providing a potential route to a fragmentary form of self-replication^28^ (Fig. 5h).

Beyond these TPR fragment pools, replication with all 64 triplet substrates also results in the de novo generation and amplification of diverse pools of RNA oligomer sequences (up to 60-nt long; Fig. 5). Such de novo sequence generation has been observed previously by proteinaceous RNA polymerases, notably Qbeta RdRP^29^ and T7 RNA polymerase^30^, via a variety of mechanisms. Further study to elucidate product origins may open a route to primer-free replication of defined sequences, although replicated sequences would likely need to exhibit a selectable phenotype to persist^31^. In this way, pools of amplification products could yield new activities to promote ribozyme survival.

**Extended Data Fig. 10:**
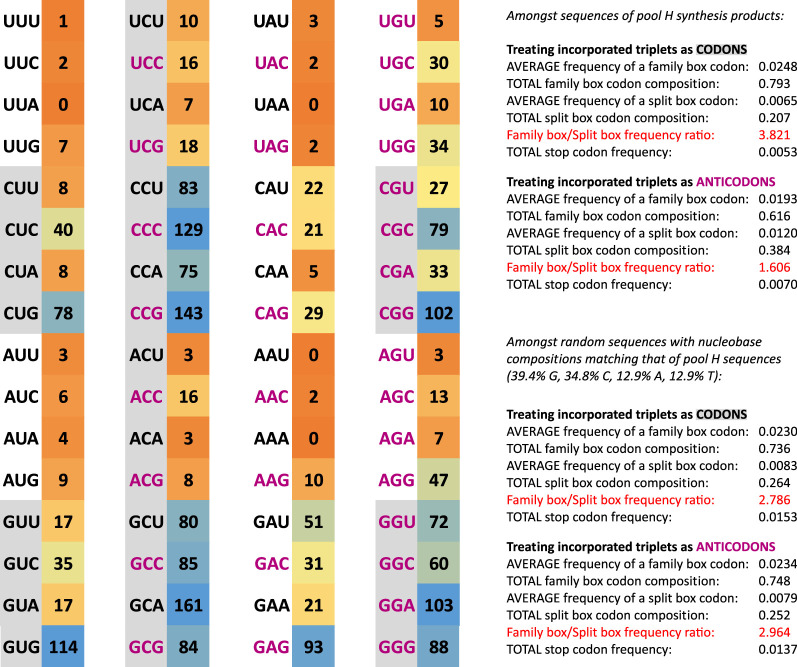
Compositional bias of ribozyme-synthesised amplification products towards family box codons. Left, Estimated percentages of each triplet substrate incorporated into amplification products in the 73-cycle unseeded amplification reaction (Fig. 5b). The triplet composition of pool H products (Extended Data Fig. 9a) was multiplied by the observed yield of RNA product in the reaction (the equivalent of 284.5 pmols of triplets incorporated out of 800 pmols supplied; see Fig. 5c) to estimate the consumption of each triplet (from the 12.5 pmols of each of the 64 triplets available in the analytical sample). Consumption %s are shown to the right of each triplet sequence, which show strong biases: some triplets are barely incorporated, others are almost fully consumed. Those triplets’ consumptions calculated to exceed 100% may reflect sequence/structure/length preferences of RNA intercalation, adapter ligation and/or RT-PCR in the work-up, not unusual when sequencing short RNAs^43^. GC-rich triplets are particularly depleted, and their high degree of utilisation might also reflect their better capacity to initiate RNA synthesis on the growing product pool (Extended Data Fig. 4) and more effective inhibition of strand reannealing (Extended Data Fig. 3). Triplets highlighted in grey represent family box codons (FBC) in the genetic code, encoding the same amino acid independently of their third position. Triplets written in purple represent the anticodons of FBCs. Right, Correlations between these classifications and triplet usage. TPR RNA products are 3.8-fold enriched in family box codons, which are thought to have comprised an early genetic code. There are contributions from both nucleobase composition (random sequences with identical nucleobase composition yield a less pronounced 2.8-fold FBC preference) and triplet sequence preferences (there is just a 1.6-fold preference for family box anticodons which have inherently identical GC compositions). TPR-generated amplification products therefore would likely generate longer peptides when translated using a putative primordial genetic code (assuming identical triplet / codon register). As may be expected based upon their base composition, triplets corresponding to modern stop codons were rarely incorporated.

Analysis of the amplified product pools in primer-free replication reveals a striking drift (~80%) towards sequences composed of triplets corresponding to family box codons in the genetic code (Extended Data Fig. 10). Family box codons (1/2 of all codons) encode amino acids independently of their third nucleobase, and these have been proposed to have formed part of a simpler, primordial genetic code, as they (generally) form more stable codon–anticodon interactions^32^, and encode amino acids provided by potentially prebiotic chemistry^33,34^. Their observed selective enrichment by replication probably reflects both TPR sequence preferences (for GC-rich triplets) as well as thermodynamic considerations (that is, higher template occupancy to inhibit strand reannealing), which have been shown to influence sequence pool evolution in model replication experiments^11^ (Fig. 3). It is tempting to speculate that the same physicochemical principles that introduce sequence bias into triplet-based RNA replication may have shaped an early genetic code to potential mutual benefit. Through a drift in triplet usage, replication cycles not only drive RNA sequence pools towards more effective replication, but also more productive translation using a primitive genetic code (Fig. 5d). This could have played an important role in the emergence of coded peptide-based phenotypes.

The factors that shape the emergence and evolution of preferentially replicated sequences from a random template (Fig. 3) may also underlie differential (+)- and (−)-strand replication (Fig. 2c). Viruses^35^ and viroids^36^ (considered by some to be relics of the RNA World) show evidence of a ‘division of labour’ between their strands, where asymmetry in secondary structure formation and folding energy favours one strand for encoding function and the other for replication. Similar specialization may manifest in RNA-catalysed RNA replication.

Although we had not set out to investigate prebiotic RNA replication scenarios, we note that our RNA replication strategy is general and robust over a range of temperature and pH values (Extended Data Figs. 2 and 6), including flash-freezing^37^, present in modern geothermal fields^38^. Functionally similar physicochemical gradients can be found in alternative geochemical scenarios including evaporation–condensation cycles or thermophoretic pH and concentration gradients^13,39,40^. Notably, freeze–thaw cycles have also been shown to promote RNA folding^41^, and recently to facilitate both activation and non-enzymatic polymerization of ribonucleotides^2^, as well as RNA 2′,3′-aminoacylation^42^.

In summary, we describe a general mechanism by which the conflicting roles of RNA oligomers as general replication substrates, as unfolded replication templates (and primers) and as folded replication catalysts can be plausibly reconciled. Our work defines physicochemical parameters capable of overcoming the strand separation problem and suggests that RNA self-replication—generating fragments for later assembly^26,28^—arises as a predisposed, emergent property of randomly primed RNA replication.

## Methods

Nucleic acid sequences are listed in Supplementary Table 2, and RNA synthesis techniques in the Supplementary Methods.

### Ribozyme RNA polymerase assay

Standard (non-replicative) primer extension assays were conducted in 5 µl of extension buffer (final concentrations 0.1 M MgCl_2_, 50 mM Tris·HCl pH 8.3, 0.05% Tween-20, 100 nM of each primer, 2.5 µM of each triplet/oligomer, 0.5 µM ribozyme). To start, ribozyme was annealed (80 °C, 2 min; 17 °C, 10 min) in 1 µl of water. Meanwhile, 0.5 pmol of template or duplex was incubated in 2 µl of 0.05% Tween-20 (+5 mM HCl for ‘acidified’ reactions) at 25 °C for 10 min. All other reaction components (in 2 µl) were mixed with ribozyme then template fractions, before flash-freezing in liquid N_2_ (20 s) and incubation at −7 °C in an LTC4 refrigerated bath (Grant Instruments).

To measure the effect of delayed triplet and/or TPR addition (for example, Fig. 1d), duplex or template was acidified as above, then a neutralization mix was added with or without the relevant triplets and primers. This neutralization mix also contained enough MgCl_2_, Tris·HCl pH 8.3 and Tween-20 to yield their final concentrations above. The resulting volume (2.5–3.7 µl) was immediately flash-frozen in liquid N_2_ and incubated at −7 °C to allow the eutectic phase to thaw out, during which time the template strand(s) reannealed or became coated with triplets.

After the indicated delay, more buffer (with identical MgCl_2_/Tris·HCl pH 8.3/Tween-20 concentrations) containing the TPR (with any missing triplets and primers) was added on top of the frozen volume to a final volume of 5 µl at −7 °C. This made all total reaction compositions identical (except template), though the buffer with TPR froze as a distinct layer on top of the original ice; the eutectic phases of the layers, however, became contiguous, allowing TPR (±triplets) to diffuse into the lower layer containing the template (as previously reported^22^). Reactions were then incubated at −7 °C for 48 h to allow primer extension, and the fraction of primers extended was then used to deduce the available template levels (and thus the degree of strand reannealing) as follows.

Data points with extension >0.1% were used to deduce reannealing rates in the eutectic phase. Even in standard reactions, primer extension is not complete, and (independent of strand reannealing) a two-layer reaction with triplets separated from the template gave threefold less primer extension than when triplets began in the same layer as the template (Fig. 1d). Therefore, primer extension efficiencies on the duplex were first divided by the average efficiency with template alone (from equivalent reactions with or without triplets at neutralization—or a geometric mean thereof when half were present). Efficiencies were then converted into free strand concentrations by estimating the eutectic phase volume (see below). As triplets/TPR do not immediately complete diffusion between reaction layers, there is a hidden lag phase, and extrapolated no-delay efficiencies would vary between different conditions. Nonetheless subsequent reannealing rates could be estimated using the changes observed between delays of different lengths.

### Replication cycling

Iterative replication of RNA was undertaken in 0.5-ml microfuge tubes containing 125 µl of replication buffer (standard composition: 0.4 mM MgCl_2_, 1.8 mM KCl, 1 mM N-cyclohexyl-2-aminoethanesulfonic acid (CHES)·KOH pH 9.0, 0.01% Tween-20, 20 nM TPR, 100 nM of each triphosphorylated triplet, 100 nM each of any RNA primers/oligomers). Up to 1 pmol of starting RNA template or duplex to be amplified was added per reaction (Extended Data Fig. 6 provides a discussion of this parameter). Reactions were prepared at room temperature, and cycling was begun with an initial denaturing step where 0.75 µl of 0.1 M HCl was added (+0.6 mM in the reaction, overwhelming the CHES buffer), the reaction was vortexed, and then incubated for 2 min on a thermocycler preheated to 80 °C. Next, 0.75 µl of 0.1 M KOH was added, and the reaction was briefly vortexed, then plunged in liquid N_2_ for 20 s to flash-freeze, before incubation at −7 °C for ~24 h. Another cycle was initiated by thawing the reactions at room temperature before HCl addition, and so on.

The fold concentration of solutes within the eutectic phase upon freezing of extension buffer or replication buffer was calculated by estimating the eutectic phase volume as a fraction of the ice, as described previously^22^. Briefly, a sample was prepared with the ionic composition of the target reaction (for extension buffer) or a 50-fold concentrated version (for replication buffer), alongside a gradually more concentrated set of samples with the same amount of buffer components but lower reaction volumes. These were then flash-frozen and incubated at −7 °C to allow eutectic phase equilibration. If the volume of a concentrated sample was less than that of the eutectic phase of the parent sample, no ice phase would be present at equilibrium. This transition occurred in a tenfold concentrated sample for extension buffer, and an 8.8-fold concentrated sample for 50× pre-concentrated replication buffer. Assuming that the eutectic phase composition was the same, independent of the starting volume (that is, uniform freezing point depression from solutes), linear concentration factors were applied (that is, 440-fold concentration from freezing replication buffer at −7 °C).

Open-ended replication cycling involving serial dilution proceeded as shown in the relevant figures (Figs. 2b, 3a and 5a). The dilution protocol was designed to create an appropriate replication burden (two- to threefold amplification in four or five cycles), while maintaining the KCl concentration (increasing from pH cycling) within the 1.8–4.2 mM range. To dilute, an acidified sample was neutralized post-heating by the combined addition of chilled KOH and fresh reaction mix, then flash-frozen and incubated at −7 °C. After this incubation, the reaction was thawed and a 125-µl aliquot transferred to a fresh tube for further cycling. The remnant sample was retained for analysis.

In some lanes of Extended Data Fig. 8a, half of the TPR was degraded beforehand by incubation at 80 °C for 8 min in 20 mM MgCl_2_/30 mM KCl/50 mM CHES·KOH pH 9.0/0.02% Tween-20. The remaining reaction components were then added to this mixture to restore the standard reaction composition before cycling began.

### Denaturing gel electrophoresis

For gel analysis of RNA synthesis in extension buffer, the samples were quenched with excess ethylenediaminetetraacetic acid (EDTA) over Mg^2+^, adding urea to 6 M. Where a specific template sequence was included in the reaction, a tenfold excess of a complementary RNA was also provided to outcompete product:template rehybridization. Samples were denatured (94 °C, 5 min), cooled and separated on 20% acrylamide/8 M urea/TBE gels. For analysis of RNA synthesis in replication buffer, 125-µl reaction aliquots were first ethanol-precipitated (85% ethanol final concentration, with glycogen carrier) before resuspension in water and addition of EDTA and urea as above.

The reactions in Supplementary Fig. 7a with biotinylated primer were stopped with excess EDTA and mixed with two volumes of bead-wash (BWBT) buffer (200 m NaCl, 10 mM Tris·HCl pH 7.4, 1 mM EDTA, 0.1% Tween-20), then biotinylated primers and products were bound to MyOne C1 streptavidin-coated microbeads (Invitrogen) prewashed three times in BWBT. These were then washed with BWBT, templates were removed with a 50 mM NaOH/1 mM EDTA/0.1% Tween-20 wash, the beads were neutralized with a BWBT + 100 mM Tris·HCl pH 7.4 wash, before a final BWBT wash and resuspension in 95% formamide/25 mM EDTA. After heating at 94 °C for 5 min to denature and detach the primers from the beads, the supernatant was subjected to denaturing polyacrylamide gel electrophoresis (PAGE) to separate the primers.

Gels were scanned on a Typhoon FLA-9000 imager (GE) at different wavelengths for each fluorophore-labelled primer. The gel of primerless extensions in Fig. 5b was pre-stained with SYBR Gold (Invitrogen) before scanning. Gel bands were quantified using ImageQuant analysis software; backgrounds were drawn between peak troughs or using adjacent negative controls. For primer extensions, band intensities of unextended primers and extension products of all lengths were used to calculate the product distribution, and hence extension yields of, for example, ‘full-length’ (full-length band only), ‘reaching full-length’ (including longer bands) or ‘primer extension’ (at least 1–3 triplet addition) products, as indicated in the figure legends, with product pmol or µM calculated from the known total pmol or µM of primer in each reaction.

To estimate the amplified RNA yield (Fig. 5), the lane intensities in Fig. 5b beneath the TPR bands were measured by densitometry, and the corresponding ^ppp^NNN-free reaction backgrounds were subtracted (alongside, if present, N_20_ seed band intensities). These intensities were converted to RNA product yields using the N_20_ seed band intensity as a reference (Fig. 5b, left lane), treating the fluorescence of SYBR Gold-stained RNAs as proportional to their length.

Oligonucleotides were purified from PAGE gels by the excision of bands identified by UV shadowing or alignment with a fluorescence scan. The bands were crushed and the oligonucleotides therein eluted into 10 mM Tris·HCl pH 7.4. Gel fragments were removed from the supernatant by passage through a Spin-X 0.22-µm cellulose acetate filter (Costar) and the supernatant was precipitated in 73% ethanol (ribozymes or long oligonucleotides) or 85% ethanol (oligonucleotides <8 nt).

### RNA sequencing

The techniques and strategies used to sequence RNA synthesis products are described in the Supplementary Methods. Scripts used to analyse the sequencing data are listed in Supplementary Table 3.

## Online content

Any methods, additional references, Nature Portfolio reporting summaries, source data, extended data, supplementary information, acknowledgements, peer review information; details of author contributions and competing interests; and statements of data and code availability are available at 10.1038/s41557-025-01830-y.

## Supplementary information

Supplementary Information Table of contents, Supplementary Figs. 1–9, Tables 1–3, Methods, References, Source data for Supplementary Figs. 1–3 and 5–7.

## Source data

Source Data Fig. 1 Uncropped gels for Fig. 1c and graphed values for Fig. 1d.

Source Data Fig. 2 Uncropped gels for Fig. 2c.

Source Data Fig. 3 Uncropped gels for Fig. 3b and 3d and graphed values for Fig. 3c and 3e.

Source Data Fig. 4 Uncropped gels for Fig. 4b and 4c and graphed values for Fig. 4c.

Source Data Fig. 5 Uncropped gel for Fig. 5b, graphed values for Fig. 5c and charted values for Fig. 5d.

Source Data Extended Data Fig. 1 Uncropped gel for Extended Data Fig. 1b.

Source Data Extended Data Fig. 2 Graphed values for Extended Data Fig. 2b and uncropped gel for Extended Data Fig. 2c.

Source Data Extended Data Fig. 3 Uncropped gel for Extended Data Fig. 3.

Source Data Extended Data Fig. 4 Mapped values for Extended Data Fig. 4.

Source Data Extended Data Fig. 5 Uncropped gels for Extended Data Fig. 5a and graphed values for Extended Data Fig. 5b.

Source Data Extended Data Fig. 6 Uncropped gel and graphed values for Extended Data Fig. 6.

Source Data Extended Data Fig. 7 Uncropped gels and graphed values for Extended Data Fig. 7.

Source Data Extended Data Fig. 8 Uncropped gels for Extended Data Fig. 8a and charted values for Extended Data Fig. 8b.

Source Data Extended Data Fig. 9 Amplification factors used for Extended Data Fig. 9d.

## Data Availability

Sequencing data and analysis summary files are publicly available from OSF.io at https://osf.io/whz92/?view_only=2984646952514752a62a4ecda73c089d. Source data are provided with this paper.

## Code availability

Custom scripts supporting the analysis of triplet-based replication products are publicly available in the GitHub repository at https://github.com/JamesAttwater/RNArepseq, https://github.com/holliger-lab/fidelity-analysis.
