# Supplementary material for: Trinucleotide substrates under pH–freeze–thaw cycles enable open-ended exponential RNA replication by a polymerase ribozyme
Source: Nat Chem. 2025 May 28;17(7):1129–37. doi: 10.1038/s41557-025-01830-y (PMC12226338; doi:10.1038/s41557-025-01830-y)
Supplement: Supplementary file 1 — Table of contents, Supplementary Figs. 1–9, Tables 1–3, Methods, References, Source data for Supplementary Figs. 1–3 and 5–7. [file 41557_2025_1830_MOESM1_ESM.pdf]

nature chemistry

Supplementary information

https://doi.org/10.1038/s41557-025-01830-y

Trinucleotide substrates under pH–freeze–thaw cycles enable open-ended exponential RNA replication by a polymerase ribozyme

In the format provided by the authors and unedited

Supporting Information - Table of Contents:

Supplementary Figure 1 1

Supplementary Figure 2 2

Supplementary Figure 3 3

Supplementary Figure 4 4

Supplementary Figure 5 5

Supplementary Figure 6 6

Supplementary Figure 7 7

Supplementary Figure 8 9

Supplementary Figure 9 10

Supplementary Table 1 11

Supplementary Table 2 12

Supplementary Table 3 15

Supplementary Methods 17

Supplementary References 22

Source Data for Supplementary Figure 1 23

Source Data for Supplementary Figure 2 24

Source Data for Supplementary Figure 3 26

Source Data for Supplementary Figure 5 27

Source Data for Supplementary Figure 6 28

Source Data for Supplementary Figure 7 29

Supplementary Figures

a

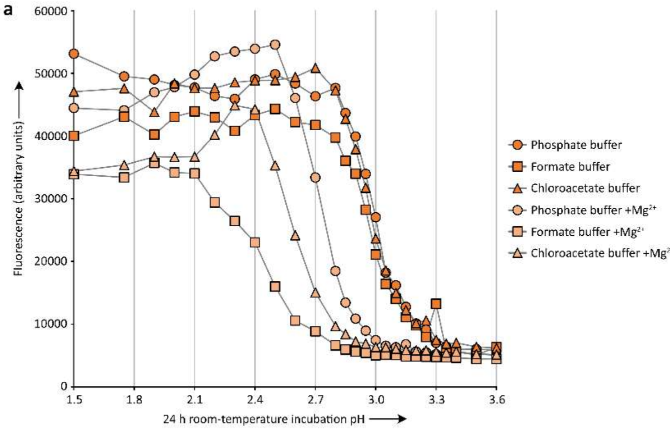

b

pH:	Incubation temperature: 25°C	37°C	50°C	60°C	80°C
1	93	151	283	452	
	±0.4	±0.5	±0.4	±1.8	
2.4	76	**70**	86	125	
	±0.8	±0.9	±0.8	±0.5	
2.7	65	63	73	90	
	±0.7	±0.9	±0.4	±1.0	
3	66	61	**67**	77	
	±0.8	±1.1	±0.4	±0.6	
3.3	9	42	71	101	
	±0.3	±1.5	±0.5	±1.0	
3.6	7	6	24	62	**100**
	±0.4	±1.2	±0.5	±0.2	

Supplementary Fig. 1 Strand separation under different pH and temperature regimes.

(**a**) Strand separation of GC-rich RNA duplexes at low pHs measured by the fluorescence/quench assay (Extended Data Fig. 2a, 0.1 M NaCl/0.05% Tween-20 detergent/0.1 M of phosphate, formate or chloroacetate buffers at the indicated pHs, 24 h at room temperature). Lowering the pH below 3 drove a sharp strand separation transition at room temperature. This is substantially below the pK_as of the nucleosides adenosine (4.5) and cytidine (4.0), potentially reflecting pK_a shifts due to base pairing in double-stranded RNA, and the need for cooperative protonation to disrupt a long duplex. A second set of samples (+Mg^{2+}) with 20 mM $MgCl_2$ also present in the buffer during denaturation showed a requirement for even lower pHs for strand separation; furthermore, we observed that 20 mM $MgCl_2$ at such pHs induced aggregation and precipitation of longer RNAs such as the triplet polymerase ribozyme. (**b**) Combinations of temperature and pH that are capable of denaturing the A^D RNA duplex, assayed as in Extended Data Fig. 2b and expressed (after subtracting background) as a % of signal vs. a fully-denaturing 2-minute incubation of 80°C at pH 3.6 (n = 3 independent repeats ± s.d.). Percentages above 100% likely reflect degradation of RNA and release of fluorescein from the oligonucleotide, relieving nucleotide-mediated quenching otherwise present in all duplexes. Conditions in bold/orange were chosen to drive test replication cycles in Extended Data Fig. 2c; although gentler conditions gave high signal in this strand reassortment assay, these consistently led to lower replication yields. We hypothesize that although milder pH and temperature regimes are able to dissociate duplex RNAs, under these conditions single-stranded oligomers are in rapid equilibrium with the duplex state (despite the low pH), leaving only a fraction single-stranded template available for ribozyme-catalyzed replication at the point of neutralisation/freezing.

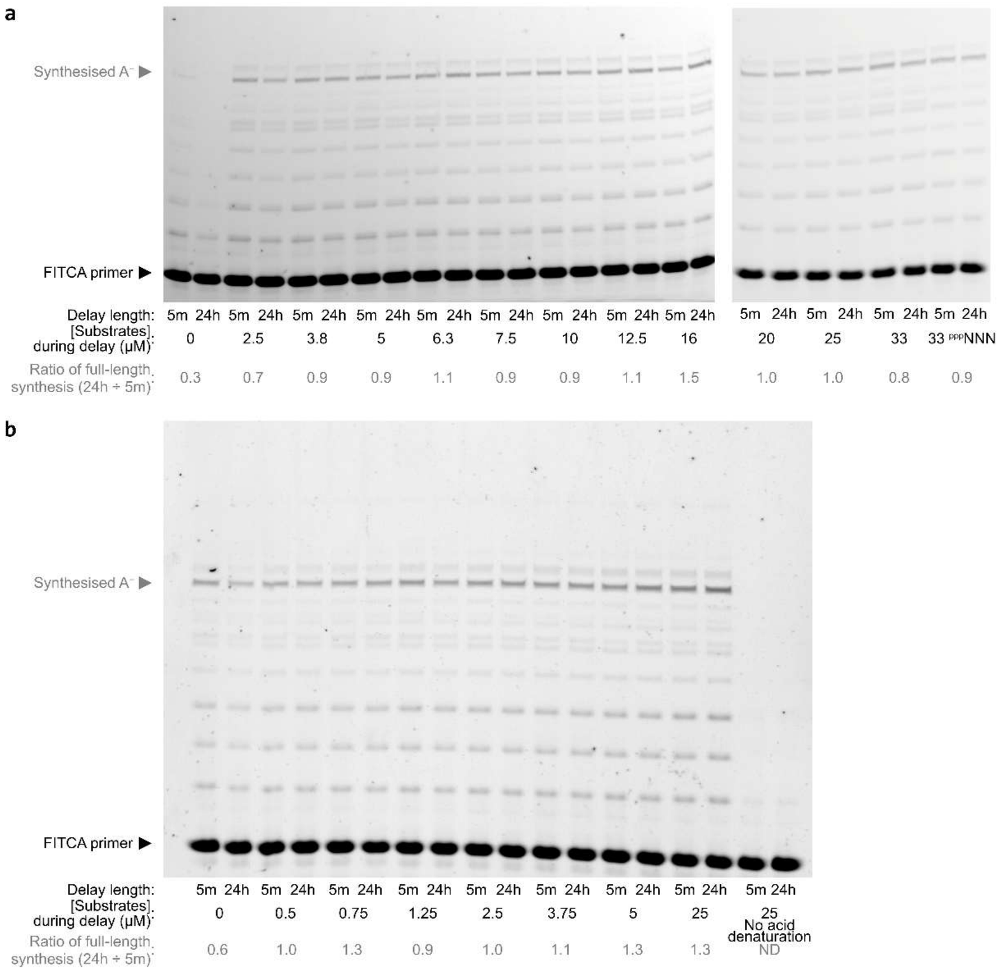

Supplementary Fig. 2 Influence of triplet substrate concentration upon the inhibition of strand reannealing.

(**a**) Primer extension on acid-denatured RNA duplex templates, following delayed addition of varying amounts of substrates after neutralisation. Reactions were set up as in Fig. 1d, but with different concentrations of triplets present upon neutralisation and freezing (shown are estimated eutectic phase triplet concentrations after neutralisation). After a delay of 5 min or 24 h, TPR ribozyme was added (alongside remaining substrates, fixing a final eutectic phase concentration of 25 μM each triplet & 1 μM duplex) before continued incubation at -7°C. Each pair of lanes (at different triplet concentrations) shows the proportional reduction in products reaching full-length as a result of a longer delay in reannealing, as calculated by gel densitometry. Little reannealing appears to occur even down to low triplet concentrations. pppNNN: Instead of just the cognate triplet substrates (as in Fig. 1b), all 64 triplets were added as substrates; the strand coating effect was maintained. (**b**) As in (a) but with lower concentrations of duplex (0.5 μM final concentration in the eutectic phase), and lower substrate concentrations during the post-neutralisation delay (down to 0.5 μM of each, all restored to 25 μM of each upon ribozyme addition). At these lower duplex concentrations, strand reannealing is incomplete, but the presence of stoichiometric triplet substrates restores strand synthesis to full levels even when TPR ribozyme addition was delayed for 24 h.

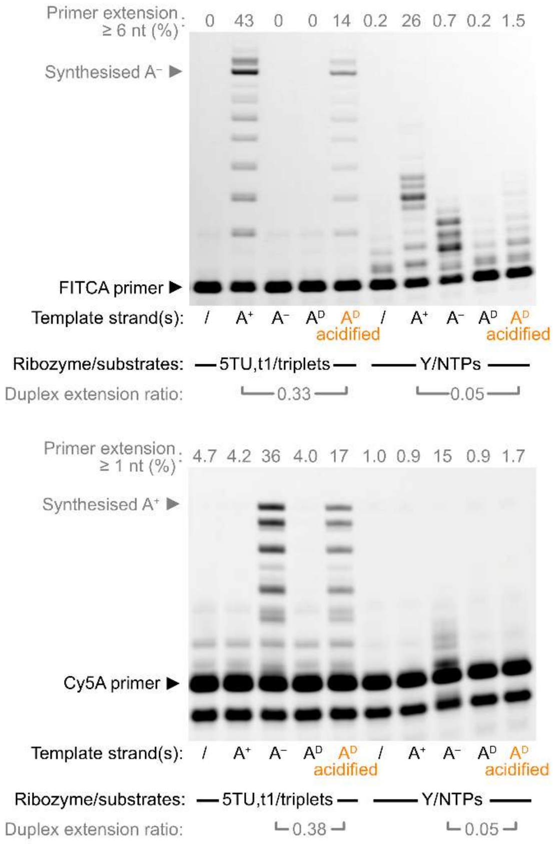

Supplementary Fig. 3 Primer extension activity of triplet polymerase ribozyme versus monomer polymerase ribozyme using RNA duplex templates.

Denaturing PAGE of primers (0.1 µM each FITCA (imaged above) and Cy5A (imaged below)) extended upon different A templates (0.1 µM) in extension buffer (100 mM $MgCl_2$, 50 mM Tris·HCl pH 8.3, 0.05% Tween-20). This was catalysed by (left) 0.5 µM TPR (with 2.5 µM of each triplet in the Fig. 1b synthesis scheme, -7°C 2 days), or (right) using 0.5 µM mononucleotide polymerase ribozyme Y[23] (with 0.5 mM of each of the four nucleoside triphosphates (NTPs), -7°C 12 days). TPR lanes reproduced in Fig. 1c. Yields of primer extension (≥ 6 nt (upper panel) or ≥ 1 nt (lower panel)) in each lane were estimated by gel densitometry. Very little primer extension was achieved by acidification of duplex when using NTPs as substrates compared to triplets, as judged by the duplex extension ratio on each template strand, calculated using estimates of '% extension above background' from densitometry:

('A^D acidified' – '/') ÷ ('$A^{+ \text{ or } -}$' – '/')

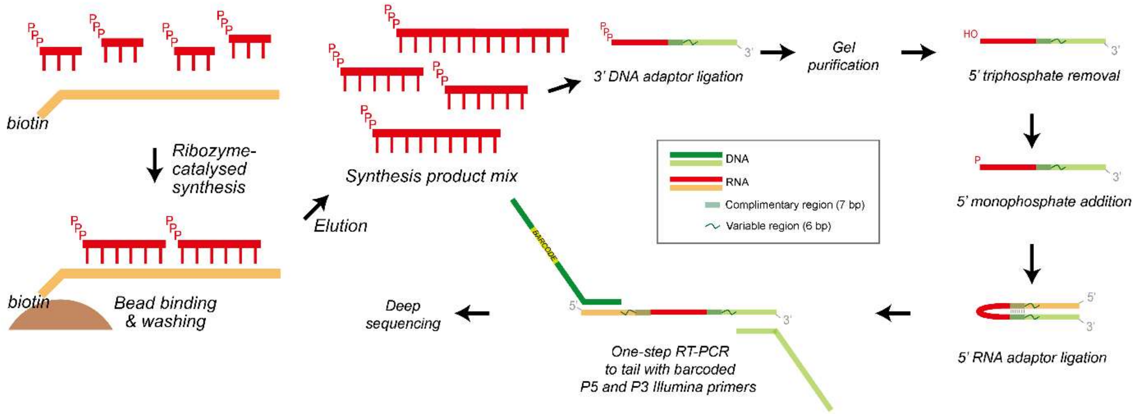

Supplementary Fig. 4 Sequencing of synthesis intermediates.

Sequencing scheme for identification of synthetic intermediates (Extended Data Fig. 4) during templated synthesis of ribozyme fragments using template-complementary triplets alone (See Supplementary Methods for experimental details).

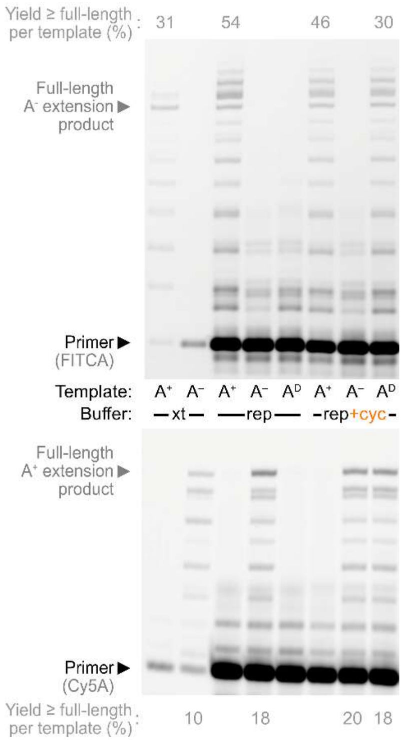

Supplementary Fig. 5 Single strand vs duplex replication efficiency in extension and replication buffer.

Strand synthesis efficiency upon single-stranded (ss) template in replication buffer matches that in extension buffer (in which the ribozyme was originally evolved[4]). Implementation of a pH cycle in replication buffer has little effect on extension on a ss template, but efficiently unlocks the templating activity of double-stranded RNA duplex. Extension buffer (xt): 100 mM $MgCl_2$, 50 mM Tris·HCl pH 8.3, 0.05% Tween-20, 0.1 µM of each primer and 2.5 µM of each triplet substrate in the scheme of Fig. 1b, 0.5 µM TPR, 0.1 µM template. These were frozen at -7°C for 48 h, increasing concentrations of solutes by ~10-fold in this eutectic phase[22]. Replication buffer (rep): 0.4 mM $MgCl_2$, 2.4 mM KCl, 1 mM CHES·KOH pH 9.0 @ 25°C, 0.01% Tween-20, 0.1 µM of each primer and 0.1 µM of each triplet substrate in the scheme of Fig. 1b, 20 nM TPR, 4 nM f.c. template; -7°C frozen 48 h, ~440-fold solute concentration in the eutectic phase. Replication buffer after cycling (rep+cyc): as rep but with addition of 0.6 mM HCl, incubation at 80°C for 2 min, and addition of 0.6 mM KOH before flash-freezing and incubation. Densitometry was used to estimate full-length product yield per template. Lower template concentrations were used with replication buffer to reflect the levels used in other replication experiments throughout this paper.

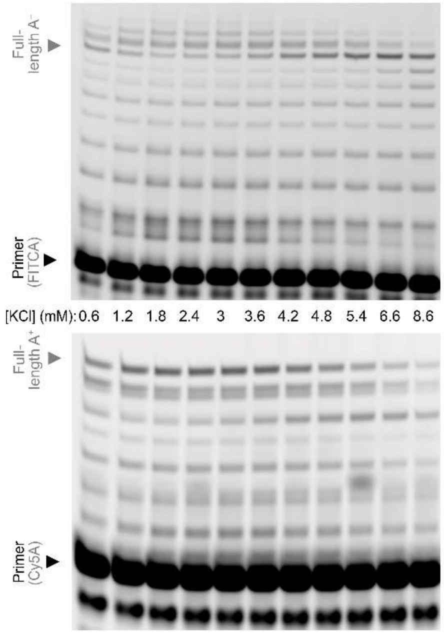

Supplementary Fig. 6 Inhibition of extension by KCl buildup during cycling.

Accumulation of KCl during cycling moderately inhibits extension on sequence A. Shown are single cycles of replication with varying concentrations of KCl ([KCl]), but otherwise set up as in Fig. 2c, with 4 nM starting duplex A^D. The [KCl] shown includes the 0.6 mM increase resulting from the single cycle of acidification and neutralisation. Freezing is predicted to yield molar ionic concentrations in the eutectic phase, and at the highest [KCl], less ice crystal growth may occur as eutectic composition is reached earlier – effectively diluting other reaction components as a result and changing the pattern of primer extension. To attenuate this, iterative cycling (increasing [KCl] by 0.6 mM/cycle) underwent occasional dilution with fresh reaction mix lacking KCl (Fig. 2b).

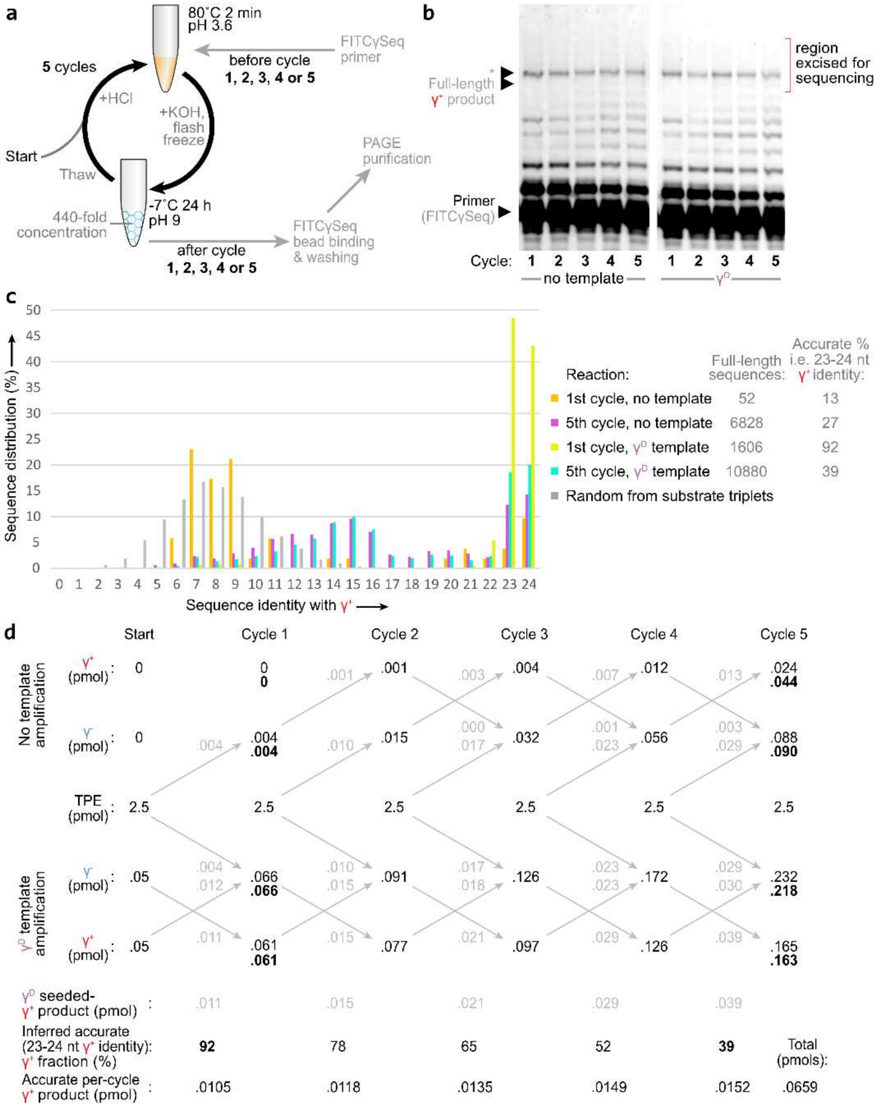

Supplementary Fig. 7 Cycle-by-cycle sequencing and analysis of self-synthesis replication products.

(**a**) Scheme for cycle-specific sequencing of replication products. Reactions were set up as in Extended Data Fig. 8a, but with 0.1 µM FITCγSeq biotinylated sequencing primer added during acidification before one of the 1st to 5th cycles. Reactions were then stopped after this single cycle of sequencing primer extension. These primers were then separated from the other FITCγ primers via biotin capture of FITCγSeq extension products, obtaining a cycle-specific record of primer extension. Iterative cycling with FITCγSeq alone was not performed as the lengthened 5' of this RNA (needed to provide an RT-PCR primer binding site of sufficient length for sequencing) reduced replication yields. (**b**) Denaturing PAGE separation of FITCγSeq primer extensions in (a). The indicated gel regions (containing full-length products) for cycles 1 and 5 in γ^D-seeded and no template reactions were excised and the primers therein ligated to 3' adapters, amplified by RT-PCR, and subjected to high-throughput sequencing. The major band present in this region (*) is a template-independent ligation artefact seen using sequencing primer; only full-length +24 nt products were further analysed. (**c**) Proportion of accurate replication products. The indicated number of such 24 nt length sequences were obtained from each sequencing reaction. These are plotted according to their degree of sequence identity with the expected γ^+

sequence. Also plotted is a distribution of sequence identity amongst 24 nt sequences generated *in silico* from a random concatenation of substrate triplets present in the reaction. Three classes of product are apparent. A single cycle of replication on γ^D generates accurate full-length γ^+ sequences (23-24 nt identity). Further cycles also generate products with partial identity (13-16 nt identity), potentially derived from template-switched incomplete extension products. Finally, there are sequences with no discernible identity with γ^+ (7-12 nt identity), the only output of a single cycle without template, where no γ^- sequence is present to direct γ^+ formation. (**d**) Model of replication product synthesis yields. To compare the absolute levels of sequence class synthesis *between* sequenced samples in (c), we needed to multiply the sequence distribution by the observed yields of full-length product in each cycle. Unfortunately, the artifact band (*) prevented quantification of FITCγSeq extension to full-length product in (b). We therefore modelled full-length product accumulation during the five cycles of seeded and unseeded replications - templated from product strands, TPR, and degraded TPR. For amplification constants, we used the γ^D templating efficiencies observed in the first cycle in Extended Data Fig. 8a (0.228-fold γ^+ from γ^-, 0.239-fold γ^- from γ^+, and 0.00167 γ^- from each TPR per cycle). An increase in the latter parameter of 0.0025 per cycle (to account for the increased templating capacity of degraded TPR that accumulates in the polymerisation steps) gave a reasonable match of the modelled yields to the observed yields (bold, from Extended Data Fig. 8a) after five cycles. Per-cycle yields of accurate γ^+ product formation were then estimated by interpolating the observed accuracies in the first and fifth cycles, which was then multiplied by the modelled per-cycle yields of all γ^+ products, giving an estimated synthesis yield of 0.065 pmols accurate γ^+ strand during five cycles of replication of 0.05 pmols γ^D. This yield does not include synthesis products exceeding full-length.

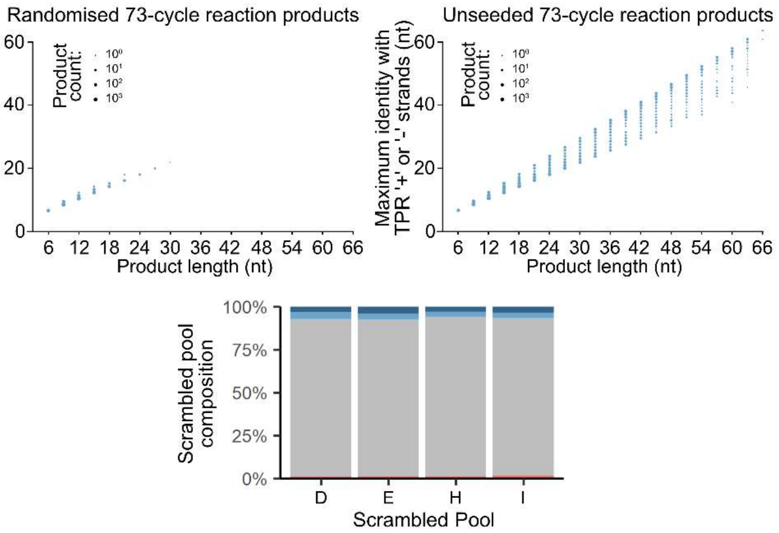

Supplementary Fig. 8 Nature of sequenced (+)-strand ribozyme-homologous products from exponential amplification.

Top, TPR identity amongst sequences classed as (+)-strand homologous. Sequence length is plotted against levels of (+)-strand identity for sequenced synthesis products from the unseeded 73-cycle reaction (right), and from a simulated pool of randomised RNAs of identical composition (left). Only sequences meeting the identity threshold for (+) or (+) & (-) strand homology are plotted; sequences with only (-)-strand identity are plotted in Fig. 5e. The 73-cycle reaction products contain two populations of sequences not present in randomised sequence. One population displays complete or near-complete (+)-strand identity together with a reasonably uniform length distribution. These likely derive from background sequencing of ribozyme (or degradation products thereof) despite the protocol requiring a 5' phosphate or triphosphate for RNA recovery: RNAs were treated with a pyrophosphohydrolase to selectively convert 5' triphosphates to monophosphates to promote selective ligation and sequencing of ribozyme-synthesised products. A second, distinct population of products (observed in the bimodality of homology in 21-36 nt length products, with ~14-28 nt (+) strand homology) has partial homology to the TPR, implicating ribozyme synthesis in their origins. Bottom, Negligible (+)-strand ribozyme homology is observed amongst scrambled pool sequences. 9 nt sequences were generated from random triplet assortments (matching the triplet compositions of the indicated pools) and classified as in Extended Data Fig. 9c.

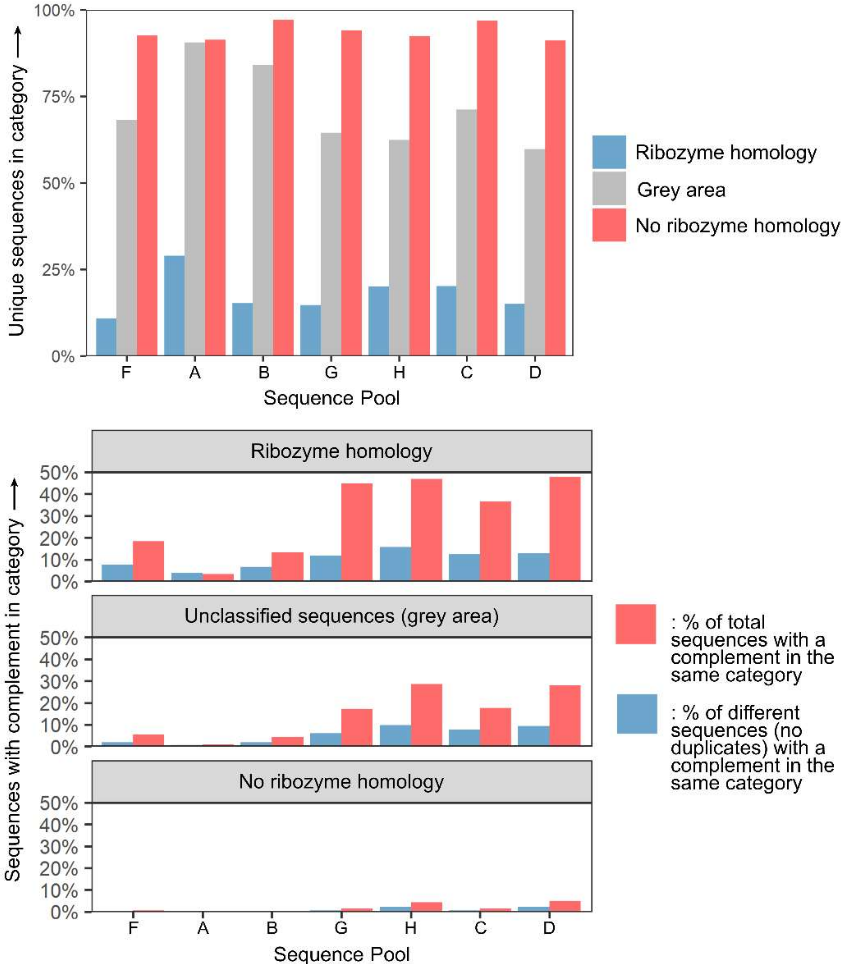

Supplementary Fig. 9 Relationships of RNA amplification products to one another.

Top, The percentages of each 9-27 nt sequenced replication product pool subcategory (classified by ribozyme homology as in Extended Data Fig. 9c) that comprise unique sequences. Across all samples, the majority of sequences with homology to ribozyme were present multiple times in the sequence data; the majority of those with no evidence of homology were unique. Bottom, Prevalence of complementary sequences among amplification reaction 9-27 nt products (from Extended Data Fig. 9), analysed by subcategory. This is expressed as the percentage of sequences in each replication product pool subcategory (classified by ribozyme homology) with a perfect complement in the same subcategory (red). This calculation was also performed after discarding repeat sequences in each subcategory, showing the percentage of different sequences with a complement in that subcategory (blue). The sequences unrelated to ribozyme showed little complementarity with each other, making reciprocal replication an unlikely mechanism for their amplification. Some of the complementarity among ribozyme-related ('+' and '-') sequences may arise from the presence of regions of internal complementarity within each ribozyme.

Supplementary Tables

Supplementary Table 1 – Replicated N_{17} library sequences

The ribozyme-catalysed replication of library in Fig. 3b was continued until 55 cycles had passed, before a single final cycle with a labelled sequencing primer to characterise the replicated RNAs. These extended primers were gel-purified in three length bins: +8-+11, +14-+17, and +20-+23 (full length equivalent), corresponding to replicons with 1-2, 3-4, or 5-6 triplets incorporated before the final pentamer. These were sequenced, and those sequences classed as potential replication products (the above lengths, ending in the 3' sequence UGUAGC – complementary to the reverse primer Cy5Rep) were tallied and the sequence of triplets incorporated to make them was determined. Examples of these, along with the number of times they appeared in the sequencing, are listed in the table below. Highlighted sequences are those tested in Fig. 3d (corresponding to Rep1+, Rep2+, Rep3+, Rep4+). The most common replicated sequences of each length show a bias towards constituting triplets of sequence 5'-^{ppp}SSW-3' (S =G/C, W = A/U), a pattern that can be accommodated on both strands due to the offset register of extension (Fig. 3a).

**Length bin +8-+11 incorporated triplets:**	**Occurrence:**
**-GGU-GUAGC**	181146
**-CGU-GUAGC**	117992
**-GCU-GUAGC**	49729
**-GGU-GCU-GUAGC**	3433
**-GCG-GGU-GUAGC**	2812
**-GGG-GCU-GUAGC**	2142
**Length bin +14-+17 incorporated triplets:**	**Occurrence:**
**-GGG-GCC-GGU-GUAGC**	2576
**-GGU-GCG-GGU-GUAGC**	2044
**-GGU-GCC-GGU-GUAGC**	1586
**-GGA-GCA-CGU-GUAGC**	450
**-GGU-GCG-CCA-CCU-GUAGC**	21
**-GCC-GGU-CCA-GGU-GUAGC**	20
**-GGG-GCU-GCC-GCU-GUAGC**	17
**-GGU-GUG-GCA-GGU-GUAGC**	6
**Length bin +20-+23 incorporated triplets:**	**Occurrence:**
**-GGG-GCC-GGU-CCA-GGU-GUAGC**	38
**-GGU-GCG-GCA-GCG-GGU-GUAGC**	20
**-GGU-GCG-CCA-CCG-GGU-GUAGC**	18
**-CCU-CCU-CGC-GGU-CCA-GGU-GUAGC**	10
**-GGU-GUG-GCA-GGU-CCA-CGU-GUAGC**	4
**-GCC-GGG-GUC-CCG-GGG-GCU-GUAGC**	4
**-GAG-GCG-GCA-GCC-GCG-GGU-GUAGC**	3

Supplementary Table 2 – Oligonucleotide sequences

Oligonucleotide sequences are written 5' to 3' below; DNA sequences are in grey, RNA sequences are in black. RNAs synthesised from DNA sequences were prepared by methods 1-6 as described in the Supplementary Methods section. All oligonucleotides (except DNAs used to make transcription templates) were PAGE-purified before use and concentrations calculated using a Nanodrop spectrophotometer and sequence-specific 260 nm extinction coefficients derived using OligoCalc[44].

Oligonucleotide	*Sequence*	*Source*
**Fγ^+m1**	FITC-GAUGCAGAGGCGGCAGCCUUCGGUGGC	IDT
**Qγ^-m1**	GCCACCGAAGGCUGCCGCCUCUGCAUC-IABkFHQ	IDT
**Mγ^+m1**	^{ppp}GAUGCAGAGGCGGCAGCCUUCGGUGGC	**Method 1**
	GATCGATCTCGCCCGCGAAATTAATACGACTCACTATAGATG<br>CAGAGGCGGCAGCCTTCGGTGGCGGGTCGGCATGGCATC	Sigma
**Mγ^-m1**	^{ppp}GCCACCGAAGGCUGCCGCCUCUGCAUC	**Method 1**
	GATCGATCTCGCCCGCGAAATTAATACGACTCACTATAGCCA<br>CCGAAGGCTGCCGCCTCTGCATCGGGTCGGCATGGCATC	Sigma
**HDVrt**	CTTCTCCCTTAGCCTACCGAAGTAGCCCAGGTCGGACCGCG<br>AGGAGGTGGAGATGCCATGCCGACCC	Merck
**HDVRec**	GATGCCATGCCGACCC	Merck
**HPrepseq**	GACTCTTCGGAGTCTG	Merck
**5T7**	GATCGATCTCGCCCGCGAAATTAATACGACTCACTATA	Sigma
γ^+	CGGAUGCAGAGGCGGCAGCCUUCGGUGGC	IDT
γ^-	GCCACCGAAGGCUGCCGCCUCUGCAUCCG	IDT
**A$^-$**	CAACCGGGGGAAGCGGGGAGUCGGGUGGUG	IDT
**A$^+$**	CACCACCCGACUCCCCGCUUCCCCCGGUUG	IDT
**B$^-$**	Biotin-C9spacer-AUUUACCAGGCCAGGGUUCAGUUCAGAGAC	IDT
**B$^+$**	GUCUCUGAACUGAACCCUGGCCUGGUAAAU	IDT
**C$^-$**	AUUACGAAGGAAGGUUUGGUAUGGGAUAGU	IDT
**C$^+$**	ACUAUCCCAUACCAAACCUUCCUUCGUAAU	IDT
**Rep1+**	GGUCCACGUGUAGC	IDT
**Rep1-**	UGCUACACGUGGACC	IDT
**Rep2+**	GGUCCAGGUGCUGUAGC	IDT
**Rep2-**	UGCUACAGCACCUGGACC	IDT
**Rep3+**	GGUCCAGGAGCACGUGUAGC	IDT
**Rep3-**	UGCUACACGUGCUCCUGGACC	IDT
**Rep4+**	GGUCCAGGUGUGGCAGGUGUAGC	IDT
**Rep4-**	UGCUACACCUGCCACACCUGGACC	IDT
**5TU**	^{p}GGAUCUUCUCGAUCUAACAAAAAAGACAAAUCUGCCACAAA<br>GCUUGAGAGCAUCUUCGGAUGCAGAGGCGGCAGCCUUCGG<br>UGGCGCGAUAGCGCCAACGUUCUCAACUAUGACACGCAAAA<br>CGCGUGCUCCGUUGAAUGGAGUUUAUCAUG	**Method 2**<br>Ref. 21

**t1**	pGACCAAUCUGCCCUCAGAGCCCGAGAACAUCUUCGGAUGC AGAGGAGGCAGGCUUCGGUGGCGCGAUAGCGCCAACGUCC UCAACCUCCAAUGCAUCCCACCACAUGAUGAGCCUGAAGAG CCUUGGUUUUUUG	**Method 2** Ref. 21
**t5**	pGGAUCUUCUCGAUCUAACAAAAAGACAAAUCUGCCAUCAA AGCUUGAGAGCAUCUUCGGAUGCAGAGGCGGCAGCCUUCG GUGGCGCGAUAGCGCCAACGUUCUCAACCAUGACAUGCAA AACGCGUGCUUCGUUGAAUGGAGUUUUUCAUG	**Method 2** Ref. 4
**+1**	pGACCAAUCUGCCCUCAGAGCUCGAGAACAUCUUCGGAUGC AGAGGAGGCAGGCUUCGGUGGCGCGAUAGCGCCAACGUCC UCAACCUCCAAUGCAUCCCACCACAUGAUGAUGCCUGAAGA GCCUUGGUUUUUUG	**Method 2** Ref. 4
**Y**	pGGACAACCAAAAAGACAAAUCUGCCCUCAGAGCUUGAGAA CAUCUUCGGAUGCAGAGGAGGCAGCCUUCGGUGGCGCGAG AGCGCCAACGUUCUCAACAGACGCACAAUACUCCCGCUUCG GCGGGUGGGGAUAACACCUGACGAAAAGGCGAUGUUAGAC ACGCCAAGGUCAUAAUCCCCGGAGCUUCGGCUCC	**Method 2** Ref. 24
**FITCγ**	FITC-C_{18}Spacer-CGGAU	IDT
**Cy5γ**	Cy5-UUGCCAC	IDT
**FITCA**	FITC-UCAACCG	IDT
**Cy5A**	Cy5-UCACCAC	IDT
**FITCB**	FITC-AUUUACCAG	IDT
**Cy5B**	Cy5-GUCUCU	IDT
**FITCC**	FITC-AUUACG	IDT
**Cy5C**	Cy5-ACUAUC	IDT
**^{ppp}triplets**	Triplets individually prepared as in ref. 4. Equimolar amounts of each of 64 mixed to make ^{ppp}NNN	**Method 3**
**Bbio**	AAGAUGCUCUCAAGCUUUGAUGGCAACAAACAAACAAACAA ACA-biotin	IDT
**γbio**	GGACCGAAAGGUCCGCCACCGAAGGCUGCCGCCUCUGCAU CCAACAAACAC-$(PEG)_4$-biotin	IDT
**dbio**	AUGUCAUGGUUGAGAACGUUGGCGCUAUCGCAACAAACAAA CAAACA-biotin	IDT
**N_{20}**	FITC-NNNNNNNNNNNNNNNNNNNN	IDT
**IIILigBio**	P-CATAGCANNNNNNAGATCGGAAGAG-biotin	Merck
**p10IIILigup**	GGGCUGCCAACCGACCGACUAUCNNNNNNUGCUAUG CATAGCANNNNNNGATAGTCGGTCGGTTGGCAGCCCTATAG TGAGTCGTATTAATTTCGCGGGCGAGATCGATC	**Method 4** Sigma
**P5xGGGp10**	AATGATACGGCGACCACCGAGATCTACACTCTTTCCCTACAC GACGCTCTTCCGATCTNNNXXXXXXGGGCTGCCAACCG	Sigma

	XXXXXX = unique barcode	
**P3IIIrt**	CAAGCAGAAGACGGCATACGAGATCGGTCTCGGCATTCCTGCTGAACCGCTCTTCCGATCTGTGTGCTCTTCCGATCT	Sigma
**FITCrep**	FITC-GGUCCA	IDT
**Cy5rep**	Cy5-UGCUACA	IDT
**^{ppp}GUAGC**	^{ppp}GUAGC<br>GCTACTATAGTGAGTCGTATTAATTTCGCGGGCGAGATCGATC	**Method 3**<br>Sigma
**^{ppp}GGACC**	^{ppp}GGACC<br>GGTCCTATAGTGAGTCGTATTAATTTCGCGGGCGAGATCGATC	**Method 3**<br>Sigma
**^{ppp}CGGU**	^{ppp}CGGU<br>ACCGTTATAGTGAGTCGTATTAATTTCGCGGGCGAGATCGATC	**Method 3**<br>Sigma
**^{ppp}UGAAUG**	^{ppp}UGAAUG<br>CATTCAGTATAGTGAGTCGTATTAATTTCGCGGGCGAGATCGATC	**Method 3**<br>Sigma
**LibN$_{17}$**	^{p}GCUACANNNNNNNNNNNNNNNNNUGGAC<br>GTCCANNNNNNNNNNNNNNNNNTGTAGCTATAGTGAGTCGTATTAATTTCGCGGGCGAGATCGATC	**Method 5**<br>Sigma
**FITCrepseq**	FITC-biotin·dT-GACUCUUCGGAGUCUGGUCCA	IDT
**FITCγSeq**	FITC-biotin·dT-GACUCUUCGGAGUCUUCGGAU	IDT
**HDVLig**	^{p}GGGTCGGCATGGCATC-C$_3$Spacer	IDT
**HPγSeq**	GACTCTTCGGAGTCTTCGGAT	Sigma
**P5xHPγn**	AATGATACGGCGACCACCGAGATCTACACTCTTTCCCTACACGACGCTCTTCCGATCTNNNXXXXXXGACTCTTCGGAGTCTTCGGAT XXXXXX = unique barcode	IDT
**P3HDV**	CAAGCAGAAGACGGCATACGAGATCGGTCTCGGCATTCCTGCTGAACCGCTCTTCCGATCTGATGCCATGCCGACCC	IDT
**P5repseqn**	AATGATACGGCGACCACCGAGATCTACACTCTTTCCCTACACGACGCTCTTCCGATCTNNNXXXXXXGATGCCATGCCGACCC<br>XXXXXX = unique barcode	IDT
**P7repseq**	CAAGCAGAAGACGGCATACGAGATGTGACTGGAGTTCAGACGTGTGCTCTTCCGATCTNNNATCACGGACTCTTCGGAGTCTG	IDT
**Rγ1^{D}**	Duplex of Mγ^{+}m1 and Mγ^{-}m1	**Method 6**
**FQγ1^{D}**	Duplex of Mγ^{+}m1 and Mγ^{-}m1	**Method 6**
**γ^{D}**	Duplex of γ^{+} and γ^{-}	**Method 6**
**A^{D}**	Duplex of A^{+} and A^{-}	**Method 6**
**B^{D}**	Duplex of B^{+} and B^{-}	**Method 6**
**C^{D}**	Duplex of C^{+} and C^{-}	**Method 6**

Supplementary Table 3 – Custom scripts

Custom scripts supporting analysis of triplet-based replication products are available in the GitHub repository: https://github.com/JamesAttwater/RNArepseq

In scripts from ‘Sequence_lengths’ onwards, datasets 'RZ5N', 'N17L', 'N73L', 'N73H', 'F201N', 'F205N', 'FN17L', 'FN73L', and 'FN73H' correspond to samples A-I respectively (Extended Data Fig. 9).

Name	*Language*	*Description*	*Lines*	*Dependencies*
**specify**	Python 3	Generate .txt file of all possible in frame species from a template.	25	NumPy, Biopython 1.71[45]
**intermaster1.2**	Python 3	Map clipped HTS reads to list of expected species, with option to tolerate errors.	98	NumPy, Biopython 1.71[45]
**tally**	Python 3	Measures and counts sequence identity with expected synthesis product	29	
**24ntrandom**	Python 3	Generates random sequences from a defined set of triplets	31	
**Sequence_lengths**	Python 3	Prepares sequences to plot lengths of sequences (inputs from .fasta files)	59	pandas, os, Biopython
**Plot_sequence_lengths**	R	Plots lengths of sequences before filtering (inputs from Sequence_lengths.py)	50	ggplot2, tidyverse, tidyr, dplyr, RColorBrewer
**Alignments**	Python 3 (Jupyter notebook)	Aligns sequences to ribozyme sequences after filtering, assess uniqueness, complementarity and nucleotide composition (inputs from .fasta files)	1317	pandas, os, Biopython, numpy
**Family_box_codons**	Python3	Assess continuity of family box codons in pool H+I and random sequences (inputs from .fasta files)	333	pandas, os, Biopython
**Plot_alignment_count**	R	Plots of the bins of different alignments (inputs from Alignments.ipynb)	231	ggplot2, tidyverse, tidyr, dplyr, RColorBrewer

**Plot_alingment_ribozyme**	R	Plots scores of aligned sequences and their position on the ribozyme (inputs from Alignments.ipynb)	55	ggplot2, tidyverse, tidyr, dplyr, RColorBrewer
**Plot_family_box_codons**	R	Plots percentage of sequences with contiguous family box codons (input from Family_box_codons.py)	47	ggplot2, tidyverse, tidyr, dplyr, RColorBrewer
**Plot_uniqueness**	R	Plots uniqueness of sequences in different categories (input from Alignments.ipynb)	48	ggplot2, tidyverse, tidyr, dplyr, RColorBrewer
**Plot_complementarity**	R	Plots complementarity of sequences in different categories (input from Alignments.ipynb)	57	ggplot2, tidyverse, tidyr, dplyr, RColorBrewer
**Plot_identity_vs_length**	R	Plots identity of sequences with the ribozyme of different length (input from Alignments.ipynb and Intensities_HI.csv)	231	ggplot2, tidyverse, tidyr, dplyr, RColorBrewer, plyr
**Plot_nucleotide_composition**	R	Plots nucleotide composition of triplets over multiple cycles in non-ribozyme sequences (input from Alignments.ipynb)	72	ggplot2, tidyverse, tidyr, dplyr, RColorBrewer

Supplementary Methods

RNA preparation

All nucleic acid sequences are listed in Supplementary Table 2.

Method 1: The indicated DNA oligonucleotide and oligonucleotide 'HDVrt' were mutually extended upon one another using three thermocycles with GoTaq Hot-start DNA polymerase (Promega). This generated a double-stranded DNA comprising the DNA sequence of the desired RNA oligonucleotide, downstream of a T7 promoter sequence, but upstream of the DNA sequence of the HDV ribozyme. This DNA was QIAQuick column purified (Qiagen) and transcribed in the presence of guanosine monophosphate (15 ng/μl DNA, 20 mM $MgCl_2$, 50 mM Tris·HCl pH 7.9, 10 mM DTT, 2 mM spermidine, 6.25 mM of each of the four NTPs, 0.01 U/μl inorganic pyrophosphatase (Thermo Scientific), 20 ng/μl T7 RNA polymerase). During overnight transcription at 37°C, co-transcribed HDV ribozyme cleaved itself off from the RNA 3' end to yield uniform 3' termini; adding urea to 6 M and incubating for a further hour at 37°C enhanced this cleavage[46]. After PAGE purification the RNA was treated with polynucleotide kinase (NEB) to remove the residual 2', 3'-cyclic phosphate before acid phenol/chloroform extraction to remove enzyme and ethanol precipitation to yield the final oligonucleotide.

Method 2: Ribozymes were prepared as per method 1 above, but the DNA transcription template (with the ribozyme-encoding sequence between a T7 promoter and HDV ribozyme) was generated by PCR. Transcription included excess guanosine monophosphate (GMP, 10 mM, with only 2 mM of each of the four NTPs) to yield 5' monophosphorylated RNAs that avoid participation as substrates in ribozyme-catalysed ligation.

Method 3: Triphosphorylated triplets and oligomers were prepared as in ref. 4. Briefly, DNA oligonucleotide '5T7' and the relevant templating DNA were mixed in transcription buffer at 1.5 μM to give a double-stranded T7 promoter with a downstream 5' overhang sequence encoding the desired oligonucleotide (see ref. 4 for details of overhang choice for each triplet). The only nucleotides added to the transcription buffer were 2.4 mM of NTP for each nucleotide present in the desired product. The correct triplet product was then purified from a 30% polyacrylamide 3M urea gel and, after precipitation in 85% ethanol, its concentration was determined by UV absorbance and extinction coefficients calculated using OligoCalc[44]. ^{ppp}NNN comprises an equimolar mix of the 64 possible triplet sequences.

Method 4: RNA adapter for RNA sequencing was transcribed as in method 1, but from DNA generated by mutual extension with oligonucleotide '5T7' instead of 'HDVrt'. As a result there was no HDV cleavage step in transcription. After PAGE purification, the RNA was treated with alkaline phosphatase (rSAP, NEB) instead of PNK to remove 5' phosphorylation and prevent adapter concatenation during adapter ligation.

Method 5: RNA library was prepared as in method 4, but included GMP in transcription as in method 2, and omitted any alkaline phosphatase treatment after PAGE purification.

Method 6: RNA duplex preparation. All RNA duplexes were prepared by native PAGE purification. 600 pmol of each complementary RNA were annealed in 60µl of 0.1 M NaCl, 5 mM tris·HCl pH 7.4 (80°C 1 s, 0.1°C/s to 4°C, kept on ice until loading with glycerol added to 12%). After 0.5× TBE 20% PAGE separation (run at 10 W for 1 h) and excision of the duplex band (identified by comparison to single-strand controls), duplex was eluted into 0.1 M NaCl, 2 mM tris·HCl pH 7.4 overnight at 4°C. After removal of gel fragments by passage through a Spin-X 0.22 µm cellulose acetate filter (Costar), salts eluted from the gel were removed by applying the eluate to a 3 kDa molecular weight cut-off filter (Amicon Ultra-0.5) and filtering three times, diluting the concentrate with 0.1 M NaCl, 2 mM tris·HCl before each time. The final concentrate was supplemented with 0.1% Tween-20 and stored in a DNA LoBind microfuge tube (Eppendorf) in the fridge, where it showed no evidence of separation after > 6 months storage. Strand separation of duplexes was measured in a fluorescence/quench assay as described in Extended Data Fig. 2.

RNA sequencing

Sequencing of primerless fragment syntheses

To understand the synthesis pathways of ribozyme fragments (Extended Data Fig. 4), polymerisation products made on templates without primers by the $t5^{+1}$ TPR[4] were eluted and sequenced (Scheme in Supplementary Fig. 4). Reaction profiling was carried out on ribozyme reactions set up with 4 pmol of biotinylated template (Bbio, γbio or dbio encoding the β^+, γ^+ or δ^+ fragments[4] of t5 respectively) prebound to 40 µg MyOne C1 microbeads, 2 pmol of $t5^{+1}$, and 50 pmol of each target fragment's constitutive triplet in 10 µl of 2× extension buffer, supplemented with 0.15% Tween-20. Reactions were frozen at -7°C and stopped after 4 (Bbio, γbio) or 11 (dbio) days by vortexing until thawed, whereafter all steps were carried out on ice to maximise the retention of any short products hybridised to templates.

Beads were twice washed with ice cold BWBTMg (200 mM NaCl, 10 mM tris·HCl pH 7.4, 20 mM $MgCl_2$, 0.1% Tween-20) to remove substrates and ribozyme whilst maintaining duplex annealing, changing to a new pre-chilled tube in between. Beads were then resuspended in 5 µl 10 mM EDTA, 0.1% Tween-20, before addition of 5 µl 80 mM NaOH to elute products from templates. This supernatant was removed after 1 min and neutralised with a mix of 0.4 µl 1 M tris·HCl pH 7.4 and 0.4 µl 1 M HCl.

Sequence analysis began with 3' adaptor ligation: 2.7 µl of the sample of eluted extension products were included in a 10 µl T4 RNA ligase 2 truncated KQ reaction (1× T4 RNA ligase buffer (NEB), 2 µM IllLigBio adapter pre-adenylated using a 5' adenylation kit (NEB), 20-200 nM ^{ppp}UGAAUG hexamer standard, 15% PEG, 20 U/µl of T4 RNA ligase 2 truncated KQ (NEB), 10°C overnight). RNA-adapter ligation products were purified by denaturing PAGE, then eluted and ethanol precipitated with a glycogen carrier as described above. After resuspension in 5 µl water, 5' triphosphates on the synthesis products were converted into 5' monophosphates: samples were treated with recombinant shrimp alkaline phosphatase (NEB) (10 µl reaction in 1× NEB buffer 2.1, 37°C 30 min, 65°C 5 min), these reactions were made up to 15 µl including 0.5 µl 10× NEB 2.1, 1.5 µl 10 mM ATP, 0.75 µl 0.1 M DTT and 0.3 U/µl T4 Polynucleotide kinase (NEB), and then incubated at 37°C 30 min, 65°C 20 min.

After addition of a monophosphate, 6 µl aliquots were 5' adapter ligated (25 µl of 1× T4 RNA ligase buffer (NEB), 1 mM ATP, 0.4 µM p10IIIligup adaptor, 25% PEG, 0.5 U/µl T4 RNA ligase 1, 25°C 2 hr, 16°C overnight). Adapters contained random sequence stretches and a short mutually-complementary region to maximise ligation efficiency and generality[43]. 2.5 µl aliquots were subsequently reverse transcribed and PCR amplified in a 50 µl SuperScript III one-step RT-PCR System (Invitrogen), using 0.4 µM barcoded P5XGGGP10 and P3IIIrt primers. PCR products were agarose gel purified using a Monarch gel extraction kit (NEB) and sequenced on an Illumina HiSeq.

Using the Galaxy web platform at the public server usegalaxy.org[47], the first three positions (of random sequence) and any positions beyond 120 nt were trimmed off the sequencing reads, before quality filtering (requiring >90% of positions with >Q=25) and conversion to .fasta format. Sequences possessing AGATCGGAAGAG from the 3' adapter were then retained. This motif, the preceding 13 positions and all downstream positions were then trimmed off to remove the 3' adapter sequence before the data was split by the identity of the first six 'barcode' positions of each sequence, also requiring an intact 5' adapter sequence: XXXXXXGGGCTGCCAACCGACCGACTATC. The first 42 positions of each sequence were then trimmed to fully remove the 5' adapter, yielding raw data files of insert sequences ('Btri4_GRC.fasta', 'ytri4_GRC.fasta', 'dtri11_GRC.fasta'), with 130,000 – 780,000 reads per test sample. In parallel negative control reactions were set up without triplets, and processed and analysed similarly ('Bneg_GRC.fasta', 'yneg_GRC.fasta', 'dneg_GRC.fasta').

Using custom script 'intermaster1.2' (Supplementary Table 3), reads were then mapped against a list of all possible species for their respective template generated using custom script 'specify' (Supplementary Table 3), allowing for one mismatch in the three 5' and three 3' positions, or one mismatch anywhere for hexamer reads. This produced counts of each oligonucleotide product ('counts_compiled_2.2.xlsx') which were then normalised by the count of the spiked-in ^{ppp}UGAAUG hexamer, reduced by reads from negative control samples (comprising 0.03 - 16% of the number of sample reads), and converted to fmol or amol values of each product made per pmol of template, assuming a linear scaling of reads with concentration. These data were used to generate the fragment synthesis pathways as described in Extended Data Fig. 5.

Sequencing of N_{17} library replication products

The reactions in Fig. 3b were continued up to 55 cycles. Then, after 3-fold serial dilution as described (Fig. 3a), a final cycle was performed with 60 nM final concentration of FITCrepseq primer replacing added FITCrep, and incubated frozen for 19 days at -7°C. The FITCrepseq primers, extended upon replicated templates in the reaction, were then recovered via a biotin moiety on the primer (see 'denaturing gel electrophoresis' above) and gel-purified by length as described in Supplementary Table 1. They were eluted from the gel fragments back onto fresh microbeads, which were passed through a 50 µm filter (CellTrics), washed and ligated to a 3' adapter (1× T4 RNA ligase buffer (NEB), 10% PEG-8000, 10 U/µl T4 RNA Ligase 2 truncated KQ (NEB), 2 µM HDVLig (pre-adenylated using a 5' adenylation kit (NEB)), 10°C overnight, 65°C 10 minutes). The beads were washed again and resuspended in a SuperScript III/Platinum Taq rtPCR reaction (Invitrogen) with HPrepseq and HDVrec

primers, which was spiked at 5% into PCR with P7repseq and P5repseqn primers to convert into a sequencing construct. These PCR products were agarose gel purified using a Monarch gel extraction kit (NEB) and sequenced on an Illumina MiSeq. Reads were quality filtered and trimmed using BBtools (https://jgi.doe.gov/data-and-tools/software-tools/bbtools/), demultiplexed using a custom python script (https://github.com/holliger-lab/fidelity-analysis/blob/master/fasta_demux.py), filtered for replication competent sequences (containing both primer binding sites), and screened as described in Supplementary Table 1.

Sequencing of γ fragment replication products

γ^+ synthesis products were purified by PAGE as described in Extended Data Fig. 8 and Supplementary Fig. 7. After 85% ethanol precipitation with glycogen carrier, these products were resuspended in water and ligated to an HDVLig 3' adapter as described above. This ligation was spiked at 2% into a SuperScript III/Platinum Taq rtPCR reaction (Invitrogen) with HPγseq and HDVrt primers. To add sequencing tags, this reaction was spiked at 1% into a GoTaq HotStart (Promega) PCR reaction with primers P3HDV and P5XHPγn, and the desired product was agarose gel purified using a Monarch gel extraction kit (NEB) and sequenced on an Illumina MiSeq.

The Galaxy web platform[47] was used for initial processing of reads as described above, with sequences possessing GGGTCGGCATGGCATC from the 3' adapter retained, before this motif and all downstream positions were trimmed off. The data was split by the sequence: XXXXXXGACTCTTCGGAGTCTTCGGAT containing a barcode region and the FITCγSeq primer sequence, yielding raw data files of extended primers ('1minus.fasta', '1yD.fasta', '5minus.fasta', '5yd.fasta'), with 59,000 – 74,000 reads per test sample. The first 27 positions of each sequence were then trimmed to fully remove the 5' primer sequence, and any extension products shorter than full-length (24 nt) were discarded, yielding full-length extension product data files ('1minusFL+.fasta', '1yDFL+.fasta', '5minusFL+.fasta', '5ydFL+.fasta'). The degree of identity of 24-nt sequences with the target sequence GCAGAGGCGGCAGCCTTCGGTGGC in each sample was counted by custom script 'tally' (Supplementary Table 3), and these distributions were compared to the identity distribution of sequences generated from a random mix of the substrate triplets present (built and analysed using sequences generated by custom script '24ntrandom' (Supplementary Table 3)), as detailed in source data file 'Error tallies weighted.xlsx' and shown in Supplementary Fig. 7c.

Sequencing of triphosphorylated amplification products

To identify TPR-synthesised RNA products from cycling in the absence of primers with ^{ppp}NNN, 3' adapters were ligated to RNAs purified from the gel in Fig. 5b (1× T4 RNA ligase buffer (NEB), 1.6 µM IllLigBio adapter pre-adenylated using a 5' adenylation kit (NEB), 15% PEG, 16 U/µl of T4 RNA ligase 2 truncated KQ (NEB), 10°C 20 h, 65°C 12 min). The reaction was then supplemented with 40 mM tris·HCl pH 8.3 and 0.5 U/µl RppH (NEB) before incubation (37°C 80 min) to convert 5' triphosphates to 5' monophosphates before purification of ligated products by denaturing PAGE. These were eluted and precipitated before ligation to 5' adapters (1× T4 RNA ligase buffer (NEB), 0.5 µM p10IllLigup adapter, 25% PEG-8000, 1 mM ATP, 2 U/µl T4 RNA ligase 1, 25°C 2 hr, 16°C 9 h, 65°C 15 min). This

ligation was spiked at 10% into a SuperScript III/Platinum Taq rtPCR reaction (Invitrogen) with 0.4 µM P3Illrt and P5XGGGP10 primers, and the desired products were agarose gel purified using a Monarch gel extraction kit (NEB) and sequenced on an Illumina HiSeq.

Sequences were processed using the FASTX Toolkit[47] version 0.0.13. The first three positions (of random sequence) were trimmed off the sequencing reads, before quality filtering (requiring >95% of positions with >Q=30) and conversion to .fasta format. Sequences possessing AGATCGGAAGAG from the 3' adapter were then retained. This motif, the preceding 13 positions and all downstream positions were then trimmed off to remove the 3' adapter sequences. The data was split by the identity of the first six 'barcode' positions of each sequence attached to an intact 5' adapter sequence: XXXXXXGGGCTGCCAACCGACCGACTATC. The first 42 positions of each sequence were then trimmed to fully remove the 5' adapter, yielding raw data files of insert sequences ('RZ5N.fasta', 'N17L.fasta', 'N73L.fasta', 'N73H.fasta', 'F201N.fasta', 'F205N.fasta', 'FN17L.fasta', 'FN73L.fasta', and 'FN73H.fasta', corresponding to samples A-I respectively), with 39,000-640,000 reads per sample.

Sequence_lengths.py followed by Plot_sequence_lengths.R were used to plot sequenced product length distributions (Extended Data Fig. 9b). Then custom code in Alignments.ipynb was used to exclude non-triplet register products (Extended Data Fig. 9c) and products > 3 nt outside the size range excised from the gel as in Extended Data Fig. 9a. To classify product sequences by alignment to ribozyme (+) and (-) sequences (Extended Data Fig. 9d), the Biopython local alignment tool was used (pairwise2.align.localms) as part of custom code Alignments.ipynb. The gap penalty was set to -1000, and a score of 1 was given to each matching nucleotide and 0 for every mismatch. Dividing the score by the length of the given sequence yielded the fractional identity with the ribozyme, and site and degree of maximal fractional identity was recorded. Sequences above a fractional identity threshold (Extended Data Fig. 9c) were classified as 'aligned'. This script was adapted to only align the 4th-12th nt of each product to perform length-independent classification in Extended Data Fig. 9e (Alignments.ipynb & Plot_alignment_count.R).

To generate the map of alignment sites in Fig. 5h (Plot_alignment_ribozyme.R), a score was given to positions in the ribozyme sequence for each 'aligned' product. The score was corrected for the yield of different reactions, taking into account the intensity of product lanes on the gel (see source data file 'SD_ExtendedData_Fig9d.xlsx') and the total number of sequences that went into the alignment in each pool. Furthermore, if a product aligned to more than one of the four possible ribozyme sequences (5TU, t1 and complements), the score increase was shared between the relevant sites to avoid introducing bias at regions of high sequence similarity between 5TU and t1.

Custom scripts (Alignments.ipynb / Family_box_codons.py / Length_vs_identity.py) were used to generate randomised pools of sequences for control analyses in Supplementary Fig. 8 / Fig. 5d / Fig. 5e and plotted using Plot_alignment_count.R / Plot_family_box_codons.R / Plot_length_vs_identity.R, respectively. For Fig. 5e and Supplementary Fig. 8 the sequences were randomised having the same triplet composition and length distribution as the comparison pools, while for Fig. 5d, only the length distribution was maintained and otherwise completely random sequences were chosen. Custom script

(Family_box_codons.py and Plot_family_box_codons.R) was used to count contiguous family box triplets in product sequences for Fig. 5d. Length_vs_identity.py allows counting the maximum identity of a product sequence upon alignment with the ribozyme, and comparison to its total length, which is plotted with Plot_length_vs_identity.R (Fig. 5e and Supplementary Fig. 8).

The uniqueness of product sequences was assessed with custom script Uniqueness.py and plotted with Plot_uniqueness.R (Supplementary Fig. 9). Complementarity of sequences (Supplementary Fig. 9) was analysed with Complementarity.py and plotted with Plot_complementarity.R. The nucleotide composition of products (Fig. 5g) was plotted with Plot_nucleotide_triplets.R with files coming from Alignments.ipynb.

Supplementary References

43. Fuchs, R.T., Sun, Z., Zhuang, F. & Robb, G.B. Bias in Ligation-Based Small RNA Sequencing Library Construction Is Determined by Adaptor and RNA Structure. *PLOS ONE* **10**, e0126049 (2015).

44. Kibbe, W.A. OligoCalc: an online oligonucleotide properties calculator. *Nucleic Acids Res.* **35**, W43-6 (2007).

45. Cock, P.J. *et al.* Biopython: freely available Python tools for computational molecular biology and bioinformatics. *Bioinformatics* **25**, 1422-3 (2009).

46. Rosenstein, S.P. & Been, M.D. Self-cleavage of hepatitis delta virus genomic strand RNA is enhanced under partially denaturing conditions. *Biochemistry* **29**, 8011-8016 (1990).

47. Afgan, E. *et al.* The Galaxy platform for accessible, reproducible and collaborative biomedical analyses: 2016 update. *Nucleic Acids Res.* **44**, W3-W10 (2016).

Source Data for Supplementary Fig. 1

	Fluorescence (arbitrary units)					
	Chloroacetate	Chloroacetate	Phosphate	Phosphate	Formate	Formate
pH	Na^+	Mg^{2+}	Na^+	Mg^{2+}	Na^+	Mg^{2+}
1.75	47514	35277	49416	43996	42985	33304
1.9	43740	36597	48917	46860	40135	35595
2	48288	36557	48024	47710	42938	34078
2.1	47569	36601	47629	49689	43826	33948
2.2	47547	40111	46289	52619	42887	29294
2.3	48460	44779	45815	53375	40746	26344
2.4	48768	44187	48924	53820	43248	22932
2.5	48799	35210	49761	54471	44211	15900
2.6	49317	24066	48281	45964	42139	10456
2.7	50772	14956	46249	33291	41658	8741
2.8	47191	9577	47573	18355	39641	6505
2.85	42655	8304	43567	13313	35937	5847
2.9	37806	7132	39842	10768	33888	5492
2.95	31648	6789	33849	8841	28145	5302
3	23551	6231	26944	7333	21000	4917
3.05	18433	6202	18108	6436	16305	5013
3.1	14846	5949	16087	6210	13919	4961
3.15	12130	5761	12650	6670	10986	4758
3.2	10044	5704	9996	5511	9682	4705
3.25	10479	5706	8996	5394	7822	4689
3.3	7378	5492	6901	5099	13149	4645
3.35	6768	5343	6336	5076	5733	4612
3.4	6902	5523	5854	5372	5461	4530
3.5	6267	5112	5909	5137	4990	4358
3.6	6078	5036	5708	4925	6218	4361

Source Data for Supplementary Fig. 2

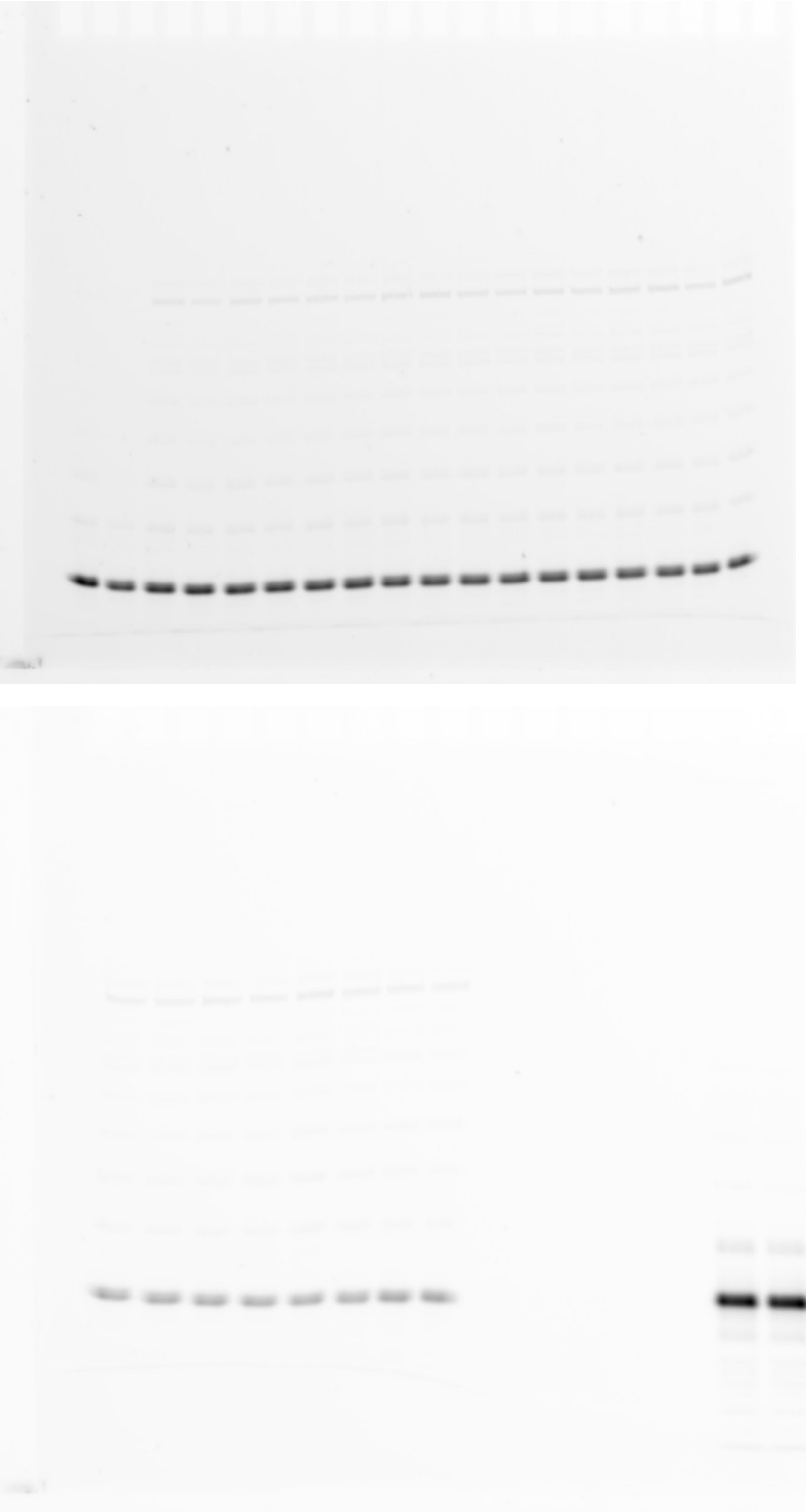

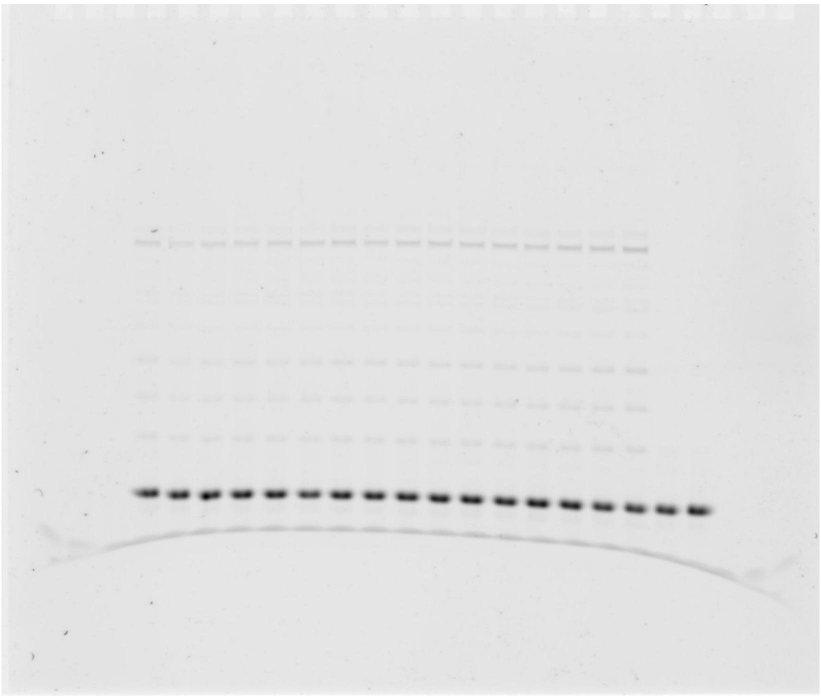

Source Data for Supplementary Fig. 3

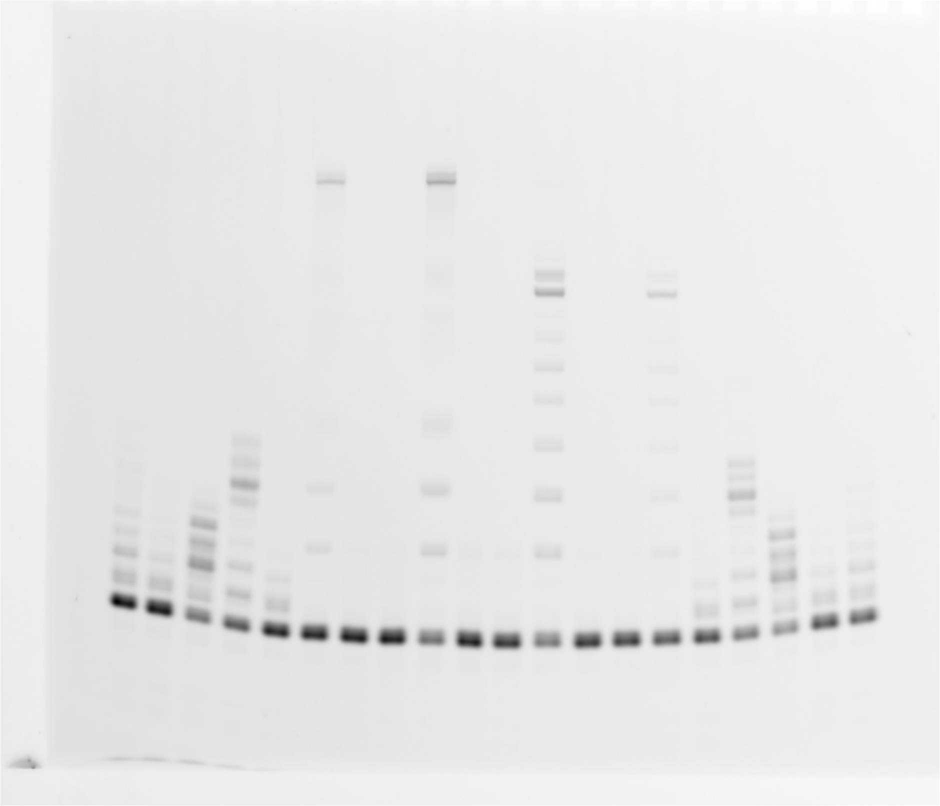

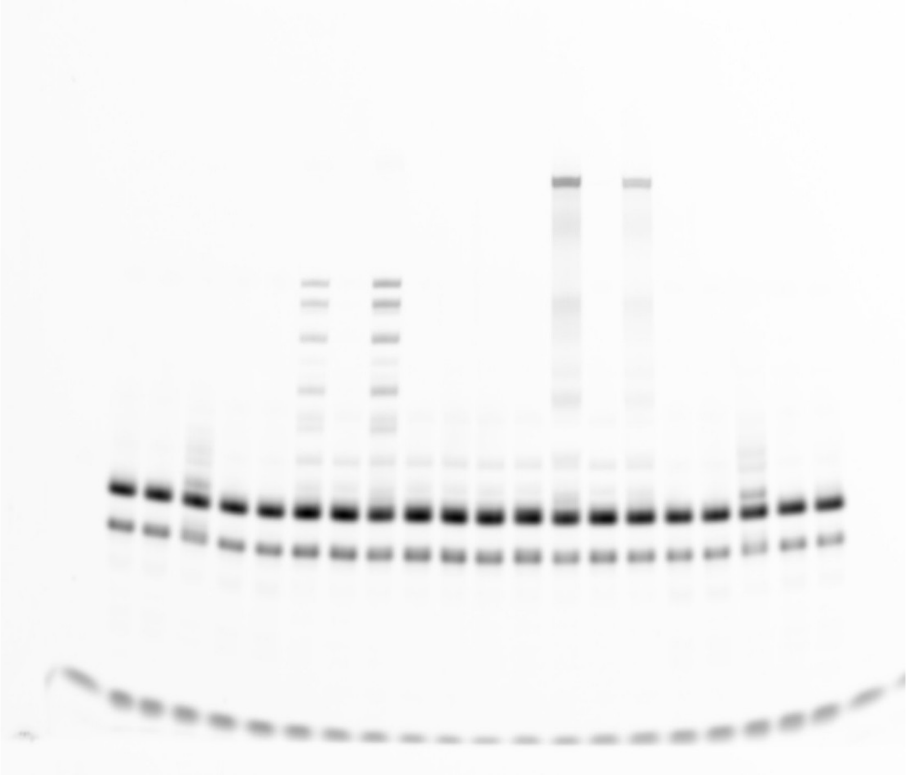

Source Data for Supplementary Fig. 5

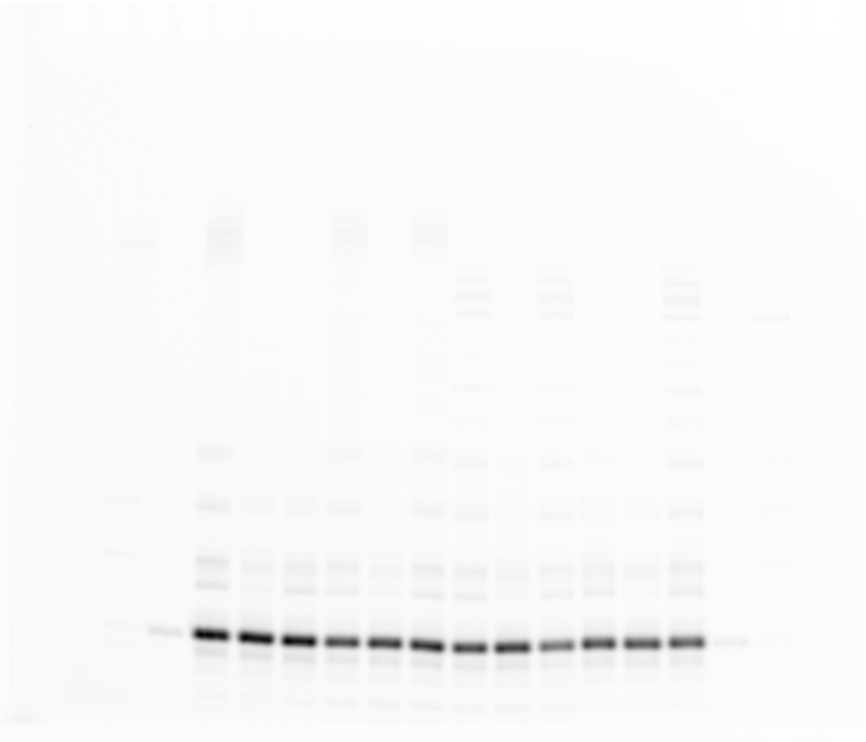

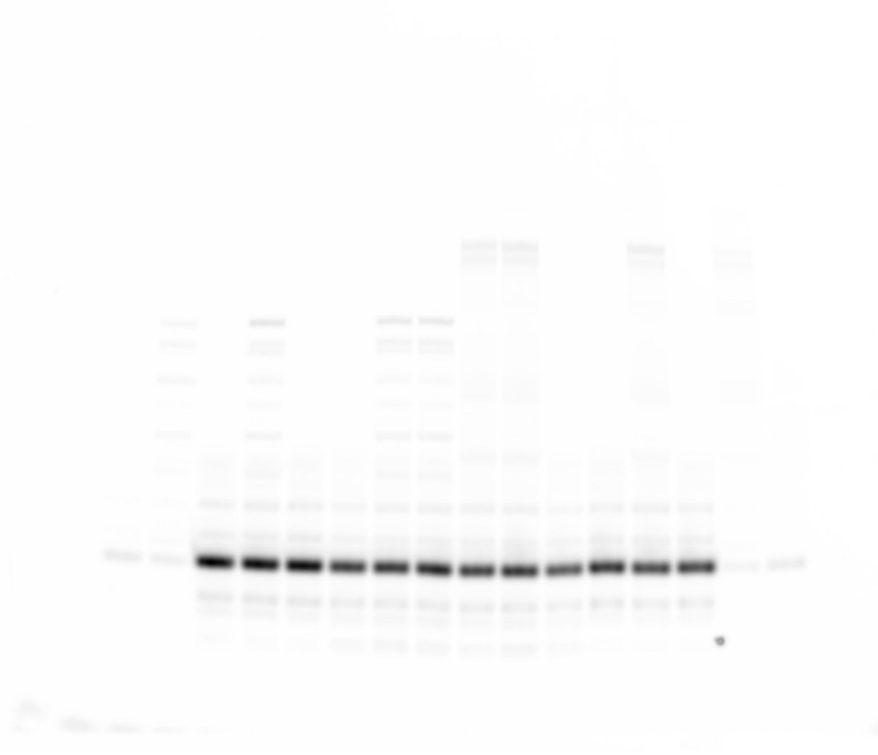

Source Data for Supplementary Fig. 6

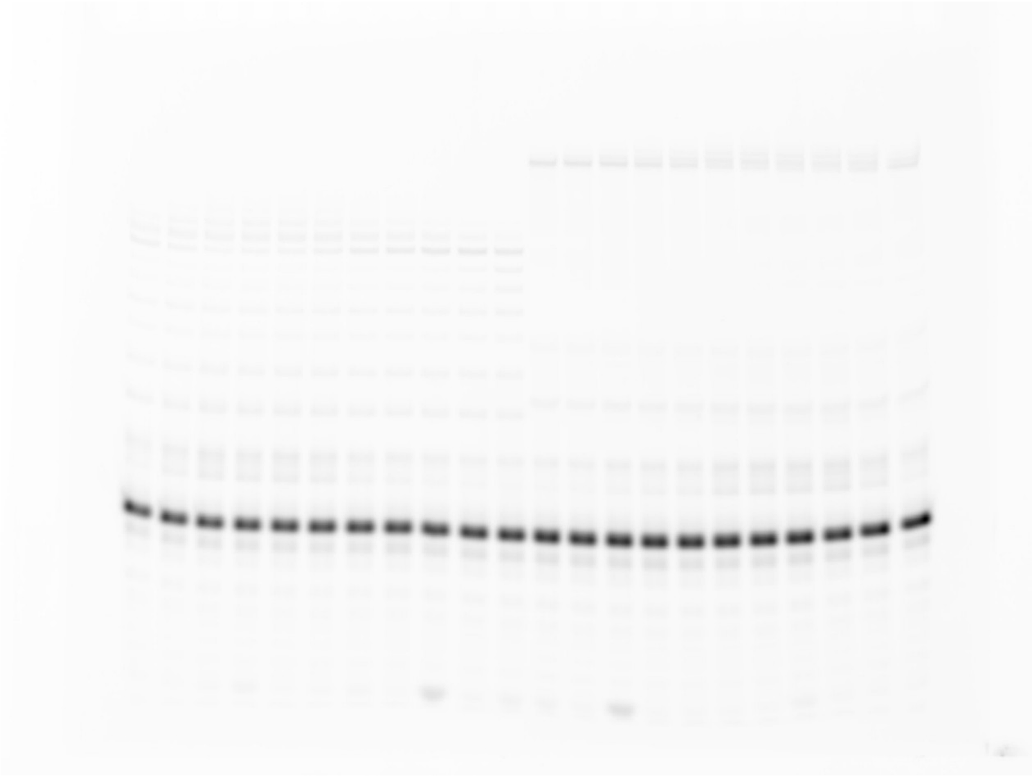

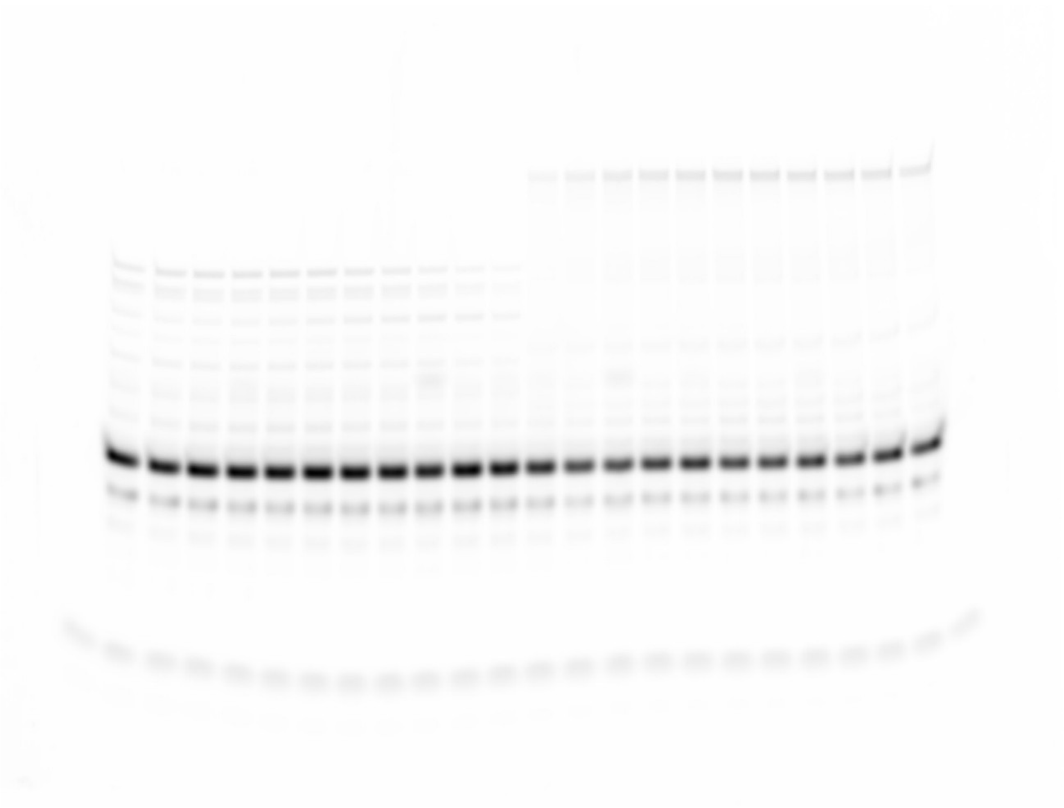

Source Data for Supplementary Fig. 7

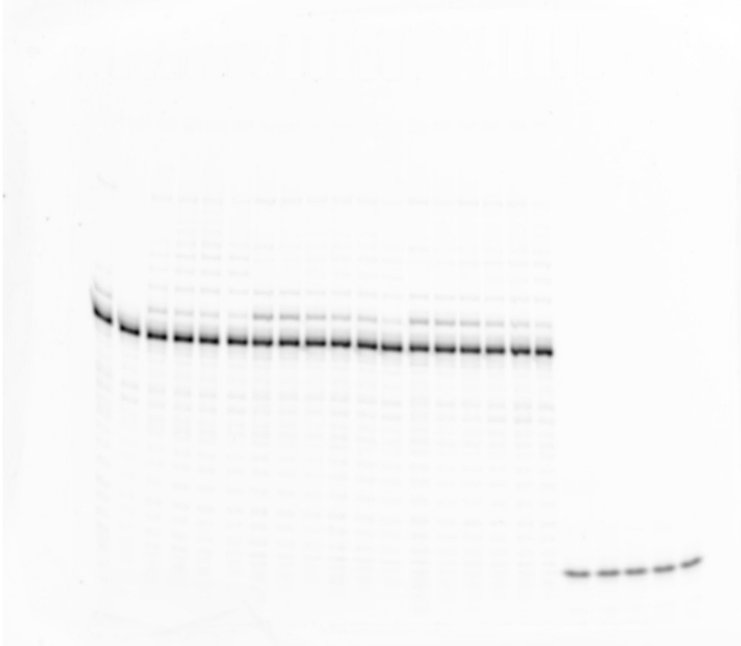

Sample distributions (%)

**Identity:**	5minus	1minus	5yD	1yD	RANDOM
**0**	0.0	0.0	0.0	0.0	0.0
**1**	0.0	0.0	0.0	0.0	0.1
**2**	0.1	0.0	0.0	0.0	0.6
**3**	0.1	0.0	0.1	0.0	1.8
**4**	0.2	0.0	0.1	0.0	5.4
**5**	0.5	0.0	0.2	0.1	9.4
**6**	0.9	5.8	0.4	0.1	13.4
**7**	2.4	23.1	2.2	0.6	16.8
**8**	1.9	17.3	1.3	0.6	15.7
**9**	2.9	21.2	1.7	0.7	13.9
**10**	3.9	1.9	2.3	0.1	9.8
**11**	5.6	5.8	3.2	0.1	6.2
**12**	6.6	0.0	4.6	0.1	3.8
**13**	6.5	0.0	5.8	0.1	1.6
**14**	8.7	1.9	8.8	0.1	0.9
**15**	9.6	1.9	10.0	0.1	0.4
**16**	7.1	0.0	7.5	0.1	0.2
**17**	2.6	0.0	2.4	0.0	0.0
**18**	2.2	0.0	1.8	0.0	0.0
**19**	3.3	0.0	2.6	0.0	0.0
**20**	3.4	1.9	2.4	0.1	0.0
**21**	2.8	3.8	1.6	0.4	0.0
**22**	2.1	1.9	2.3	5.4	0.0
**23**	12.3	3.8	18.5	48.4	0.0
**24**	14.3	9.6	20.1	43.1	0.0
