# Supplementary figures and images for: Trinucleotide substrates under pH–freeze–thaw cycles enable open-ended exponential RNA replication by a polymerase ribozyme
Source: Nat Chem. 2025 May 28;17(7):1129–37. doi: 10.1038/s41557-025-01830-y (PMC12226338; doi:10.1038/s41557-025-01830-y)

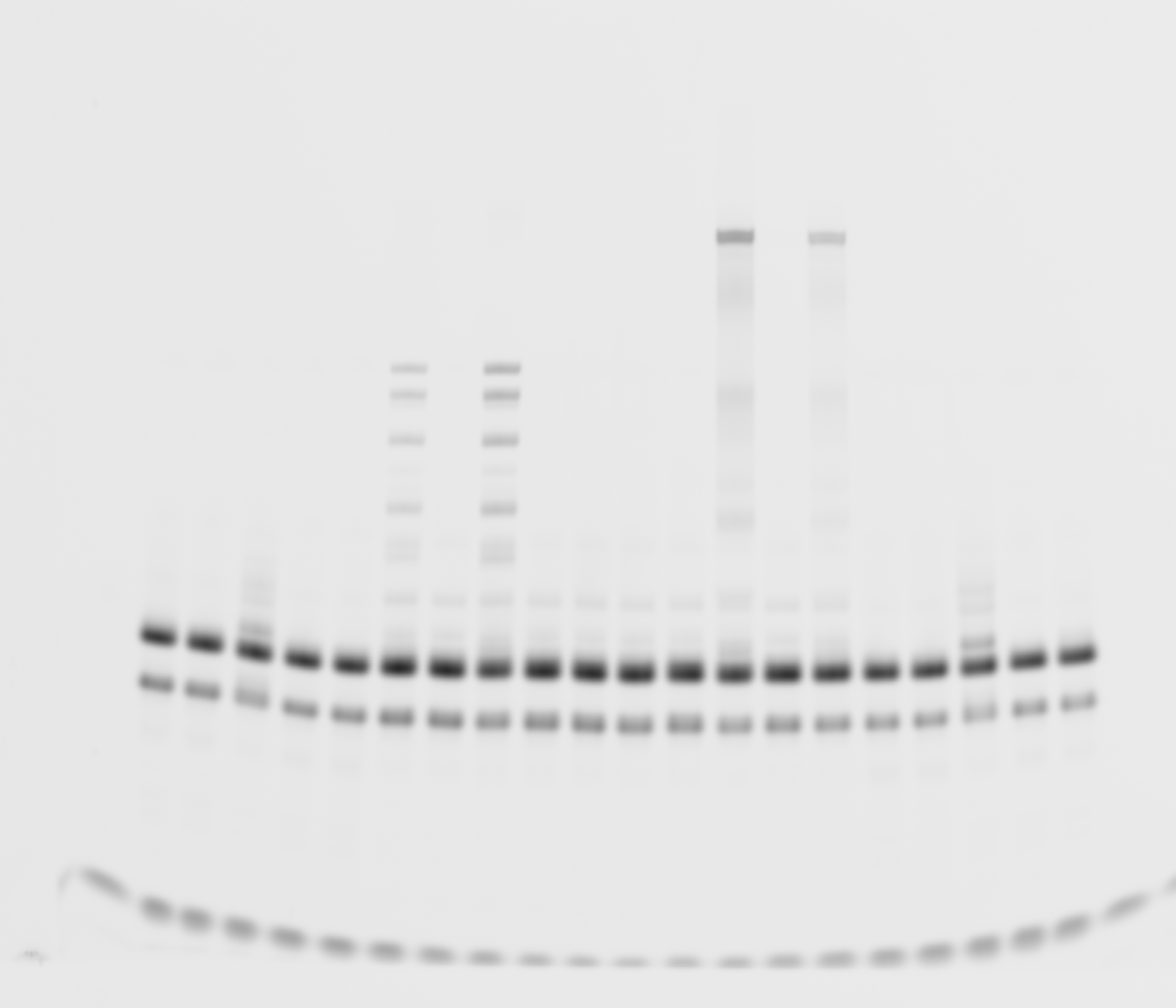

Supplement: Supplementary file 2 — Uncropped gels for Fig. 1c and graphed values for Fig. 1d. [file 41557_2025_1830_MOESM2_ESM.zip › SD_Fig1/SD_Fig1c_lower.tif]

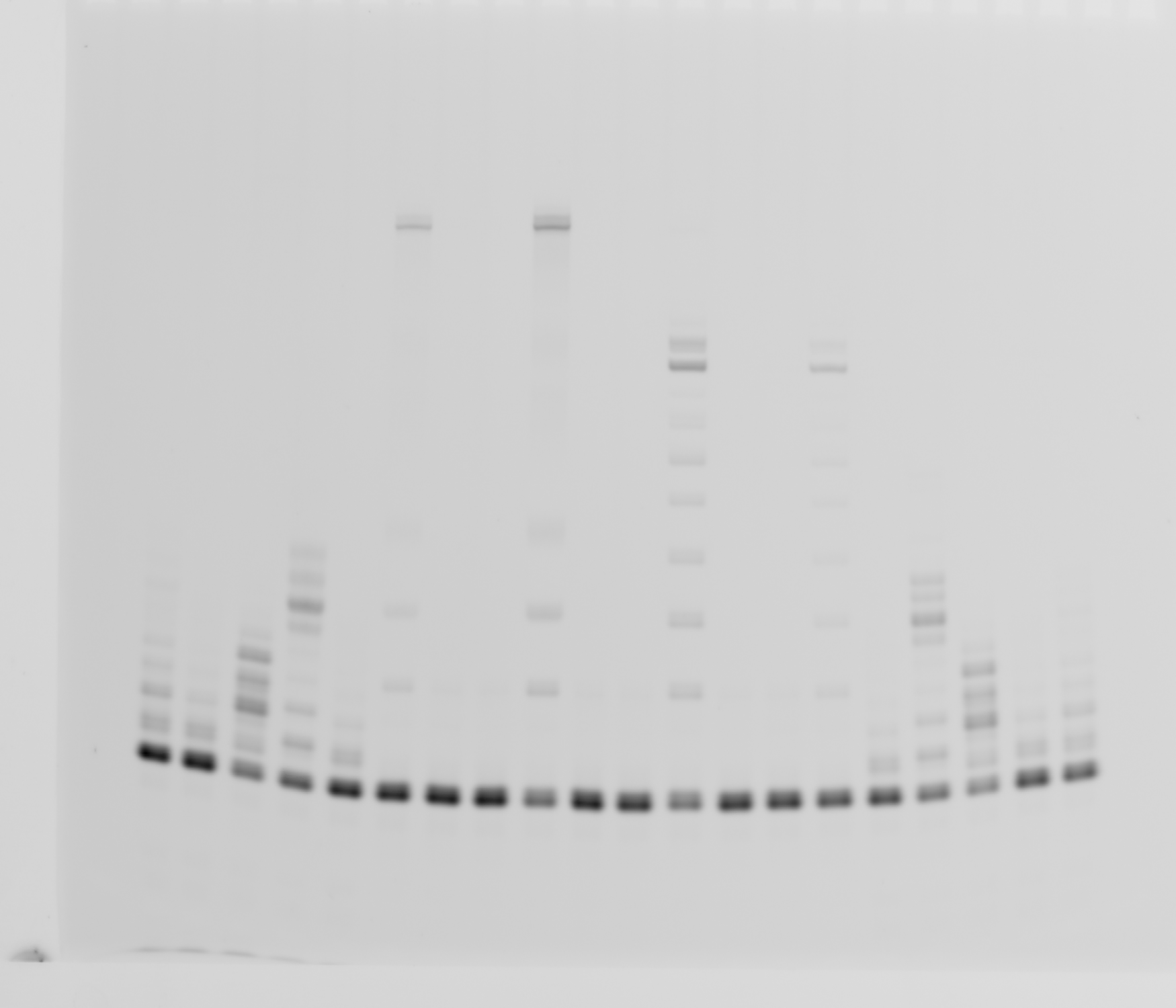

Supplement: Supplementary file 2 — Uncropped gels for Fig. 1c and graphed values for Fig. 1d. [file 41557_2025_1830_MOESM2_ESM.zip › SD_Fig1/SD_Fig1c_upper.tif]

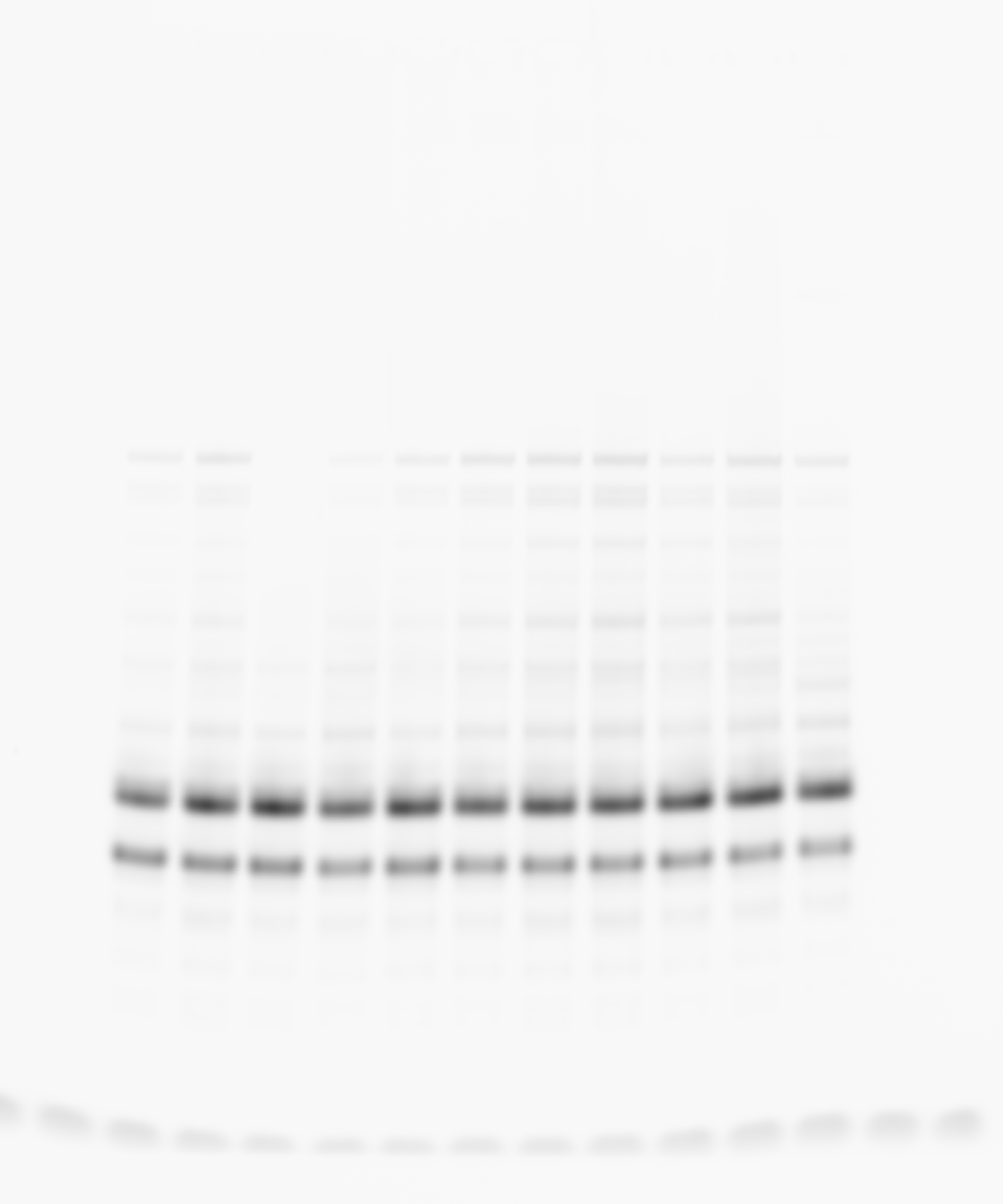

Supplement: Supplementary file 3 — Uncropped gels for Fig. 2c. [file 41557_2025_1830_MOESM3_ESM.zip › SD_Fig2/SD_Fig2c_lower.tif]

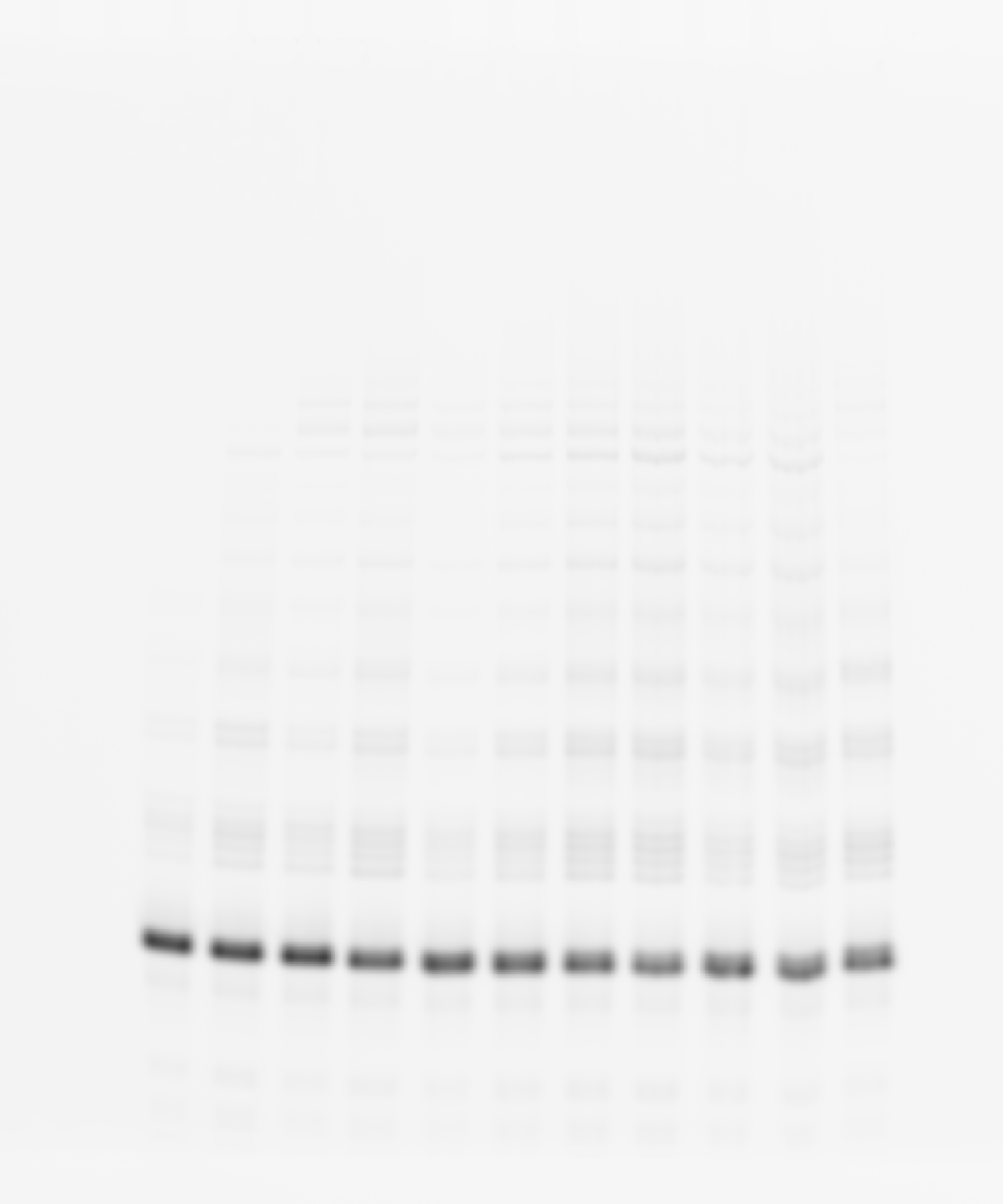

Supplement: Supplementary file 3 — Uncropped gels for Fig. 2c. [file 41557_2025_1830_MOESM3_ESM.zip › SD_Fig2/SD_Fig2c_upper.tif]

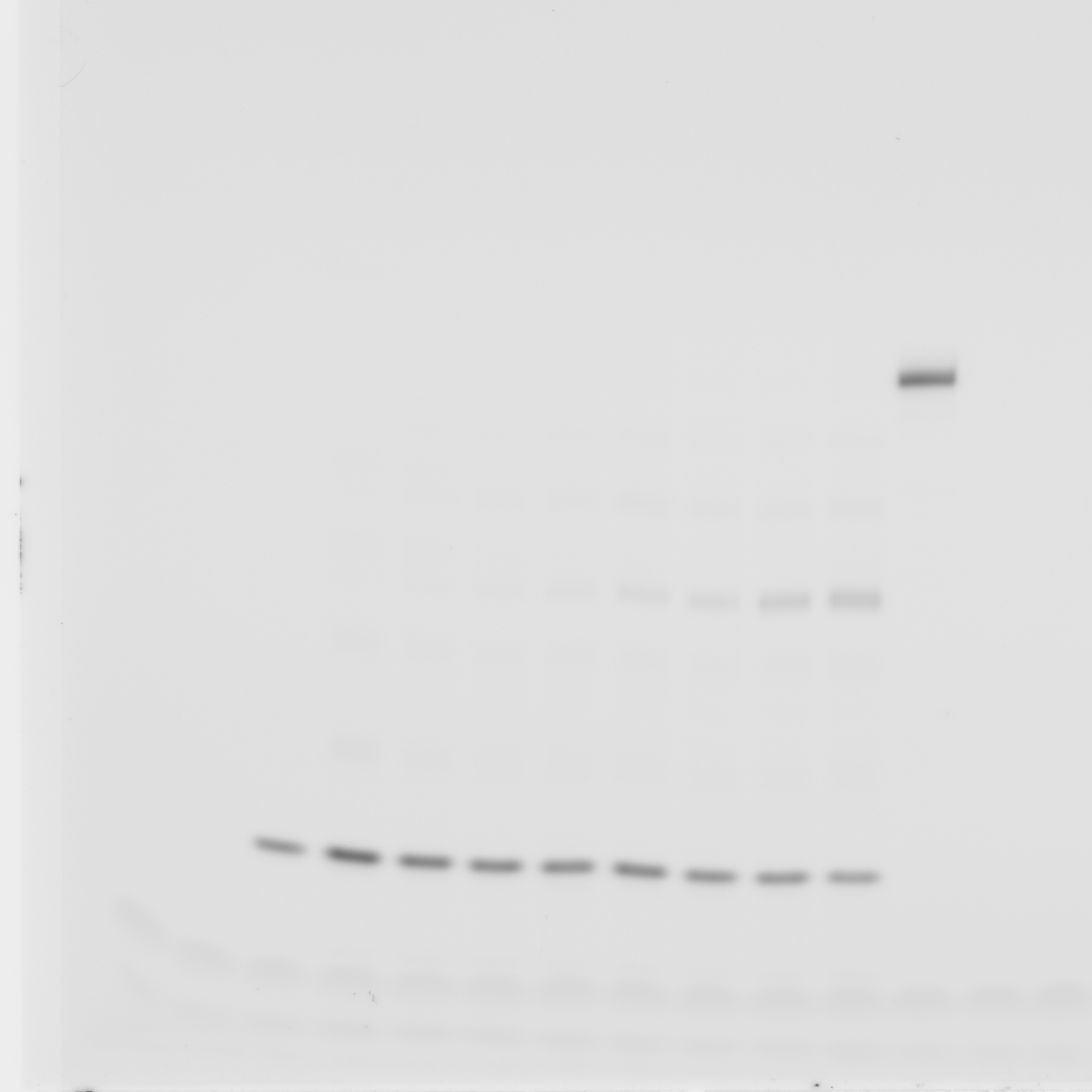

Supplement: Supplementary file 4 — Uncropped gels for Fig. 3b and 3d and graphed values for Fig. 3c and 3e. [file 41557_2025_1830_MOESM4_ESM.zip › SD_Fig3/SD_Fig3b.tif]

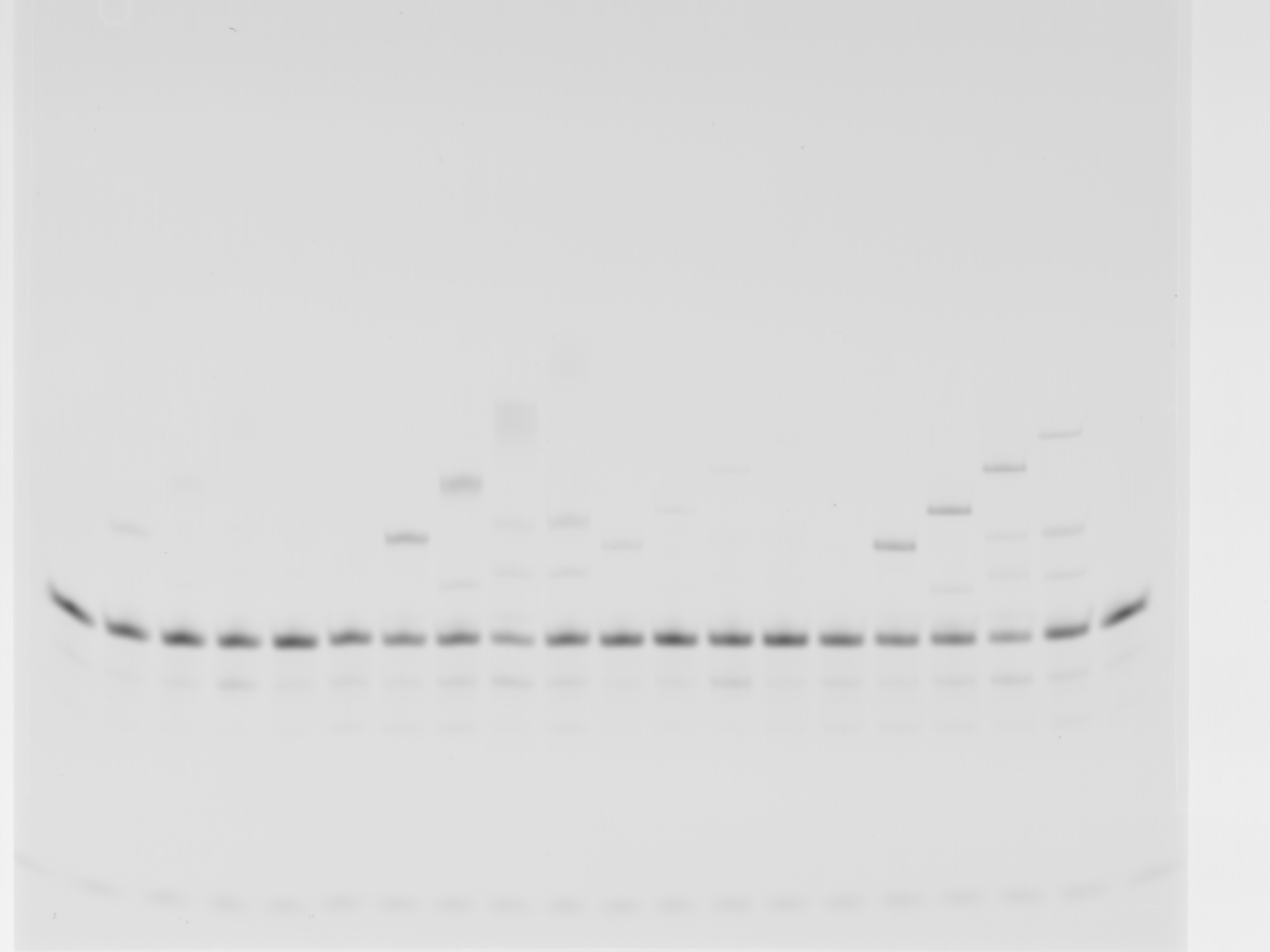

Supplement: Supplementary file 4 — Uncropped gels for Fig. 3b and 3d and graphed values for Fig. 3c and 3e. [file 41557_2025_1830_MOESM4_ESM.zip › SD_Fig3/SD_Fig3d_lower.tif]

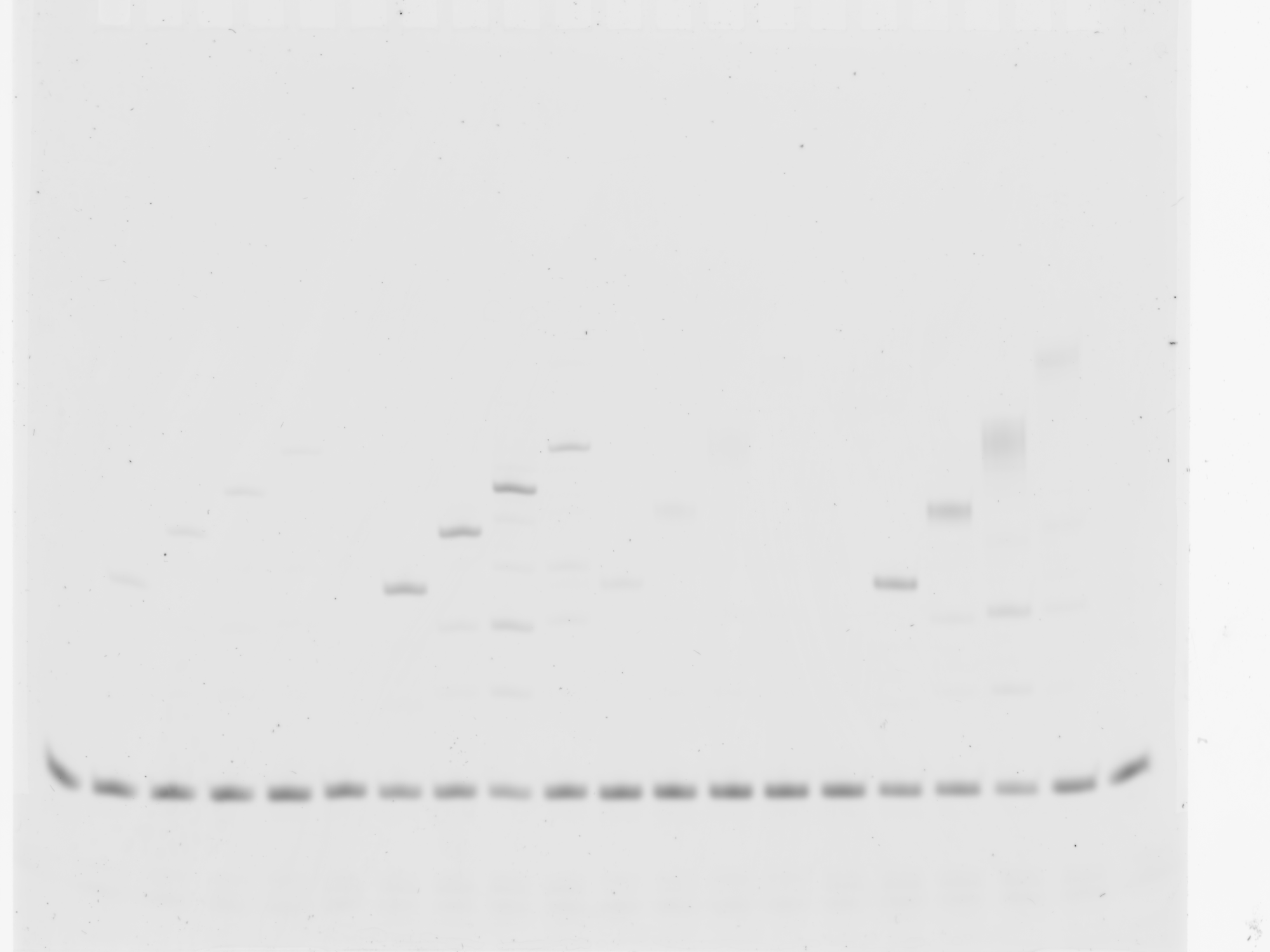

Supplement: Supplementary file 4 — Uncropped gels for Fig. 3b and 3d and graphed values for Fig. 3c and 3e. [file 41557_2025_1830_MOESM4_ESM.zip › SD_Fig3/SD_Fig3d_upper.tif]

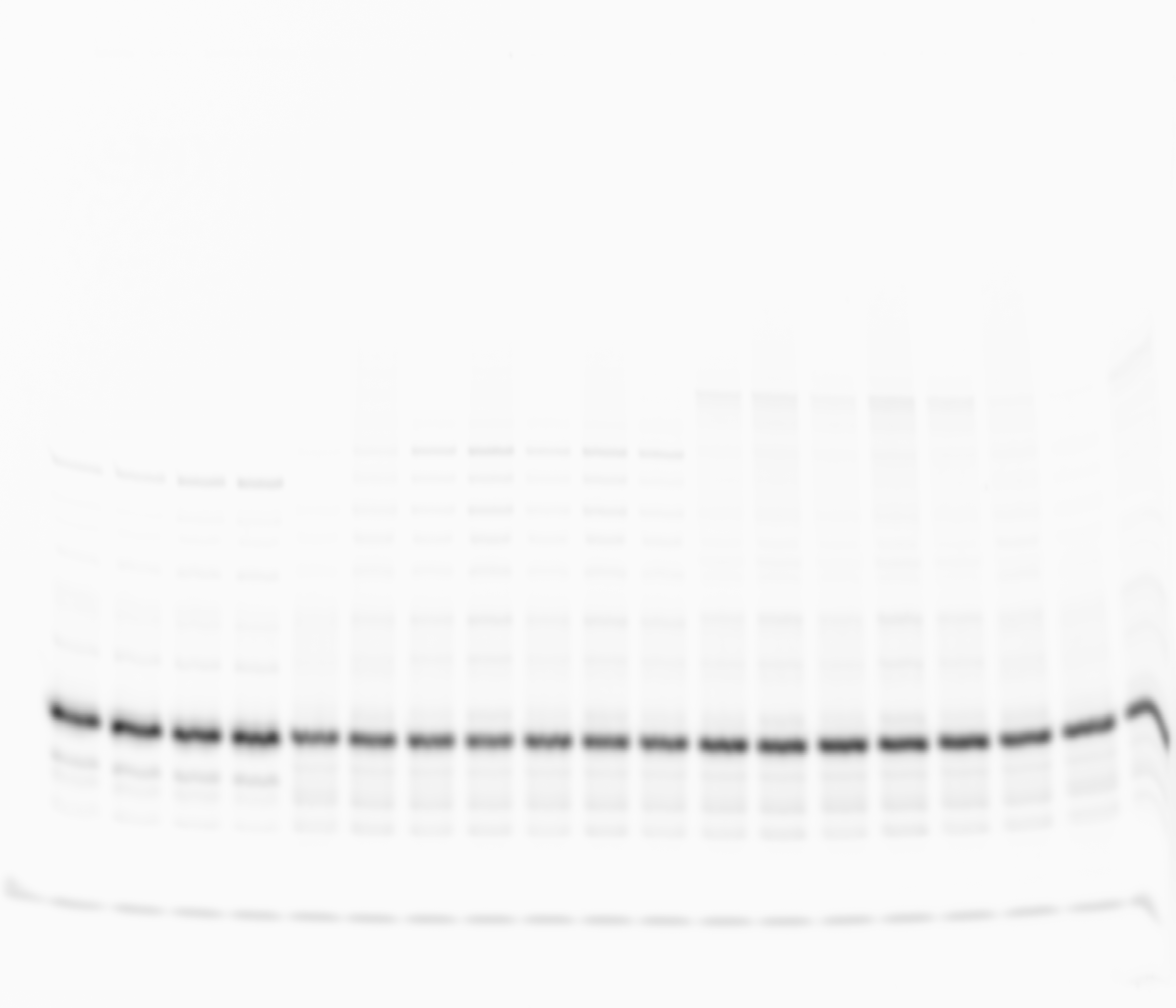

Supplement: Supplementary file 5 — Uncropped gels for Fig. 4b and 4c and graphed values for Fig. 4c. [file 41557_2025_1830_MOESM5_ESM.zip › SD_Fig4/SD_Fig4b_lower.tif]

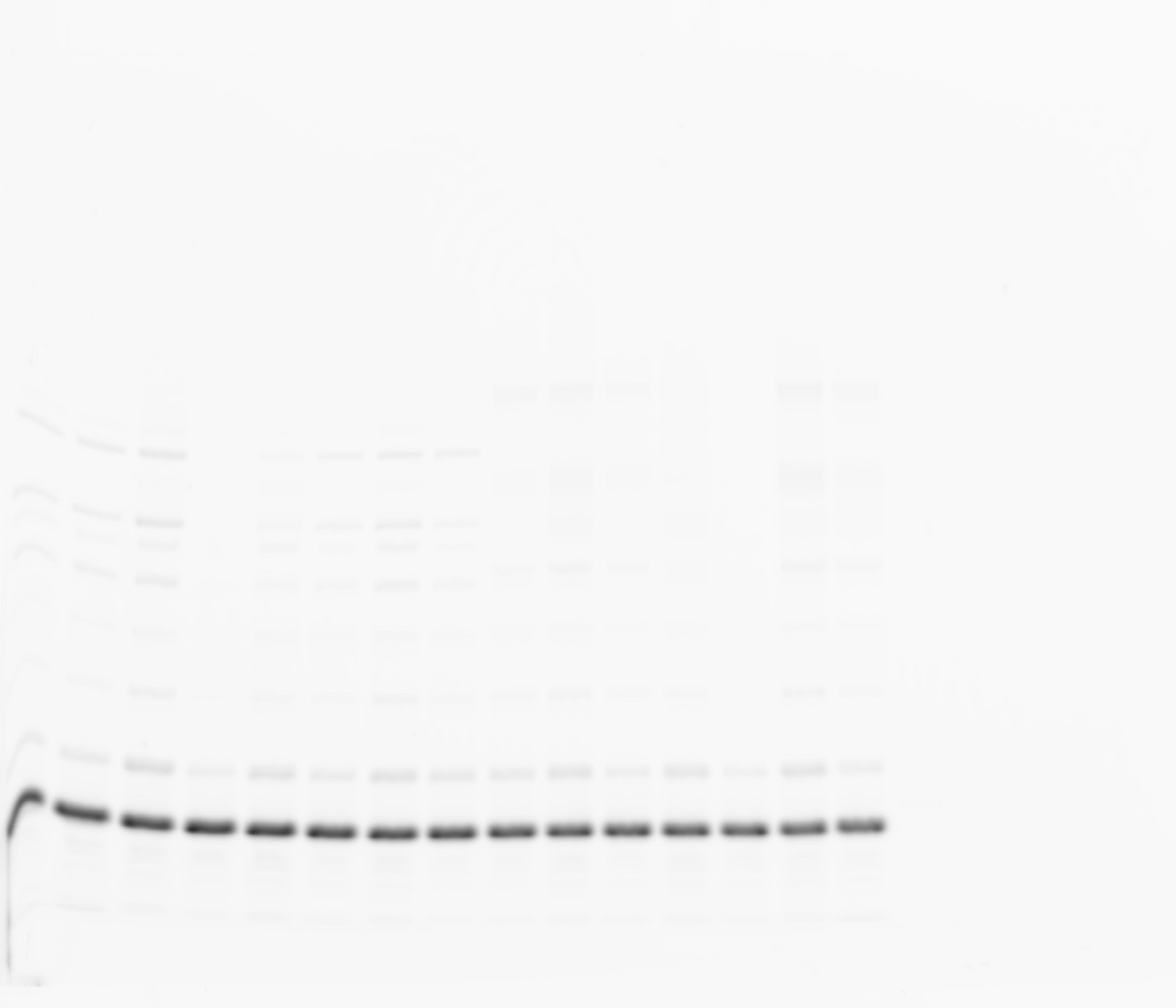

Supplement: Supplementary file 5 — Uncropped gels for Fig. 4b and 4c and graphed values for Fig. 4c. [file 41557_2025_1830_MOESM5_ESM.zip › SD_Fig4/SD_Fig4b_upper.tif]

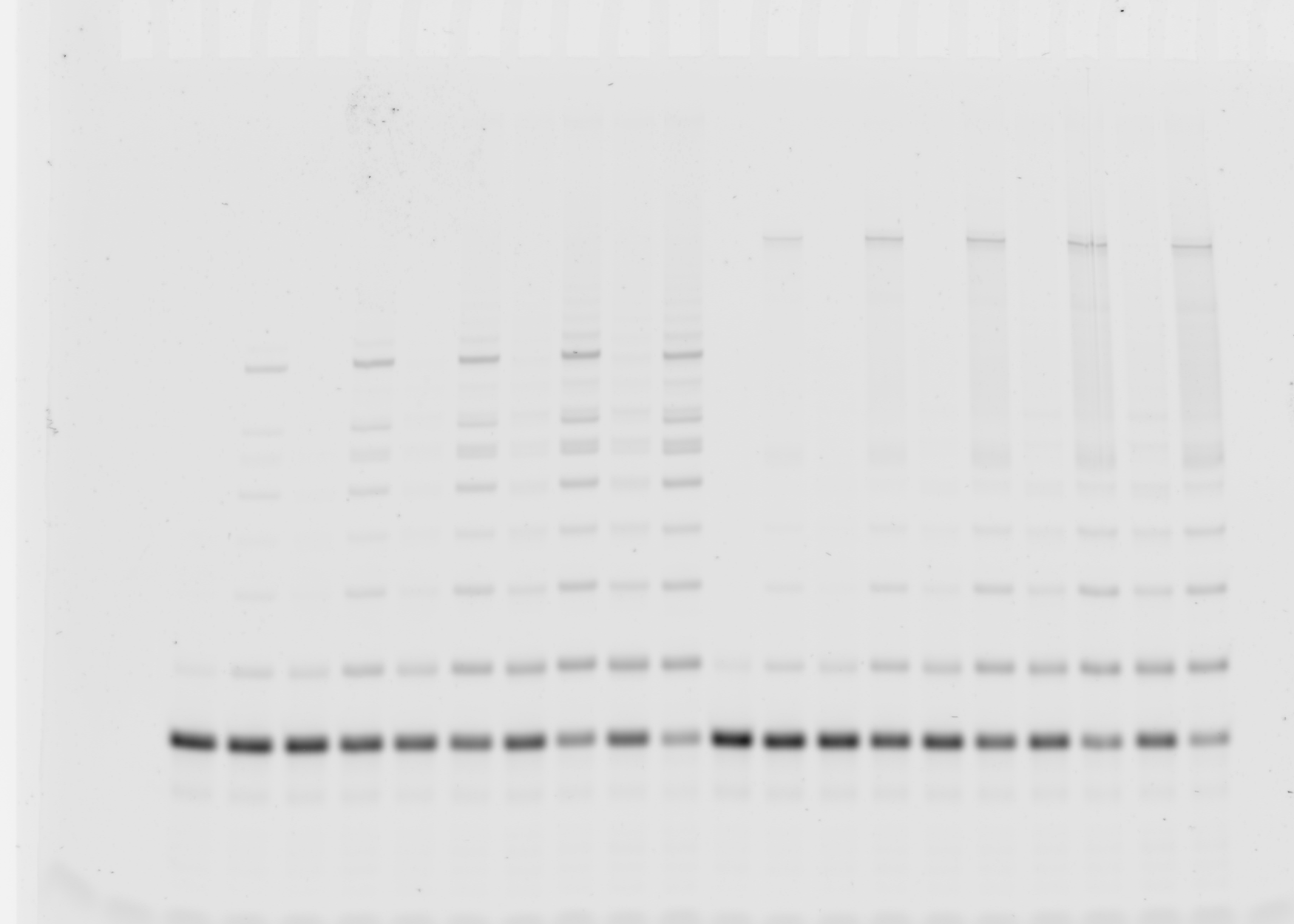

Supplement: Supplementary file 5 — Uncropped gels for Fig. 4b and 4c and graphed values for Fig. 4c. [file 41557_2025_1830_MOESM5_ESM.zip › SD_Fig4/SD_Fig4c_left.tif]

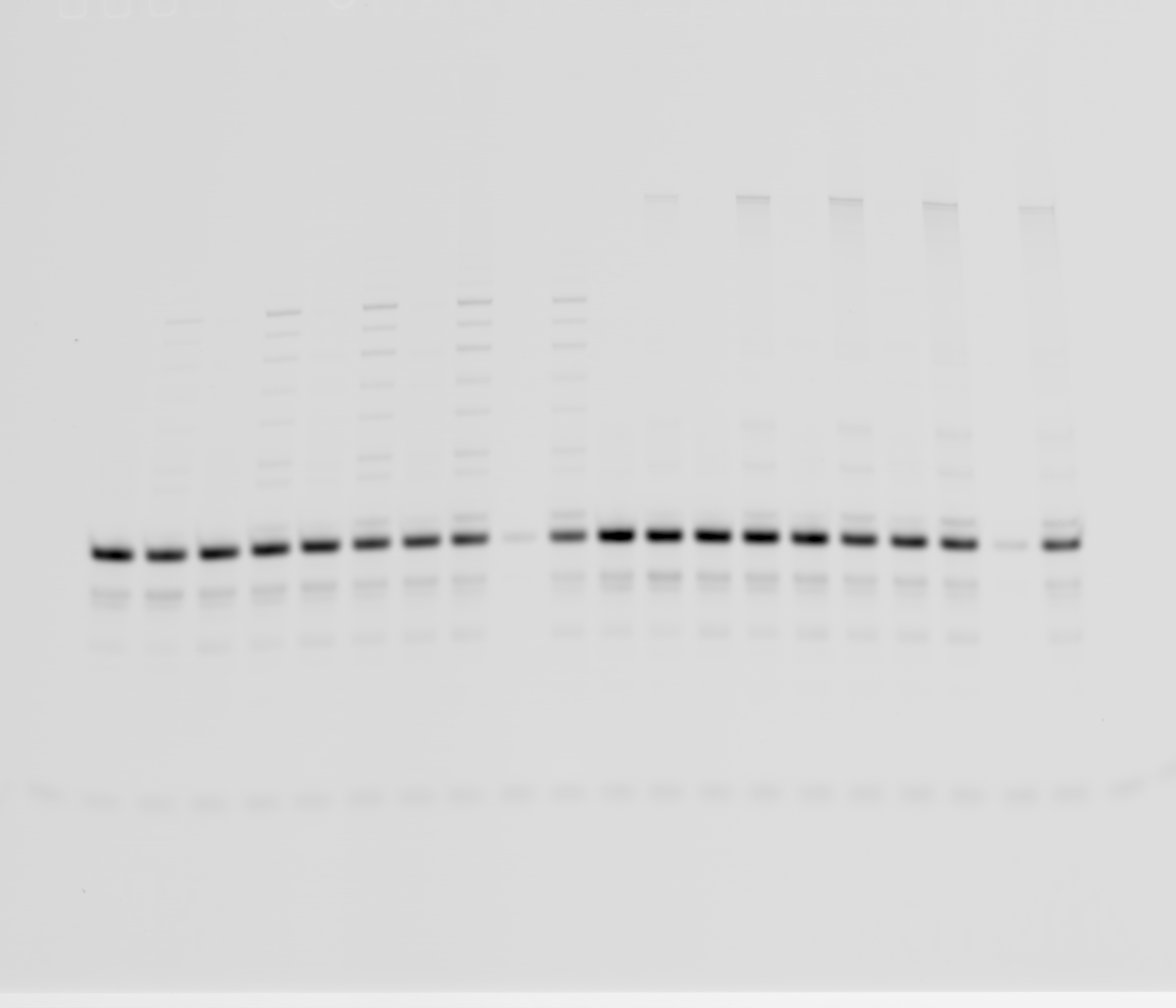

Supplement: Supplementary file 5 — Uncropped gels for Fig. 4b and 4c and graphed values for Fig. 4c. [file 41557_2025_1830_MOESM5_ESM.zip › SD_Fig4/SD_Fig4c_right.tif]

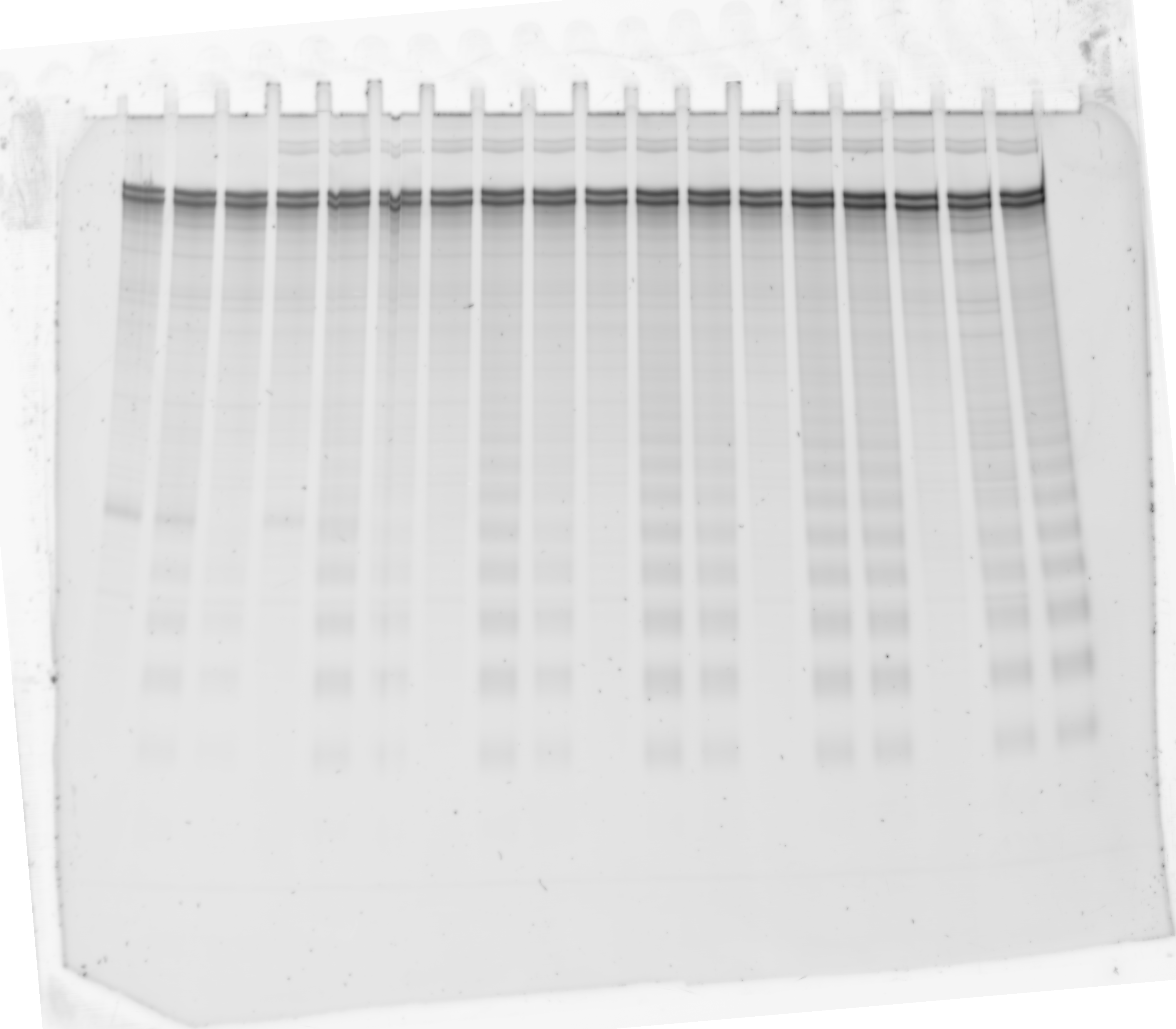

Supplement: Supplementary file 6 — Uncropped gel for Fig. 5b, graphed values for Fig. 5c and charted values for Fig. 5d. [file 41557_2025_1830_MOESM6_ESM.zip › SD_Fig5/SD_Fig5b.tif]

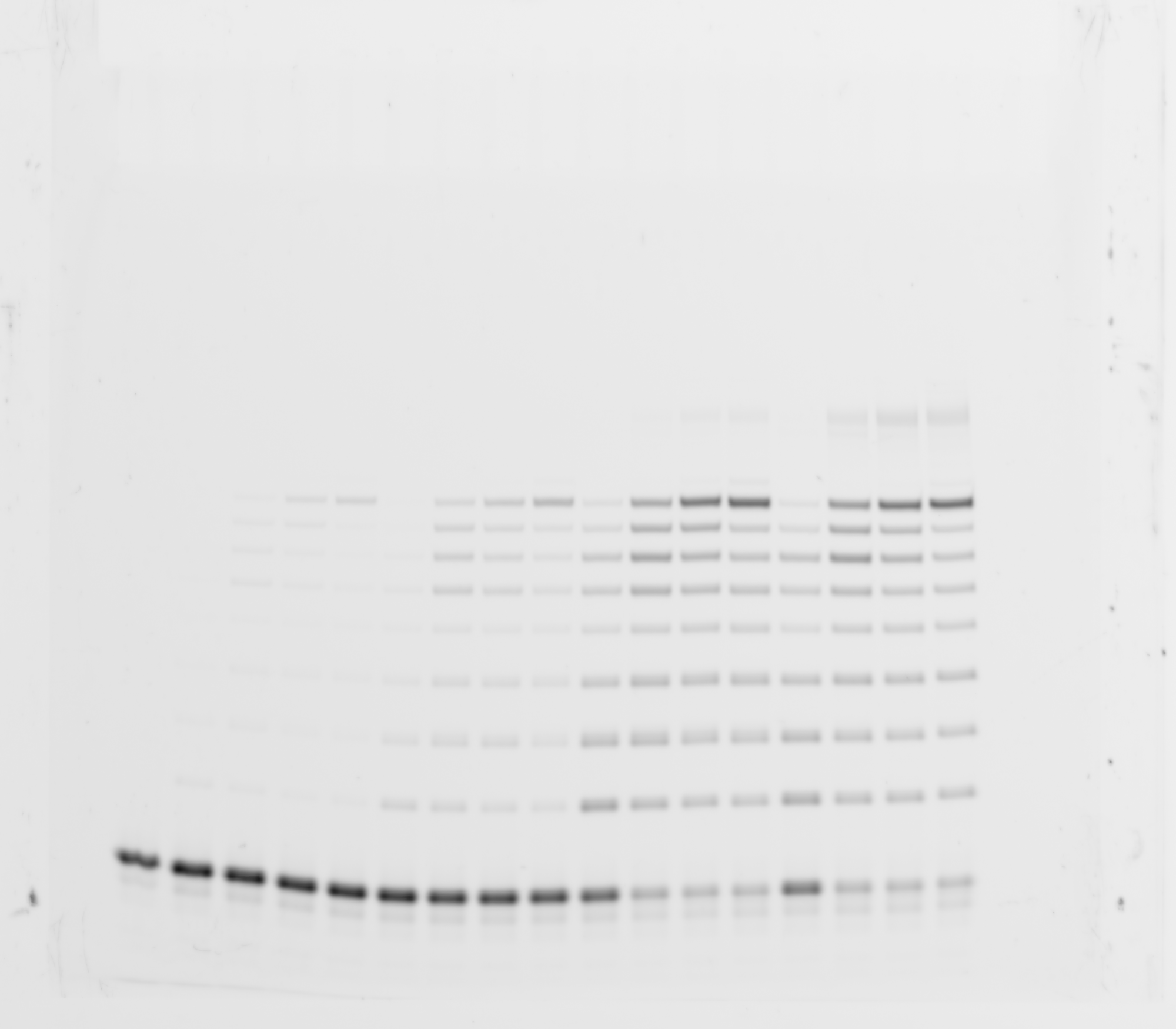

Supplement: Supplementary file 7 — Uncropped gel for Extended Data Fig. 1b. [file 41557_2025_1830_MOESM7_ESM.zip › SD_ExtendedData_Fig1/SD_ExtendedData_Fig1.tif]

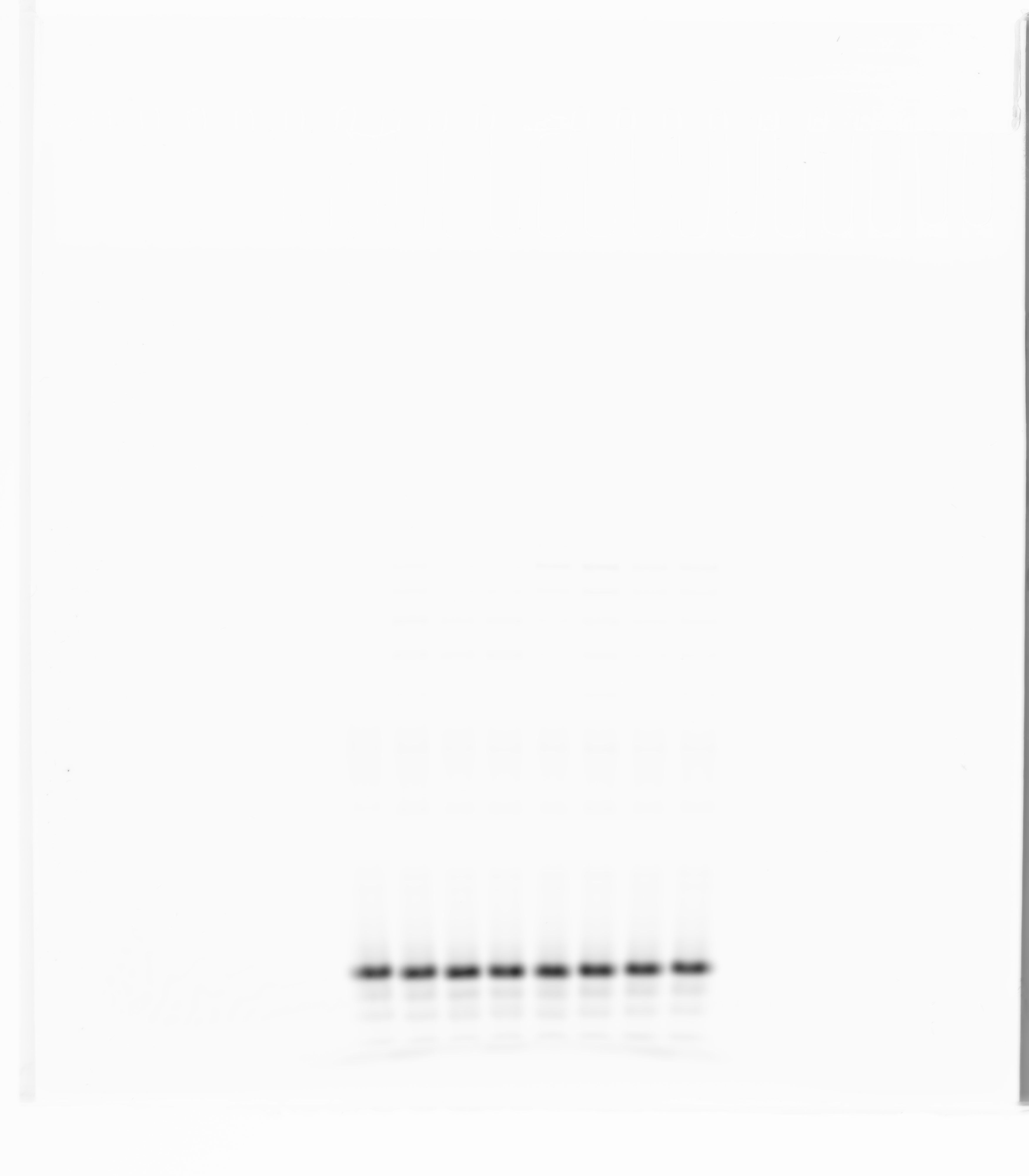

Supplement: Supplementary file 8 — Graphed values for Extended Data Fig. 2b and uncropped gel for Extended Data Fig. 2c. [file 41557_2025_1830_MOESM8_ESM.zip › SD_ExtendedData_Fig2/SD_ExtendedData_Fig2c.tif]

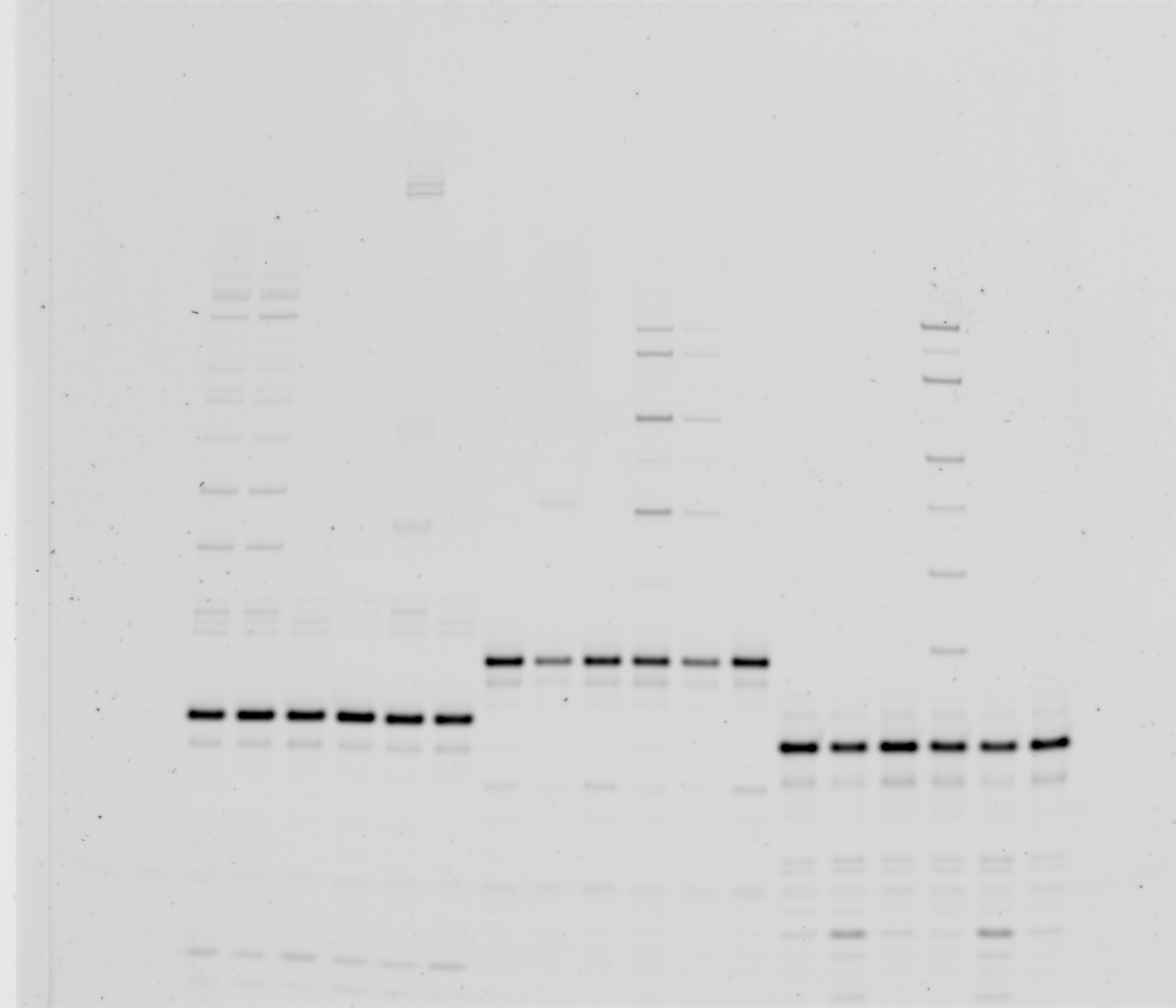

Supplement: Supplementary file 9 — Uncropped gel for Extended Data Fig. 3. [file 41557_2025_1830_MOESM9_ESM.zip › SD_ExtendedData_Fig3/SD_ExtendedData_Fig3.tif]

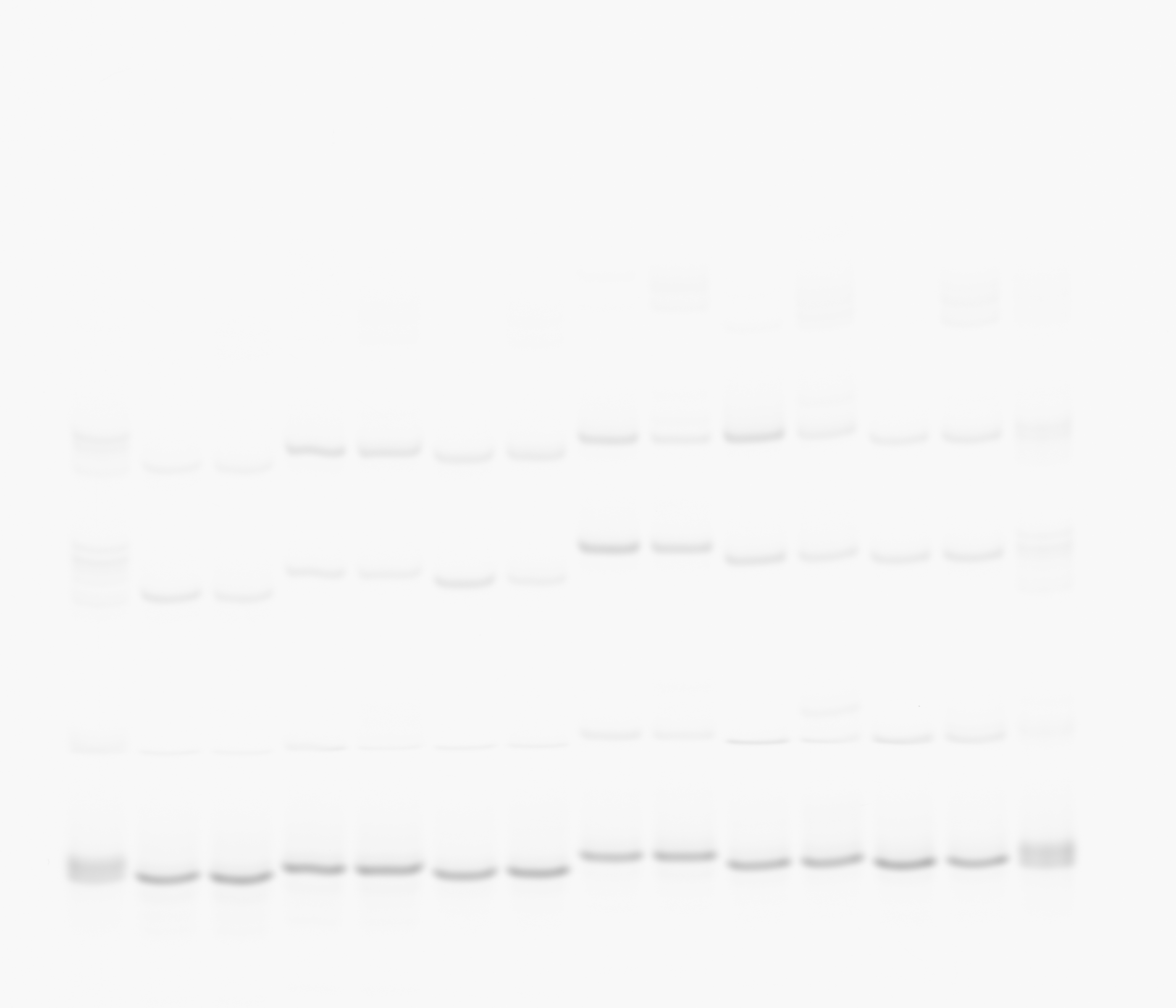

Supplement: Supplementary file 11 — Uncropped gels for Extended Data Fig. 5a and graphed values for Extended Data Fig. 5b. [file 41557_2025_1830_MOESM11_ESM.zip › SD_ExtendedData_Fig5/SD_ExtendedData_Fig5a_left.tif]

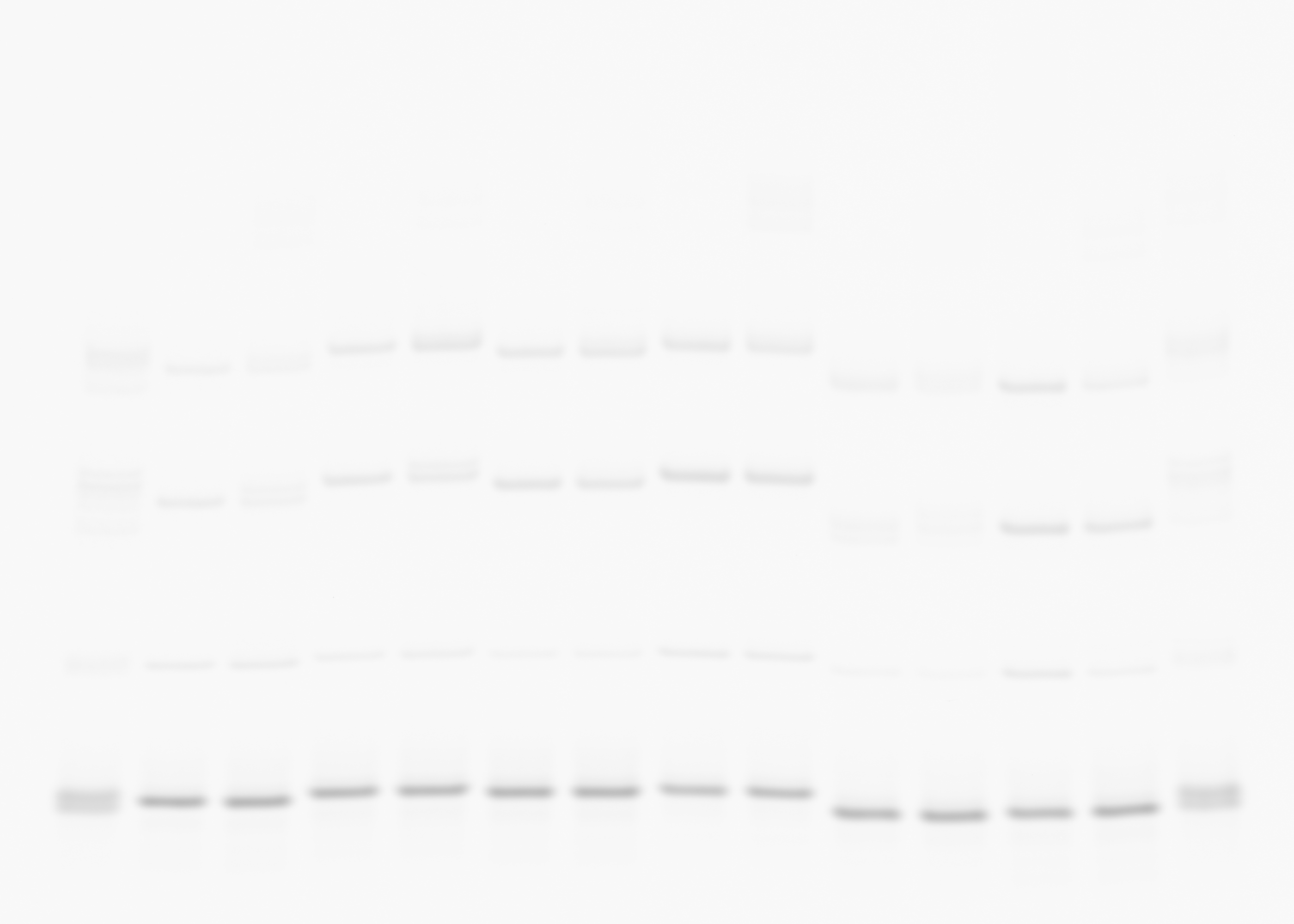

Supplement: Supplementary file 11 — Uncropped gels for Extended Data Fig. 5a and graphed values for Extended Data Fig. 5b. [file 41557_2025_1830_MOESM11_ESM.zip › SD_ExtendedData_Fig5/SD_ExtendedData_Fig5a_right.tif]

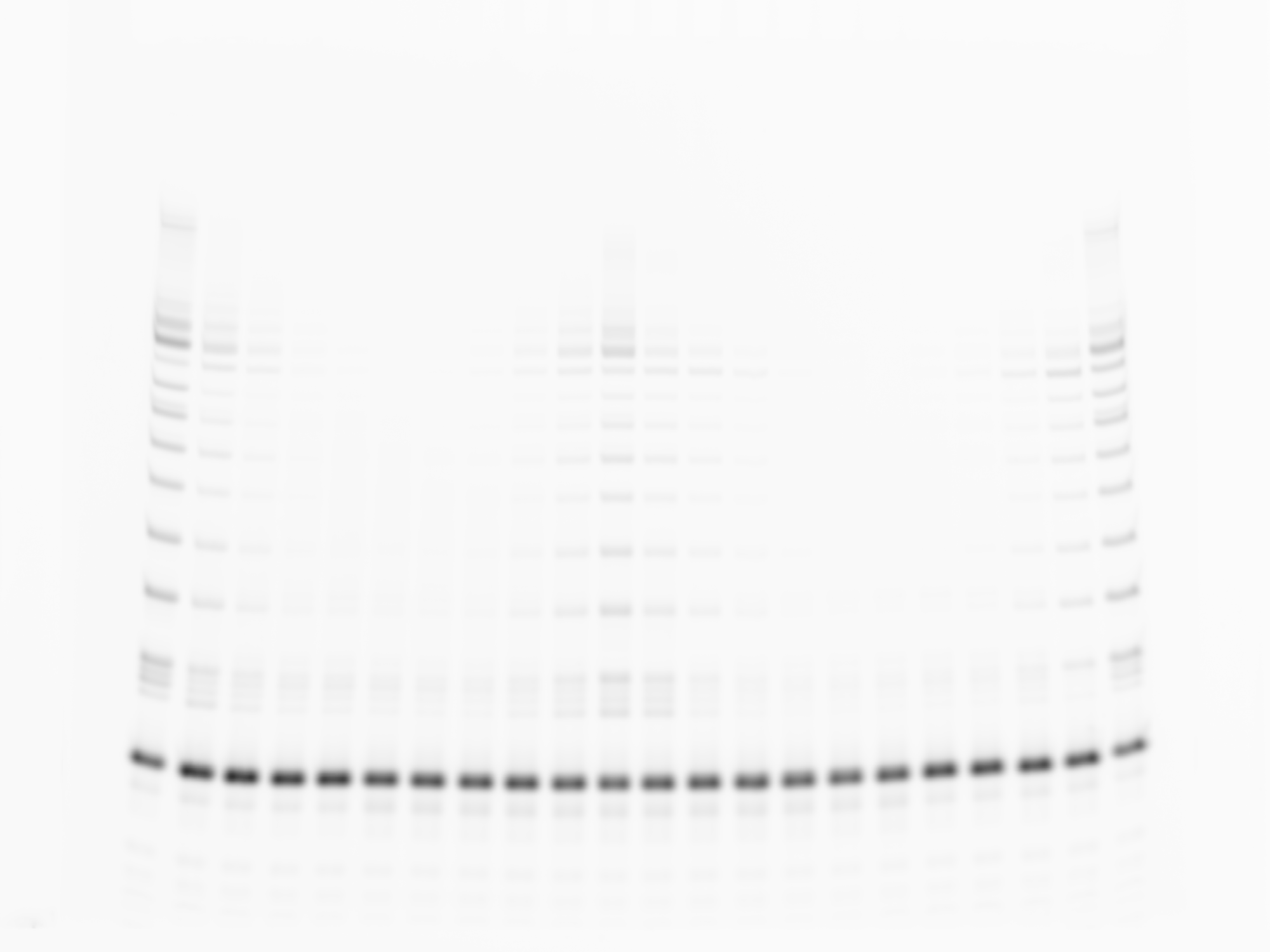

Supplement: Supplementary file 12 — Uncropped gel and graphed values for Extended Data Fig. 6. [file 41557_2025_1830_MOESM12_ESM.zip › SD_ExtendedData_Fig6/SD_ExtendedData_Fig6.tif]

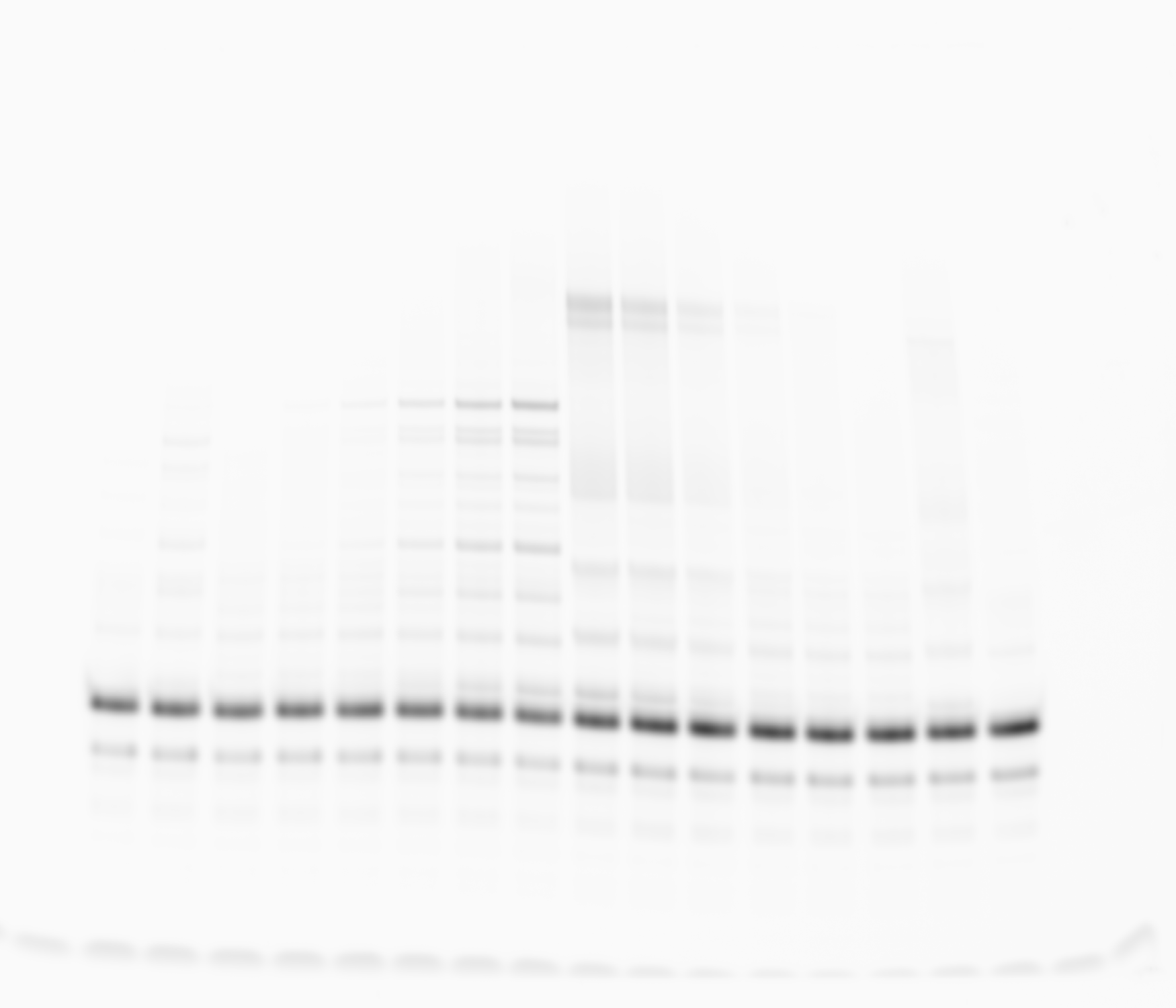

Supplement: Supplementary file 13 — Uncropped gels and graphed values for Extended Data Fig. 7. [file 41557_2025_1830_MOESM13_ESM.zip › SD_ExtendedData_Fig7/SD_ExtendedData_Fig7_lowerleft.tif]

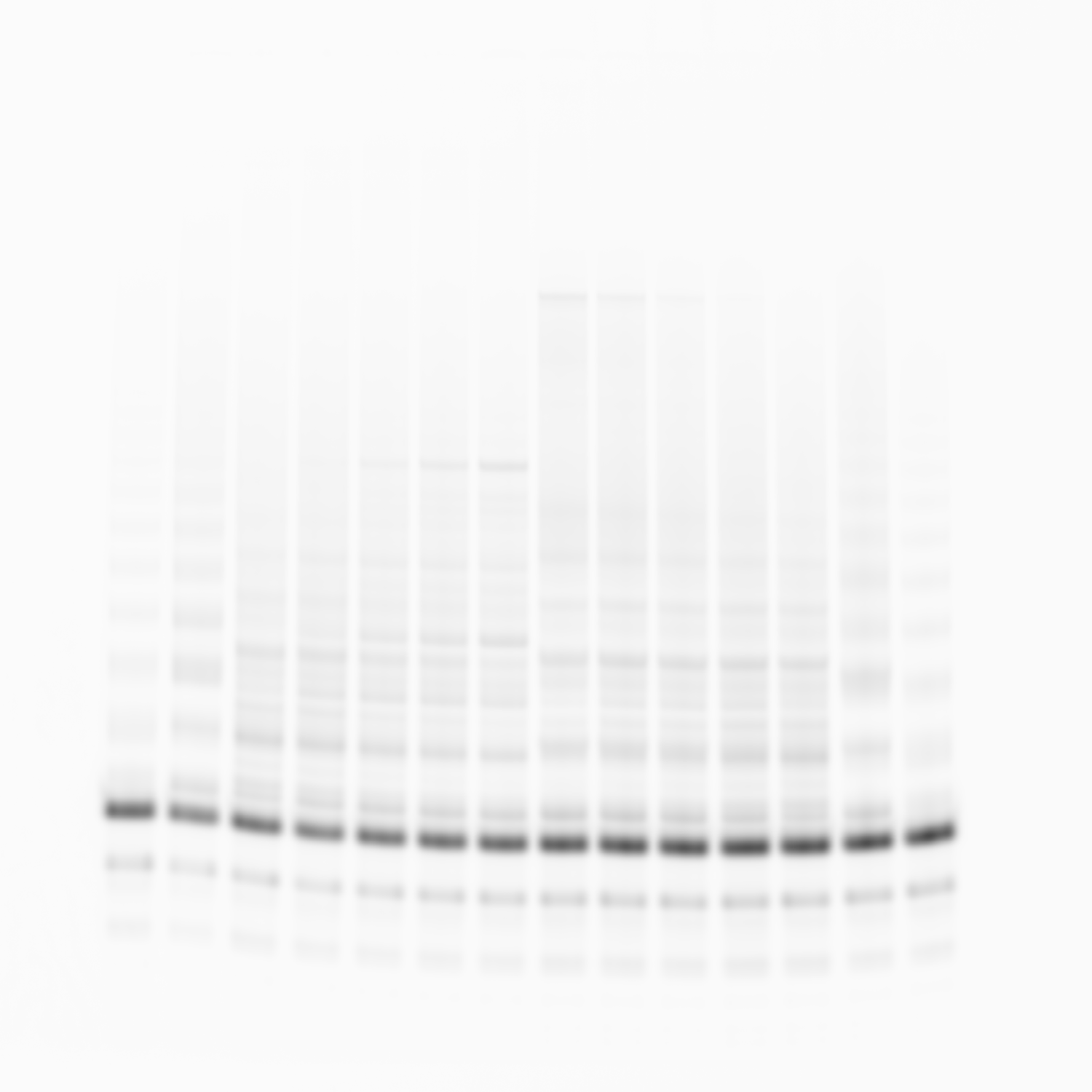

Supplement: Supplementary file 13 — Uncropped gels and graphed values for Extended Data Fig. 7. [file 41557_2025_1830_MOESM13_ESM.zip › SD_ExtendedData_Fig7/SD_ExtendedData_Fig7_lowerright.tif]

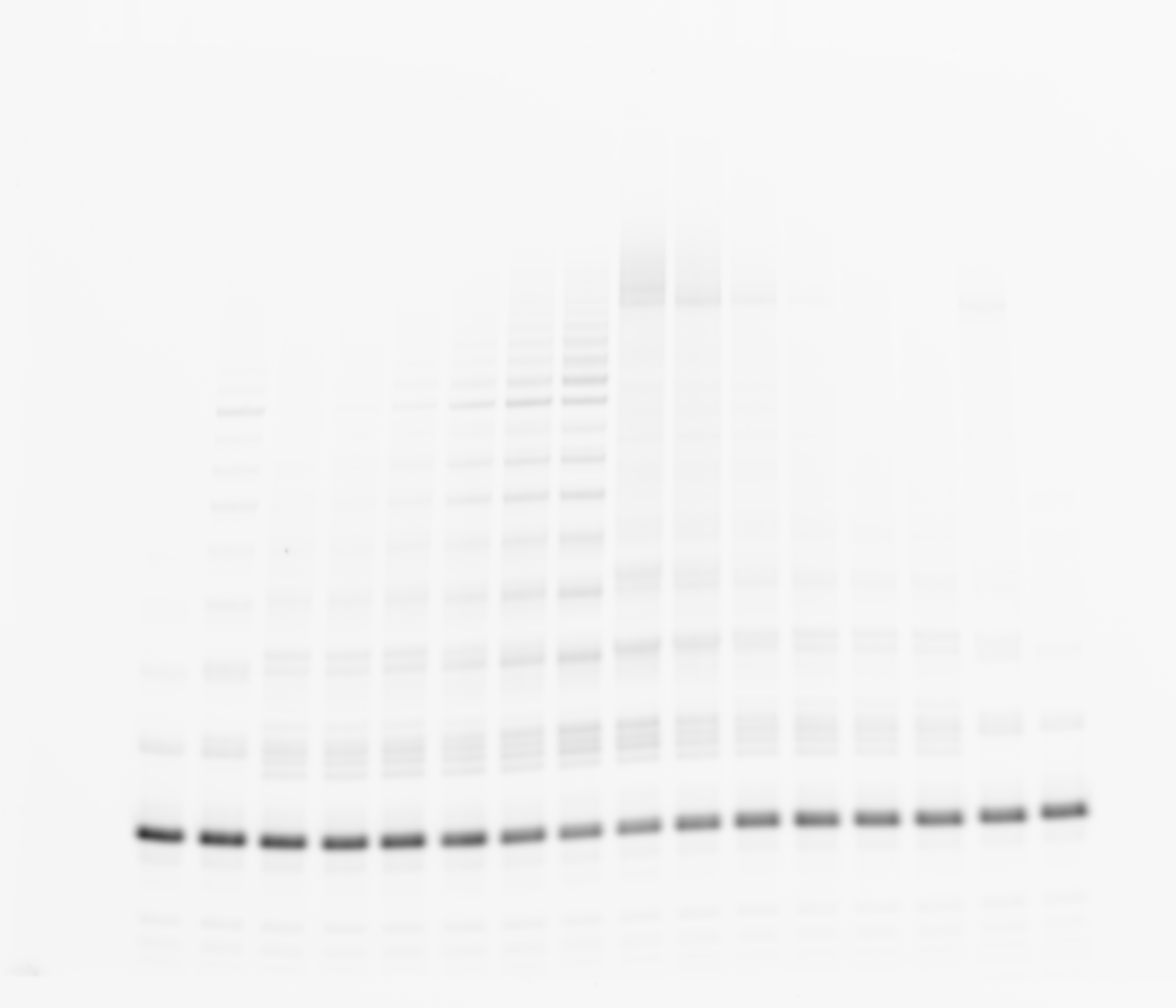

Supplement: Supplementary file 13 — Uncropped gels and graphed values for Extended Data Fig. 7. [file 41557_2025_1830_MOESM13_ESM.zip › SD_ExtendedData_Fig7/SD_ExtendedData_Fig7_upperleft.tif]

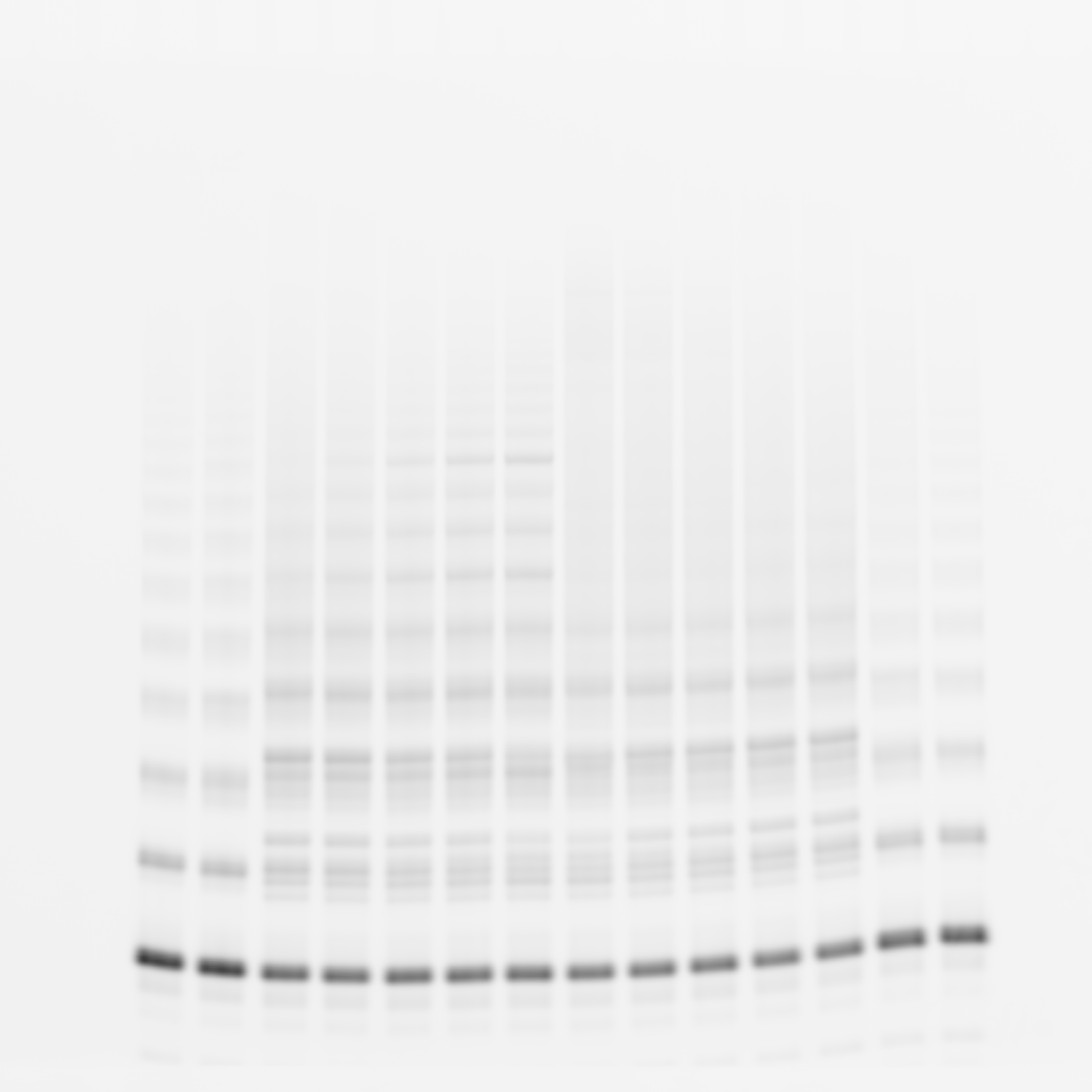

Supplement: Supplementary file 13 — Uncropped gels and graphed values for Extended Data Fig. 7. [file 41557_2025_1830_MOESM13_ESM.zip › SD_ExtendedData_Fig7/SD_ExtendedData_Fig7_upperright.tif]

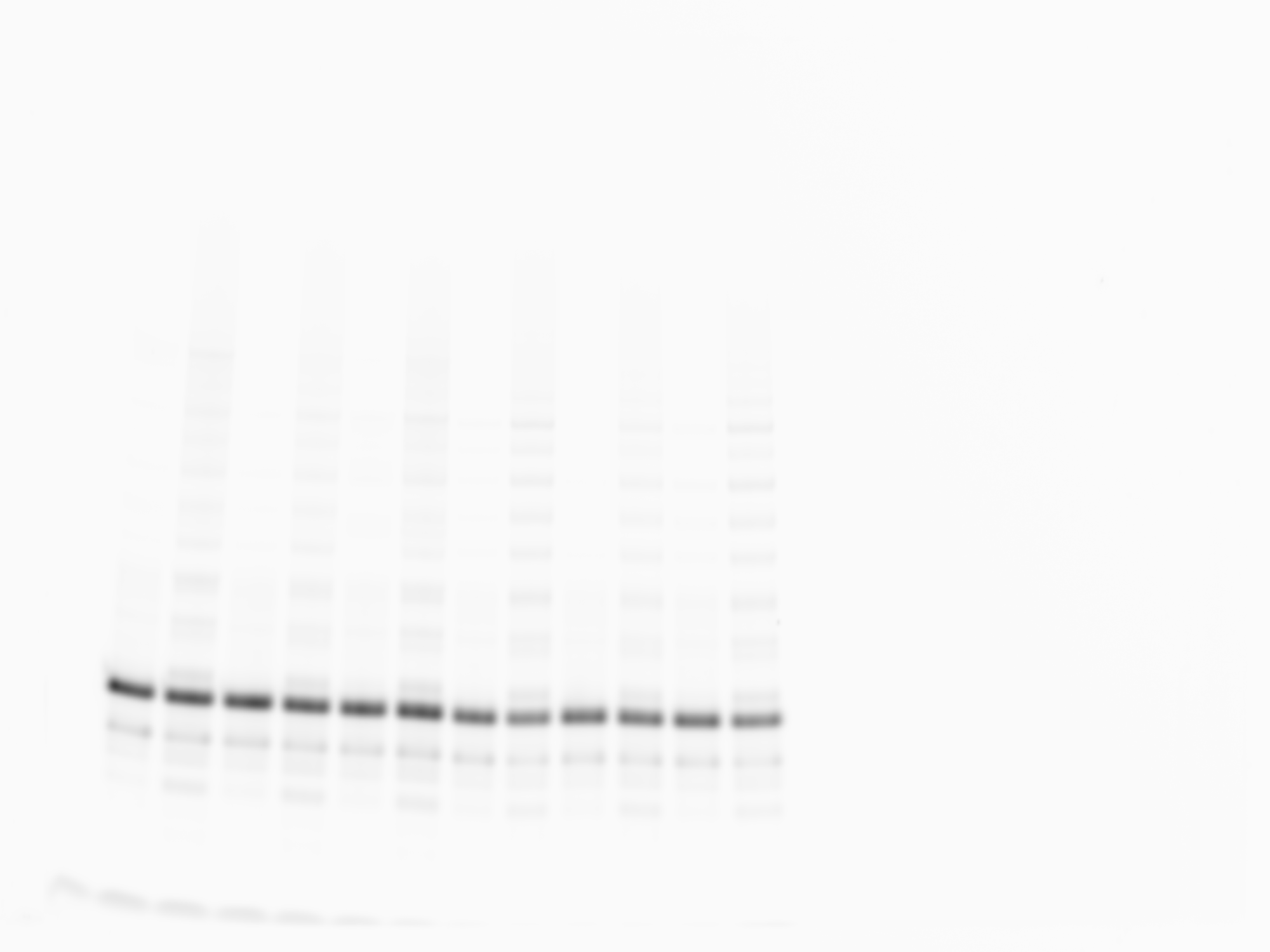

Supplement: Supplementary file 14 — Uncropped gels for Extended Data Fig. 8a and charted values for Extended Data Fig. 8b. [file 41557_2025_1830_MOESM14_ESM.zip › SD_ExtendedData_Fig8/SD_ExtendedData_Fig8a_lower.tif]

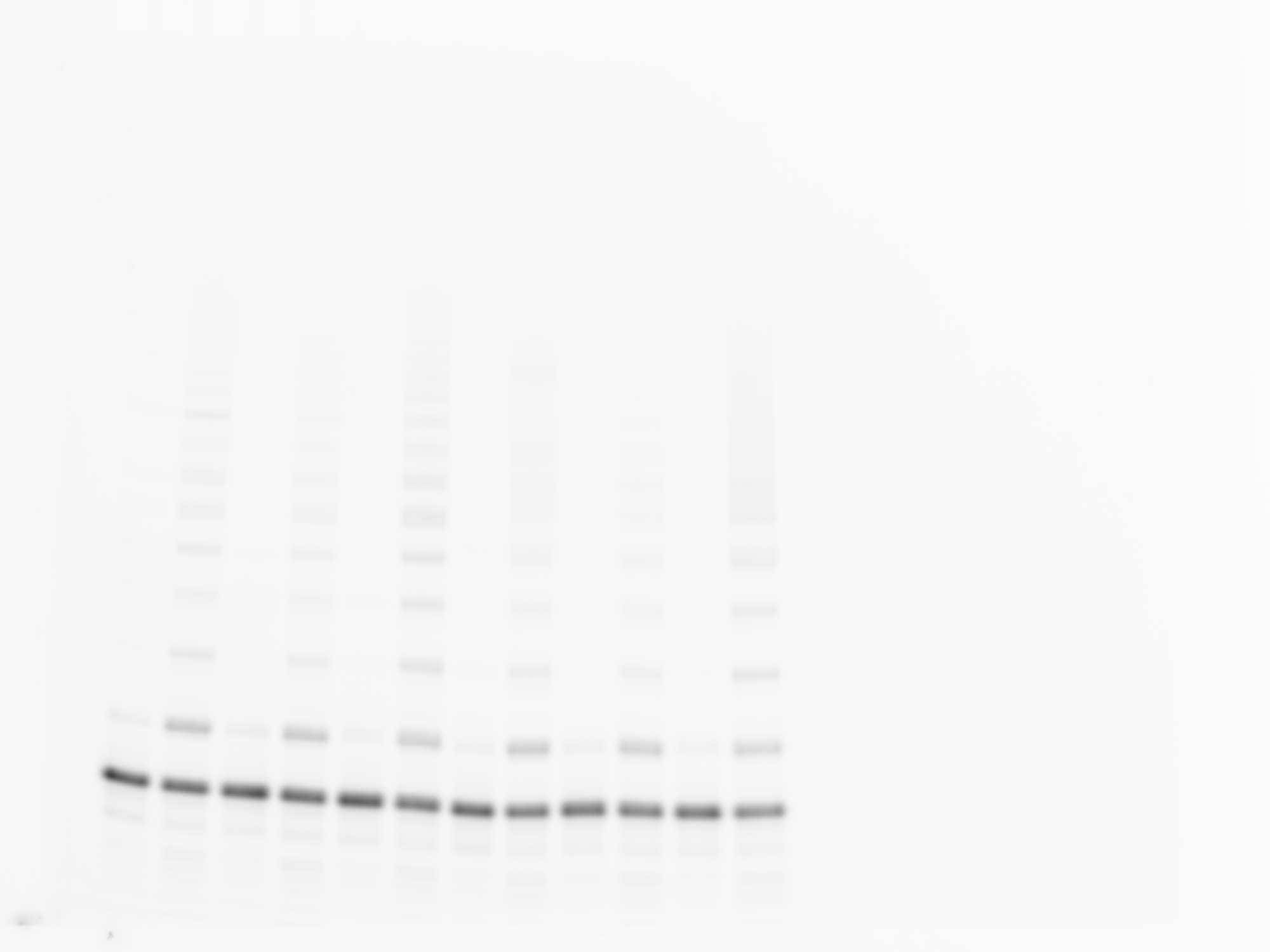

Supplement: Supplementary file 14 — Uncropped gels for Extended Data Fig. 8a and charted values for Extended Data Fig. 8b. [file 41557_2025_1830_MOESM14_ESM.zip › SD_ExtendedData_Fig8/SD_ExtendedData_Fig8a_upper.tif]
